# Quantitative analysis of a rare disease network’s international contact database and E-repository provides insights into biobanking in the electronic consent era

**DOI:** 10.1186/s13023-019-1145-y

**Published:** 2019-07-11

**Authors:** Alexander Suarez, Curran Reilly, David C. Fajgenbaum

**Affiliations:** 1 Perelman School of Medicine, University of Pennsylvania, Philadelphia, PA USA; 2 Castleman Disease Collaborative Network, Philadelphia, PA USA; 3 Division of Translational Medicine and Human Genetics, Perelman School of Medicine, University of Pennsylvania, Philadelphia, PA USA

**Keywords:** Castleman disease, Biorepository, Electronic consent, Contact database, Electronic repository, Enrollment strategy

## Abstract

**Background:**

Castleman disease (CD) describes a group of rare and poorly understood lymphoproliferative disorders that include unicentric CD (UCD), Human Herpes Virus-8 (HHV8)-associated multicentric CD (HHV8 + MCD), and HHV8-negative/idiopathic MCD (iMCD). Efforts to advance research and drug discovery for CD have been slowed by challenges shared by other rare diseases, such as collecting and centralizing data and biospecimens for research. To collect disease characteristic data and identify individuals interested in contributing biospecimens for research, a global research organization - the Castleman Disease Collaborative Network (CDCN) - established an international Contact Database and electronic repository (E-repository). Herein, we performed analyses of these datasets to further characterize CD and gain insights into research biospecimen acquisition.

**Results:**

Descriptive statistical analyses were performed on 891 participants from the Contact Database and 166 patients in the E-repository. The median age of patients at the time of enrollment in the Contact Database and E-repository was 42 ± 15.7 and 35 ± 14.8, respectively. The E-repository had increased representation from patients with MCD and the iMCD subtype compared to other sub-groups. Though the majority of participants were from the USA, a total of 49 countries on 6 continents were represented. Several patient characteristics in the Contact Database were associated with subsequent enrollment in the E-repository. There were significantly more MCD patients (*p* < 0.0001) and females (*p* = 0.002) enrolled in the E-repository compared to the Contact Database. Patient’s year of birth, date of registration, preferred method of communication, and relationship to the patient were also significantly associated with enrollment in the e-Repository.

**Conclusions:**

This study of the largest- dataset of CD patients worldwide provides insights into disease phenotypes, characteristics of patients interested in contributing data and biospecimens for research, and methods for successfully acquiring data and biospecimens. Generally, the factors associated with enrollment in the E-repository represented severity of disease subtype, proximity to the research, and patient motivation. We hope that these findings and the sample documentation (e.g., electronic consent, recruitment materials) provided with this article will assist future rare disease efforts with overcoming hurdles.

**Electronic supplementary material:**

The online version of this article (10.1186/s13023-019-1145-y) contains supplementary material, which is available to authorized users.

## Background

In the United States, a rare or orphan disease is defined as any disease affecting fewer than 200,000 people [1]. Collectively, 1 in 10 Americans suffer from one of the approximately 7000 rare diseases; only 5% currently have FDA-approved treatments [2]. Rare disease research faces many challenges that impede discoveries and new drug development, including decreased priority, focus, or funding relative to more common diseases. Due to the nature of rare diseases - few cases worldwide, complicated diagnoses, restricted availability of information and resources – accessing necessary biospecimens and patient data pose additional challenges.

Castleman disease (CD) is a group of rare and poorly understood lymphoproliferative disorders. Clinicians and researchers have primarily relied on case reports to understand CD, as limited access to samples and data has resulted in studies with small numbers. While all cases of CD share common lymph node histopathological features, CD is further sub-classified into: Unicentric CD (UCD), Human Herpesvirus-8 (HHV-8)-associated multicentric CD (HHV8 + MCD), and HHV-8-negative/idiopathic MCD (iMCD) [3]. UCD involves a single region of enlarged lymph nodes and mild symptoms, which can be resolved with lymph node resection. HHV8 + MCD involves multiple regions of enlarged lymph nodes and symptoms related to intense immune hyperactivation due to uncontrolled infection with HHV-8. iMCD is characterized by nearly identical clinical and laboratory abnormalities as HHV8 + MCD, but these patients are HHV-8 negative and the etiology is unknown. Historically iMCD has received the least research attention. Unsurprisingly, it is also the least well understood and most deadly subtype. A review of all published case of MCD was performed in 2016 to further investigate and define iMCD [4]. 42% of patients were found to be HHV-8-positive; 25% had unknown HHV8 status; and 33% were HHV-8-negative. This study provided important insights into clinical and pathological features of iMCD, but publication bias could not be accounted for as every case had been previously written up.

In 2012, the Castleman Disease Collaborative Network (CDCN) was created to advance research and treatment discovery for CD. The CDCN forged a novel framework for research called the collaborative network approach to promote collaboration, prioritize and fund high impact research, facilitate tissue and data sharing for research, and support patients [5]. In order to facilitate tissue and data sharing for research, the CDCN launched an international Contact Database in July 2014 and an electronic repository (E-repository) in July 2015. The Contact Database collects a limited set of data on patients and loved ones; the E-repository enables patients to indicate their interest in donating tissue samples for research.

Our aim is to characterize the largest dataset of CD patients in the Contact Database and to compare the features of patients who also sign up for the E-repository against those who do not. We also share resources, including a patient-directed electronic consent (e-consent) and marketing materials, that can be re-used by other rare disease efforts that are interested in improving research biospecimen acquisition.

## Methods

### Patient databases

The CDCN webpages for participants to sign up for the Contact Database and the E-repository are shown in Additional file 1: A. The Contact Database includes an online questionnaire regarding participant demographic data, diagnosis, contact information, and preferences for getting more involved. Following completion of the Contact Database form, the participant receives an automated email that includes a link to sign up for the E-repository, among other links (Additional file 1: B1-B2). Within 24 h, the participant also receives a personalized follow-up email from a CDCN staff member, which also includes a link to sign up for the E-repository.

The E-repository includes a Tissue Sample Donation Interest Form (TSDIF) to collect more in depth information regarding the patient’s demographics, course of disease, past medical history, and any previous tissue donation (See Additional file 1: C for full list of questions). Completion of this form to express interest in participating in research is included as part of a protocol (“The Castleman Disease Collaborative Network Biobank: A Collection of Biospecimens and Clinical Data to Facilitate Research”) approved by Quorum IRB. A separate electronic consent form was developed for the CDCN’s Biobank (see Additional file 1: D). Participants can request to be removed from the Contact Database and E-repository at any time.

### Statistical analyses

Descriptive statistical analyses were performed on the demographic and disease characteristic data for both databases. Not all patients completed each registration question for the Contact Database and E-repository form. Chi-square analysis was performed to search across the two databases for significant associations between characteristics of patients in the Contact Database and likelihood of joining the E-repository. The Bonferroni method of multiple hypothesis testing was performed to determine corrected *p*-values. Alpha was determined to be *p*-value < 0.05.

## Results

**Fig. 1:**
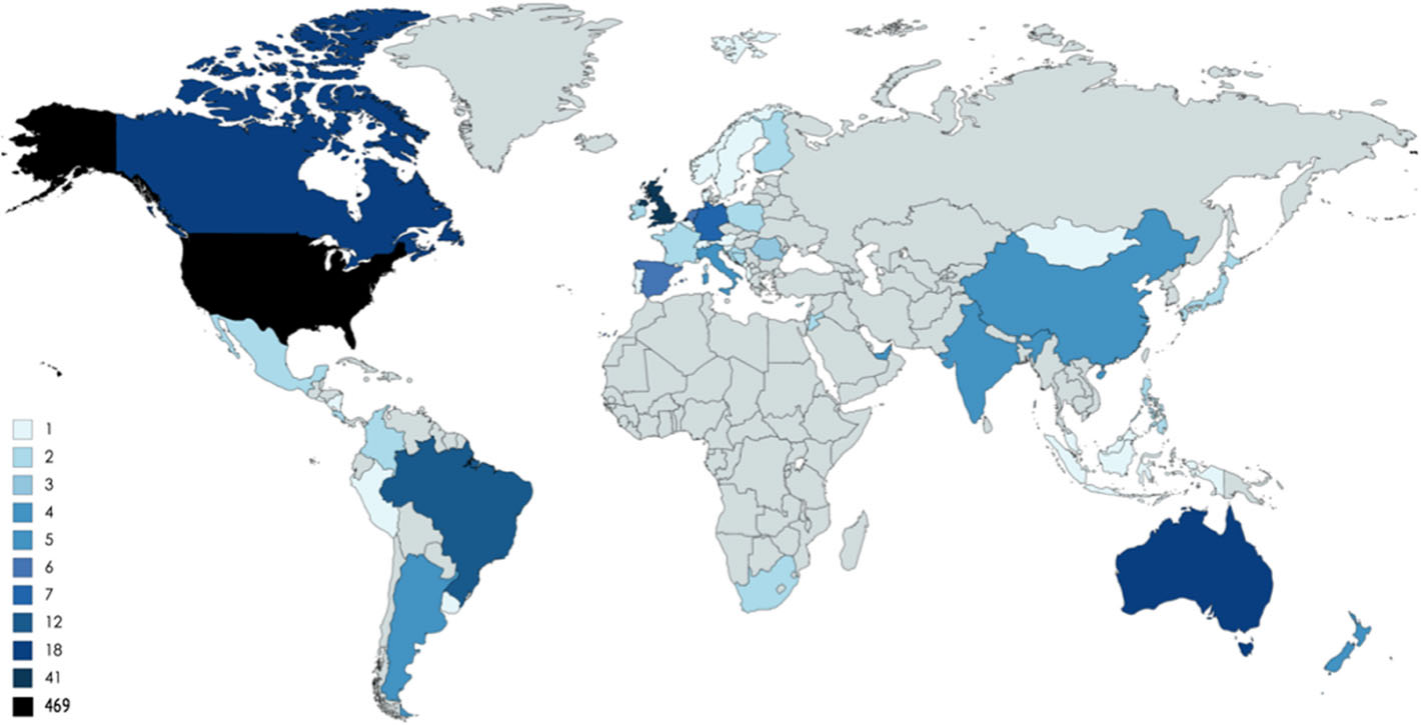
Distribution of Patients in Contact Database by Country (*n* = 654)

The Contact Database includes 891 patients enrolled between July 2014–December 2017, and the E-repository includes 166 patients enrolled between July 2015–December 2017. There are 165 patients in both the Contact Database and the E-repository; one patient is in the E-repository but not the Contact Database. Table 1 includes demographics of patients in the Contact Database and E-repository. Of those participants registered in the Contact Database (*n* = 891), 18.2% also registered for the E-repository (*n* = 165). In the Contact Database, 49% of patients were female and 51% of patients were male; 72% of patients were from the US. The remaining 28% of participants represent 49 countries on 6 continents (Fig. 1). The mean age of patients at the time of enrollment was 41.8 years old. The proportion of patients with CD subtypes included 41% MCD, 33% UCD, and 26% unknown. Sixty percent of MCD patients were female and 69% of UCD patients were female (Table 2). Among MCD cases, 57% were reported to be HHV-8-negative/iMCD, 10% HHV8 + MCD, and 33% unknown.

**Table 1:** Demographic data for patients enrolled in the Contact Database and E-repository, and prior estimates in literature

Title	Contact Database	E-repository	*p*-value*	Talat & Schulte	Liu et al.
N	891	166		384	128 iMCD
Age (Median ± SD)					
UCD	35 ± 14.8	–		30	–
MCD	42 ± 15.7	–	0.612^1^	52	50
Patient Gender, N (%)			0.002^2^		
Female	200 (49%)	44 (67%)		179 (47%)	54 (42%)
Male	195 (51%)	22 (33%)		205 (53%)	74 (58%)
Patient Country of Origin, N (%)			0.002^2^		
US	469 (72%)	117 (82%)		–	–
International	185 (28%)	26 (18%)		–	–
Primary Diagnosis, N (%)			< 0.0001^2^		
Multicentric CD	206 (41%)	59 (51%)		101 (26%)	128
Unicentric CD	168 (33%)	49 (42%)		283 (74%)	–
Unknown	131 (26%)	8 (7%)		–	–
Patient’s MCD Subtype Diagnosis, N (%)			0.403^2^		
HHV-8-negative	117 (57%)	38 (64%)		–	128
HHV-8-positive	21 (10%)	5 (8%)		–	–
Not sure or N/A	67 (33%)	16 (27%)		–	–

^1^Student’s t-test *p*-value ^2^ Chi square *p*-value *p-value with Bonferroni correction (k = 21), a = .002

**Table 2:** Patient-reported subtype of CD by gender in the Contact Database

Title	Male	Female
Primary Diagnosis, N (%)		
Multicentric CD	81 (40%)	120 (60%)
Unicentric CD	38 (31%)	86 (69%)

Patients in the E-repository were 67% female and 82% were from the US. The mean age of patients was 42.6 years old. Fifty-one percent of patients reported having MCD compared to 42% UCD and 7% unknown. Sixty-four percent of MCD cases reported having HHV-8-negative/iMCD, 8% HHV8 + MCD, and 27% unknown.

In order to identify features significantly associated with enrollment in the E-repository, differences in the characteristics of patients in the Contact Database and E-repository were evaluated (Table 3). The distribution of patient gender and primary diagnosis was significantly different between patients in the Contact Database and E-repository (corrected *P* < 0.05 after correcting for multiple hypothesis testing). Additional characteristics significantly associated with enrollment in the E-repository included patient’s year of birth, date of registration, preferred method of communication, and participant’s relationship to the patient. Elements that trended towards significance included patient’s country of origin, the participant’s interest in joining a patient engagement program, and the amount of time elapsed between diagnosis and date of enrollment. There were no differences in patients’ subtype of MCD, age at diagnosis, date of diagnosis, and disease specific symptoms between the two cohorts.

**Table 3:** Summary of comparison of characteristics of patients in the Contact Database and E-Repository by Chi Square, displayed in order by *p*-value

Title	*p*-value*
Statistically significant (α < 0.002)	
Patient’s Primary Diagnosis	< 0.0001
Date of Registration	< 0.0001
Participant’s Preferred Method of Communication	< 0.0001
Participant’s Relationship to Patient	< 0.0001
Patient’s Year of Birth	0.001
Patient’s Gender	0.002
Trending towards significance (α = 0.002–0.01)	
Patient’s Country	0.002
Time elapsed between date of diagnosis and date of registration	0.005
Interest in Warrior program	0.007
Not statistically significant (α > 0.01)	
Patient’s Date of Diagnosis	0.075
Patient’s Symptoms	0.177
Patient’s Subtype Diagnosis	0.403
Patient’s Age at Diagnosis	0.499
Participant’s Age	0.502
Participant’s Gender	0.615
Participant’s Year of Birth	0.721
Distance between participant and patient based on zip code	0.742
Registration Date relative to annual Patient Summit	0.851

*p-value with Bonferroni correction (k = 21), α = .002

The demographics of those who elected to register for both the Contact Database and E-repository are summarized in Table 4. Among those who signed up for the Contact Database, women had nearly a two-fold increased rate of joining the E-repository when compared to men (22.3 vs. 10.6%). Patients born either between 1961 and 1970 (27.1%) or 2011–2018 (27.1%), compared to those patients of other age cohorts, were also significantly more likely to enroll. MCD patients (28.6%) and UCD patients (29.2%) were significantly more likely to enroll in the E-repository than participants who were unsure of diagnosis or not officially diagnosed (6.1%). Date of registration for the Contact Database was also significantly associated with enrollment in the E-repository. Patients who registered for the Contact Database between July–December 2014 (25.9%), July–December 2015 (30.4%), and January–June 2016 (25.4%) were more likely to sign up for the E-repository than registrants at other time periods. Of the patients who listed both phone and email as their preferred methods of communication (rather than just one or neither), 100% signed up for the E-repository. When patients registered themselves directly for the Contact Database, they were more likely to also register for the E-repository (27.8%), compared to those patients who were registered by family (4.1%) or friends (3.2%).

**Table 4:** Odds Ratios of joining E-repository for Significant Characteristics

Title	Joined E repository	Did not join	Rate of E-repository	Odds Ratio^a^
Patient’s Gender	*n* = 395			
Male	22	178	11.0%	0.60
Female	44	151	22.6%	1.24
Patient’s Year of Birth	*n* = 721			
1930–1940	0	12	0.0%	0.00
1941–1950	9	25	26.5%	1.45
1951–1960	16	84	16.0%	0.88
1961–1970	48	127	27.4%	1.51
1971–1980	27	124	17.9%	0.98
1981–1990	34	100	25.4%	1.39
1991–2000	5	74	6.3%	0.35
2001–2010	2	19	9.5%	0.52
2011–2018	5	10	33.3%	1.83
Patient’s Primary Diagnosis	*n* = 505			
Multicentric CD	59	147	28.6%	1.57
Unicentric CD	49	119	29.2%	1.60
Unsure or Not officially diagnosed	8	123	6.1%	0.34
Date of Registration	*n* = 874			
Jul-Dec 2014	30	86	25.9%	1.42
Jan-Jun 2015	26	113	18.7%	1.03
Jul-Dec 2015	31	71	30.4%	1.67
Jan-Jun 2016	35	103	25.4%	1.39
Jul-Dec 2016	17	87	16.3%	0.90
Jan-Jun 2017	7	138	4.8%	0.27
Jul-Dec 2017	6	124	4.6%	0.25
Participant’s Preferred Method of Communication	*n* = 449			
Email	66	340	16.3%	0.89
Phone	7	25	21.9%	1.20
Phone, email	11	0	100.0%	5.49
Participant’s Relationship to Patient	*n* = 811			
Patient	150	380	28.3%	1.55
Family	9	209	4.1%	0.23
Friends	2	61	3.2%	0.17

^a^Overall average 18.2%

**Fig. 2:**
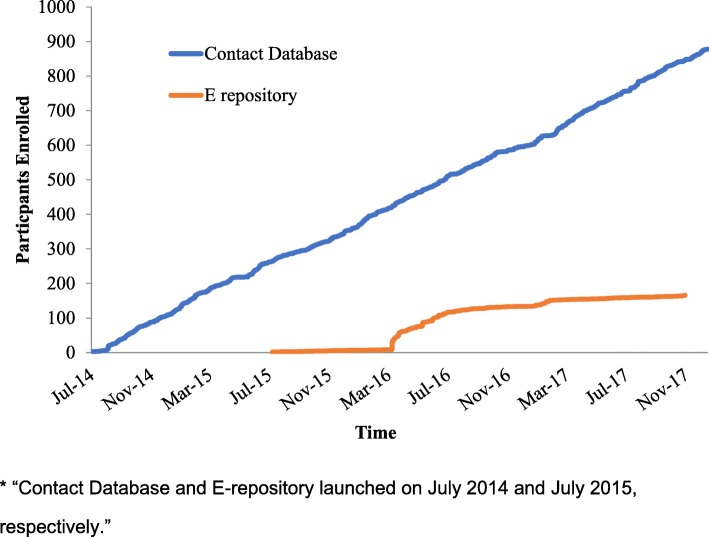
Graph of participant enrollment in the Contact Database and E-repository from July 2014 until December 2017. * “Contact Database and E-repository launched on July 2014 and July 2015, respectively”

We hypothesized that there would be a statistically significant rise in enrollment in the Contact Database and E-repository following the 2017 Patient Symposium, but there was no significant change. Figure 2 illustrates patient enrollments over time in both the Contact Database and the E-repository through December 2017. The Contact Database followed a steady and linear enrollment trajectory from its opening, averaging about 21 participants a month. For the E-repository, few individuals initially signed up (8 individuals in the first 8 months). Upon execution of a comprehensive enrollment strategy in March 2016 (see Additional file 1: E), there was a large rise in enrollment. Thirty-four patients signed up within 1 week and 56 signed up within 1 month. Over the next several months, the rate of enrollment continued at an increased pace and by the end of 2016 there were 134 patients in the E-repository.

## Discussion

Herein we present a description of the largest database of CD patients worldwide, characteristics associated with patients interested in contributing data and biospecimens for research, and insights into successful data and biospecimen acquisition for rare diseases.

The characteristics of CD patients in the Contact Database share several commonalities and differences with the existing literature. The age (41.8 ± 16.0) of CD patients in the Contact Database is similar to what has been reported previously for CD patients by Munshi et al. (mean: 42.9–45.8, SD 17.2–17.6) [6]. For subtypes of CD, the data also aligns with prior published ages from Talat & Schulte (median: 30 and 52 for UCD and MCD respectively) [7] and iMCD patients from Liu et al. (median: 50, IQR 35–61) [4]. The gender distribution (49% female) is also similar to that published by Munshi et al. (64.1%) for CD and Liu et al. (42.2%) for iMCD. Review articles often state that UCD predominantly affects females while MCD affects more males [8, 9]. However, when broken down by gender, both UCD and MCD had more females than males. There did seem to be a larger female predominance in the UCD group as compared to the MCD group, in line with the UCD-female association canon.

The only study of the incidence of CD estimated that MCD cases made up 1569–1756 (20–27%) of the 6502–7696 annual CD diagnoses in the USA [6]. Our results (41% MCD, 33% UCD, 26% unknown) suggest that the proportion of CD cases that have MCD may be higher than previous estimates. However, the Contact Database is highly susceptible to ascertainment bias and likely also does not represent the population distribution, as individuals choose to join the Contact Database for a variety of reasons. Factors such as personal motivation, access to the internet, technical savviness, and ability to navigate an English-language website may have contributed. Further, after surgical excision and treatment of their disease, UCD patients may proceed with their healthy lives and not be as interested in volunteering in research studies for their already treated disease as patients with MCD. The only prior estimate of the proportion of HHV8-negative iMCD relative to HHV8 + MCD was 34–55% vs 45–66%, respectively, based on the proportion in published case reports [4]. Our results (57–90% iMCD vs 10–43% HHV8 + MCD) suggest that the proportion of iMCD to HHV8 + MCD may be higher than previously thought.. However, patients with iMCD, which is not as clearly understood or well controlled as HHV8 + MCD, may be more likely to seek out support and answers through a rare disease organization as well as more likely to express interest in donating biospecimens for research. Likewise, the high proportion of patients enrolled from the US almost certainly represents the fact that the CDCN is headquartered in the US and media profiles have primarily occurred within the US.

The high proportion of respondents unaware of whether they had UCD or MCD is quite concerning as the clinical presentation, treatment, and survival outlook is significantly different. Approximately 35% of iMCD patients die within 5 years of diagnosis and another 25% die within 10 years of diagnosis [10]. Conversely, less than 5% of UCD patients die within 5 years of diagnosis and less than 10% of HHV8 + MCD patients die if appropriately treated with B cell depletion therapy. It is imperative that all physicians perform necessary testing to determine if the patient has UCD, HHV8 + MCD, or iMCD and that patients are better informed of their subtype. A possible explanation for this high proportion of patients unaware of their subtype may be that some individuals who register in the Contact Database don’t actually have CD or the subtype testing may not have been completed when the patient registered. A clear limitation of this study is that the patients’ diagnoses are patient reported and not validated with medical records. There may be discrepancies between what participants complete in the form and the medical record. The CDCN is currently co-leading enrollment into the ACCELERATE Natural History Registry, where patients can enroll themselves online, complete medical surveys, and grant access to medical records for in-depth data abstraction. By coupling self-reported surveys with medical record data, we should be able to gain insights into the reliability of self-reported surveys like the Contact Database and E-repository. The recent publication of the first-ever diagnostic criteria for iMCD may also help with determining and communicating this challenging diagnosis appropriately [11].

The analysis of differences between patients who only enroll in the Contact Database versus those who go on to enroll in the E-repository suggests that certain characteristics are associated with interest in tissue sample donation, such as female gender and MCD subtype. Female gender has previously been shown to be associated with more altruistic behavior such as filling out more surveys and donating money to nonprofits [12, 13]. Diagnosis with the more severe MCD subtype compared to UCD or unknown subtype of CD was also associated with significantly increased rates of E-repository enrollment. Some of the respondents with unknown subtype of CD may not be confident they actually have CD and so therefore may be less interested to contribute samples to its cause. Another explanation is that those patients who have a known primary diagnosis communicated to them are more motivated to participate in investigative efforts. The significant differences between the characteristics of patients in the Contact Database and the E-repository, including female gender, proximity to research/USA location, and more severe subtype may also be associated with tissue sample donation interest for other rare diseases too.

Additional insights were generated related to patient interest in contributing samples for research. Participants that A) signed up within the first 2 years of the Contact Database opening, B) preferred to be contacted both by email or phone (rather than one or neither), or C) were the patient (rather than a family member) were all significantly more likely to enroll in the E-repository. Regarding A, it seems that there was a first mover effect. Those individuals who were quick to sign up in the Contact Database were also more willing to express interest in donating tissue; these individuals were driven by the desire to be as involved as much as possible as quickly as possible. Similarly, even though a small number of individuals elected to be reached out to by both phone and email (*n* = 11), all eleven individuals signed up for the E-repository. As would be expected, individuals open to any form of contact regardless of the modality likely have a stronger desire to participate in research efforts.

Three characteristics that trended towards significance are also worth discussing. American participants trended towards being more likely to sign up for the E-repository. This likely has to do with the CDCN’s location in the USA and the proximity to most CDCN research studies. Individuals who expressed interest in a CDCN program geared at empowering patients and loved ones to spread awareness and raise funds for research also tended to be more likely to enroll in the E-repository. We suspect similar underlying motivations in these patients as those outlined for individuals who answered phone and email for their preferred communication. Finally, individuals who had carried a CD diagnosis for a longer period of time also trended towards being more likely to express interest in donating tissue. This may be secondary to these individuals experiencing a prolonged course with little scientific progress, which may have served as inspiration to get more involved with research. This may also reflect that patients further out from diagnosis are able to consider contributing to research more so than newly diagnosed patients, who are focused on finding an expert and getting care right away.

There are also several operational learnings from implementing and rolling out the Contact Database and E-repository, which may be helpful for other rare disease organizations. First, an electronic consent form has been shown to be effective for increasing enrollment into tissue repositories for a number of disease-specific and non-disease specific biobanks [14]. The IRB-approved electronic consent forms that the CDCN developed are included as supplementary files with this publication so other rare disease organizations may model their own consents after it in hopes of expediting other rare disease investigative efforts (Additional file 1: D). Second, visualizing the E-repository enrollment (Fig. 2) highlights a sharp peak around March 2016. A review of the CDCN’s internal records helps to explain the spike in enrollment. A CDCN task force developed a tissue acquisition plan in February 2016 called “The Answers are Within.” Only 8 patients had been enrolled in the E-repository in the prior 8 months. The strategy included posting an article to the homepage on CDCN.org, posting on social media, and sending tailored emails (Additional file 1: E) to members of the Contact Database. There were different pathways of communication for patients who reported having UCD, MCD, or being unsure. The outcome was a 15-fold increase (8 vs. 125 patients) in enrollment when comparing the 8 months pre vs post roll out of the strategy. Finally, considering the low enrollment of patients in Asian and African countries, moving forward, a greater emphasis should be made to partner with physicians in these regions and to adapt English language consents to local languages to encourage more patient enrollments in these regions. As larger numbers are enrolled into these databases within a given country, analyses should be conducted to determine regional differences in prevalence and demographics at the national level.

Despite several important limitations, the results presented come from the largest database of CD patients. The characterization of patients in the Contact Database and comparison with patients in the E-repository identified statistically significant differences that may assist with future rare disease biospecimen procurement. Commentary on managing these databases and sample documentation (e.g., electronic consent, recruitment materials) provided with this article should also assist future rare disease research efforts with adoption and implementation of biorepositories.

## Conclusions

This study of the largest- dataset of CD patients worldwide provides insights into disease phenotypes, characteristics of patients interested in contributing data and biospecimens for research, and methods for successfully acquiring data and biospecimens. Generally, the factors associated with enrollment in the E-repository represented severity of disease subtype, proximity to the research, and patient motivation. We hope that these findings and the sample documentation (e.g., electronic consent, recruitment materials) provided with this article will assist future rare disease efforts with overcoming hurdles.

## Additional file

Additional file 1: Examples of webpage screenshots, email communication, tissue donation forms, electronic consents, and E-repository enrollment documents. (PDF 1553 kb)

## Data Availability

The data that support the findings of this study are available from the authors upon reasonable request and with permission of Quorum IRB. Much of the data infrastructure has been made available in our Additional file 1.
