# Supplementary material for: Quantitative analysis of a rare disease network’s international contact database and E-repository provides insights into biobanking in the electronic consent era
Source: Orphanet J Rare Dis. 2019 Jul 11;14:173. doi: 10.1186/s13023-019-1145-y (PMC6625003; doi:10.1186/s13023-019-1145-y)
Supplement: Supplementary file 1 — Examples of webpage screenshots, email communication, tissue donation forms, electronic consents, and E-repository enrollment documents. (PDF 1553 kb) [file 13023_2019_1145_MOESM1_ESM.pdf]

Supplementary Materials

A – Screenshots of the CDCN webpages for participants to sign up for the Contact Database and E-repository

B – Email communication following Contact Database registration

C – Tissue Sample Donation Interest Form

D – Copy of Electronic Consent documents for CDCN Biobank

E – E-repository Enrollment Strategy Documents

A

Screenshots of the CDCN webpages for participants to sign up for the Contact Database and E-repository

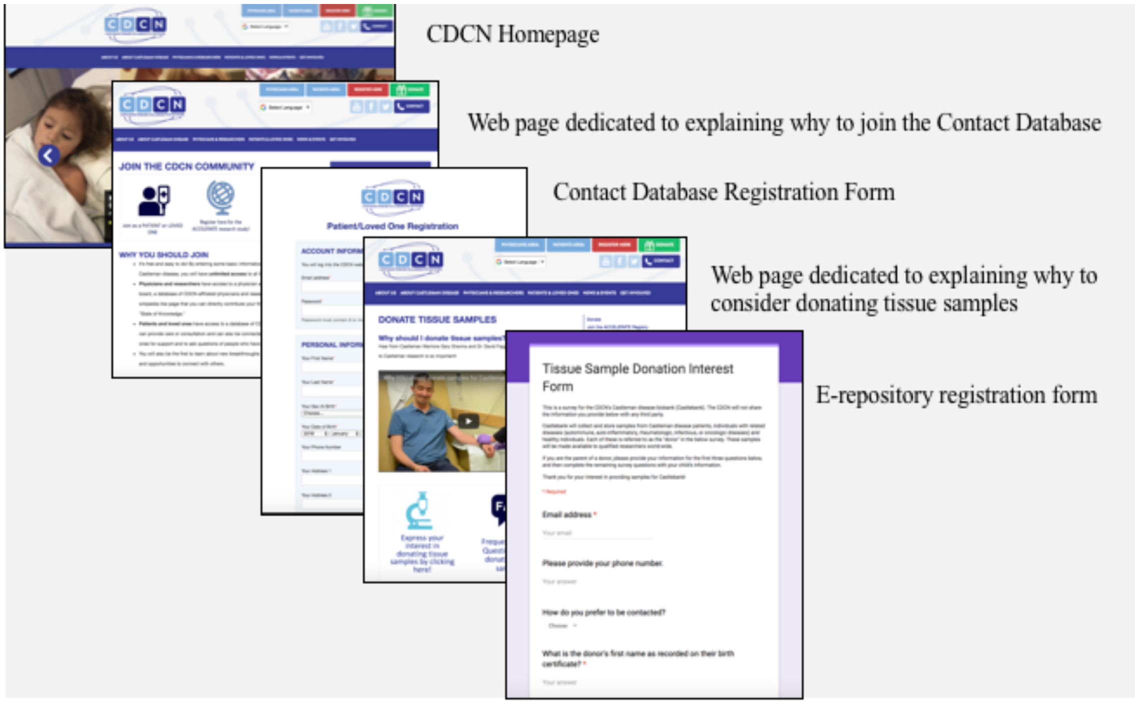

B

1
Email #1: Automated email after joining Castleman Network

Title: Thank you for joining Castleman Network!

Welcome to the Castleman Disease Collaborative Network!

The CDCN has made incredible progress over the last 4 years as a result of hundreds of patients, loved ones, physicians and researchers working together through the network. We have more work ahead of us to identify the cause(s) of Castleman disease, which immune cells are involved and which treatments are effective so we can ultimately cure this disease. Learn more about our progress by visiting http://www.cdcn.org/about-us/impact and watching our new video: https://youtu.be/9PcJscr5xT8!

There are three important ways that you can get involved in our effort:

1. Please consider donating precious samples to the CDCN BioBank to ensure research continues. Learn more about contributing samples for research at http://www.cdcn.org/get-involved/donate-tissue and express your interest in contributing samples by filling out this brief survey: https://goo.gl/KuZRPS. The answers are within each of us!
2. The ACCELERATE Natural History Registry Study is now enrolling! We invite all patients, parents of pediatric patients, children of adult patients, family members and loved ones of deceased patients to enroll. By gathering medical data from patients and their families, we aim to unlock the history of Castleman disease, its treatment and outcomes. For more information or to enroll, please visit: http://www.cdcn.org/accelerate.
3. All of our research efforts will not be possible without funding. Learn how you can get involved and make an impact as a Castleman Warrior: http://www.cdcn.org/get-involved.

If you are interested in joining the fight against Castleman Disease in any/all of the above ways, please feel free to reach out at any time.

Sincerely,

[CDCN Member's Name]
[CDCN Member's Name]'s email signature
Connect with us on Facebook: https://www.facebook.com/cdcn2/

Email #2: Automated email after joining Castleman Network

Title: Thank you for joining Castleman Network!

Dear *PATIENT FIRST NAME*,

Thank you for joining the Castleman Disease Collaborative Network (CDCN) as a **patient*. I am sorry to hear about your diagnosis with Castleman disease. I hope that we at the CDCN will be able to offer you support moving forward. I want to address each of the areas you requested help in, so I apologize in advance for the long email!

Understanding Castleman disease:
Castleman Disease describes a rare group of three separate inflammatory disorders that share similar lymph node appearance under the microscope. Unicentric Castleman disease (UCD) is diagnosed in patients with enlarged lymph nodes in one region of the body. Patients with multicentric Castleman disease (MCD) have enlarged lymph nodes in more than one area of the body. There are two subtypes of MCD: one that is caused by a virus (HHV-8-positive MCD) and another whose cause is unknown (HHV-8-negative or idiopathic MCD). When diagnosed with MCD, it is important that the patient's lymph node is tested for HHV-8 virus as the two types require different treatments.
IF UCD include This would not apply to you since based on your submission form you were diagnosed with UCD.
IF TAFRO symptoms include Based on your submission form, you may have a particular subtype of idiopathic MCD known as TAFRO syndrome. TAFRO stands for thrombocytopenia (low platelets), ascites (fluid accumulation), microcytic anemia, myelofibrosis (bone marrow fibrosis), renal dysfunction (kidney dysfunction), and organomegaly (enlarged liver or spleen).

There is a great deal of information about Castleman disease available here: http://www.cdcn.org/about-castleman-disease
Our 2017 Community Update is also a good resource for understanding this disease: http://files.www.cdcn.org/CDCN_annual_report_170403_-_final.pdf

Treatment options:
Treatment depends on the subtype of disease that you are diagnosed with. First-line treatment of UCD is complete removal of the swollen lymph nodes, including any surrounding lymph nodes if they may be enlarged. If complete removal of the swollen lymph nodes is not possible, some UCD patients require treatments used for idiopathic MCD. There are no established treatment guidelines that have been published for idiopathic MCD. I am happy to share more specifics about treatment options once we determine the subtype, but in the meantime, please take a moment to check out our new Therapeutic Target Dashboard (http://www.cdcn.org/about-castleman-disease/treatments), which describes treatments that have been used for UCD, idiopathic MCD and HHV-8-positive MCD.

Finding a Doctor:

I have attached our Physician Referral List to this email. I would like to call special attention to Dr. van Rhee, who has a great deal of experience treating Castleman disease patients.
IF INTERNATIONAL remove van Rhee

Peer Support:
When diagnosed with a rare disease like Castleman disease, it can help for patients and their loved ones to connect with others experiencing similar struggles.

1. Many patients connect on Facebook through the CDCN Facebook group (https://www.facebook.com/cdcn2/) and the International Castleman Disease Organization group (https://www.facebook.com/groups/48343887930/).

2. We also have an Ambassador team, which is a group of patients and loved ones that connect with new members of our community. Would you be interested in being connected with another patient? If yes, I will pass along your email so they can reach out.

3. Thank you for expressing interest in joining the Castleman Warriors! The Warriors are a group of patients and loved ones that connect monthly. They work together to raise awareness and fundraise for Castleman disease research in their community. You can sign up here: http://www.cdcn.org/get-involved/castleman-warriors

Donating Samples and Joining Research Studies:
The best way to get involved in high-impact research for Castleman disease is to enroll in ACCELERATE and Castlebank.

1. ACCELERATE Patient Natural History Study is a study that collects medical records and prior lymph node biopsy samples of Castleman disease patients around the world. Through collecting medical records, we hope to learn more about and find treatments for this rare disease. You can learn more about ACCELERATE or sign up for this study here: http://cdcn.org/accelerate

2. If you would like to contribute blood samples for research, please visit: http://www.cdcn.org/samplesOnce you fill out this form, we will contact you with instructions so you can contribute your sample to Castleman disease research!

3. Have questions or ideas about Castleman disease and what we should be focusing our research on? Fill out the Google form here: http://www.cdcn.org/patients-loved-ones/patient-driven-research-opportunities to be added to our patient driven research initiative.

Please let me know if you have any additional questions and do stay in touch about **PATIENT'S** health.

All my best,
[CDCN Member’s Name]

Usually Physician Referral List sent as attachment

C

Tissue Sample Donation Interest Form

This is a survey for the CDCN's Castleman disease biobank (Castlebank). The CDCN will not share the information you provide below with any third party.

Castlebank will collect and store samples from Castleman disease patients, individuals with related diseases (autoimmune, auto-inflammatory, rheumatologic, infectious, or oncologic diseases) and healthy individuals. Each of these is referred to as the "donor" in the below survey. These samples will be made available to qualified researchers world-wide.

If you are the parent of a donor, please provide your information for the first three questions below, and then complete the remaining survey questions with your child's information.

Thank you for your interest in providing samples for Castlebank!

* Required

Email address *

Please provide your phone number.

How do you prefer to be contacted?

What is the donor's first name as recorded on their birth certificate? *

What is the donor's middle name as recorded on their birth certificate? *

What is the donor's last name as recorded on their birth certificate? *

What is the donor's sex as recorded on their birth certificate? *

Female
Male

What is the donor's date of birth as recorded on their birth certificate? *

In which city, town, or village was the donor born as recorded on their birth certificate? *

In which country was the donor born as recorded on their birth certificate? *

Donor's ethnicity (please select all that apply). *

American Indian or Alaska Native
Asian
Ashkenazi Jewish
Black or African American
Hispanic/Latino
Native Hawaiian or other Pacific Islander
White
Unknown
No response

What is the donor's diagnosis? *
Castleman Disease
Related Disease
Health Donor

[If person answers,…
“Castleman Disease, they get directed to the page Titled “Diagnosis Castleman Disease”
“Related Disease, they get directed to the page Titled “Diagnosis Related Disease”
“Healthy Donor, they get directed to the page Titled “Healthy Donor”]

Tissue Sample Donation Interest Form

Diagnosis: Castleman disease

What was the date when the donor first experienced Castleman disease symptoms?

What was the date when the formal Castleman disease diagnosis was made?

What is the donor's subtype of Castleman disease?

Unicentric Castleman disease

HHV-8 positive multicentric Castleman disease

HHV-8 negative (idiopathic) multicentric Castleman disease

I have multicentric Castleman disease, but I am unsure if I am HHV-8 positive or negative

I do not know

Which of the below best describes the donor's Castleman disease course?

The donor has experienced at least two periods or Castleman disease flare-ups (symptoms believed to be related to Castleman disease) that are separated by a period of remission

The donor's Castleman disease symptoms have never fully resolved and s/he has experienced constant symptoms throughout

The donor has experienced at least one point of Castleman disease flare-up (symptoms they believe to be related to Castleman disease) and is now in remission

I don't know

Has the donor previously or are they currently experiencing any of the below symptoms? Please select all that apply.

Swollen lymph nodes

Swelling or edema in other body area

Fatigue

Night sweats

Fever

Unintentional weight loss

Rash or skin lesions

Enlarged liver

Enlarged spleen

Please list any other symptoms related to their Castleman disease that the donor is currently experiencing?

Please list all medications that the donor is currently receiving to treat their Castleman disease.

In the last two years, has the donor received rituximab/rituxan (anti-CD20), chemotherapy, or other immune-depleting therapy (therapies that 'kill' the immune system)?

Yes

No

If so, please provide the therapy and the month and year it was received.

Has the donor felt unwell in the last two weeks (e.g. experienced a cold, flu, or other illness)?

Yes

No

If so, please provide details.

Tissue Sample Donation Interest Form

Diagnosis: Related disease

What is the donor's autoimmune, auto-inflammatory, rheumatologic, infectious, or oncologic disease diagnosis?

What was the date when the formal diagnosis was made?

Has the donor ever tested positive for Kaposi's sarcoma-associated/human herpes virus-8 (HHV-8) or human immunodeficiency virus?

Yes

No

Please list all medications that the donor is currently receiving.

Has the donor felt unwell in the last two weeks (e.g. experienced a cold, flu, or other illness)?

Yes

No

If yes, please provide the details.

Please list any other health information for the donor that might be important.

Tissue Sample Donation Interest Form

Diagnosis: Healthy donor

Has the donor ever been diagnosed with an autoimmune, auto-inflammatory, rheumatologic, infectious, or oncologic disease diagnosis?

Yes

No

If yes, please provide details.

Please list all medications that the donor is currently receiving.

Has the donor felt unwell in the last two weeks (e.g. experienced a cold, flu, or other illness)?

Yes

No

If yes, please provide details.

Please list any other health information for the donor that might be important.

Tissue Sample Donation Interest Form

Thank you for your interest in donating tissue samples!

D

Parent of pediatric CD patient < 7

Thank you for filling out this Survey, so that we will be able to include your samples in the BioBank.

If you are presented with a Survey Page that does not contain any questions, please select "Next Page" to continue through the Survey. That particular Survey Page does not contain any questions that pertain to you based on your prior responses.

If you have any questions, please contact Dustin Shilling at 1-215-614-0689 or dustin@castlemannetwork.org

The following form will enable you to participate in the CDCN Biobank (Castlebank). Tissue samples such as blood or excess biopsy samples from patients will enable scientists to better characterize and understand Castleman disease and develop better treatments. Any blood or tissue donation will be used for high-impact research to battle this disease!

Is this your first time filling out this Survey? (If you do not remember if you have participated in the past, please call 1-215-614-0689)

X First Time Participant

_ Repeat Participant

--NEXT PAGE--

Please select the appropriate category that describes the PERSON completing this survey. (This question refers to the person completing the survey, NOT necessarily the patient)

_ Adult CD patient (> 18 years old)

X Parent of pediatric CD patient

_ Related family member of CD patient (without CD and > 18 and NOT parent of pediatric CD patient)

_ Patient with related disease (e.g. Lupus, Rheumatoid Arthritis and > 18 years old)

_ Healthy volunteer (> 18 years old)

Please select the appropriate age band for the patient. (Please let us know the age band of the patient. This is needed to determine the consent requirements.)

_ Patient > = 14 or < 18

_ Patient > = 7 or < 14

QUORUM REVIEW
APPROVED
INSTITUTIONAL
REVIEW BOARD

X Patient < 7

RESEARCH CONSENT/ASSENT FORM – KEY POINTS

For Adult Participants or Parents/Guardians

For Minors Age 14 and Older

For Participants Who Become Adults While in the Study

Study Title: The Castleman Disease Collaborative Network Biobank: A Collection of Biospecimens and Clinical Data to Facilitate Research
Study #: 001
Sponsor: Castleman Disease Collaborative Network
Study Investigator: David Fajgenbaum
Castleman Disease Collaborative Network
1001 S. 18th St., Unit A., Philadelphia, PA 19146
Telephone Number: (610) 304-0696

Potential Participants 18 years and older: This is a consent form. It provides a summary of the information the research team will discuss with you. If you decide that you would like to take part in this research study, you would sign this form to confirm your decision.

Potential Teen Participants: This form also serves as an assent form. That means that if you choose to take part in this research study, you would sign this form to confirm your choice. Your parent or guardian would also need to give their permission and sign this form for you to join the study.

Parents/Guardians: You have the option of having your child or teen join a research study. This is a parental permission form. It provides a summary of the information the research team will discuss with you. If you decide that your child can take part in this study, you would sign this form to confirm your decision.

When reading this form, please note that the words “you” and “your” refer to the person in the project rather than to a parent or guardian who might sign this form on behalf of the person in the project.

Participants who become adults during the study: Because you are now an adult, you have been asked to read this consent form. After you have read the form, you will be asked to sign it if you still agree to have your samples and information stored and used in a variety of research studies.

The goal of the “Castleman Disease Collaborative Network Biobank” project is to be a patient-driven movement that empowers people with Castleman Disease to directly transform research and treatment of disease by providing blood, sharing part of their stored tissue, and copies of their medical records for

QUORUM REVIEW
APPROVED
INSTITUTIONAL
REVIEW BOARD

use in a variety of research projects. Because we are enrolling people with Castleman across the country regardless of where they are being treated, this study will allow many more people to contribute to research than has previously been possible.

1. What is the purpose of this study?

We want to understand Castleman Disease better so that we can develop more effective therapies. The Castleman Disease Collaborative Network would like to partner directly with people with Castleman Disease, so we can study many more aspects of Castleman Disease than would otherwise be possible.

2. What will I have to do if I agree to participate in this study?

You will answer some questions about your disease and medical care and send a blood sample and/or a saliva sample to us in a pre-stamped package that we will provide. The blood sample should be collected during your regularly scheduled clinical visit if possible. We will take care of obtaining copies of your medical records as well as small amounts of your stored tissues (if available) from the hospitals or centers where you receive your medical care.

3. Do I have to participate in this study?

No. Taking part in this study is voluntary. Even if you decide to participate, you can always change your mind and leave the study.

4. Will I benefit from participating?

While taking part in this study will not improve your own health, the information we collect will aid in research efforts to provide better treatment and prevention options to people with Castleman in the future. We will provide updates about key research discoveries made possible by this biobank on our website, and directly to you via email if you so choose.

5. What are the risks of taking part in this research?

There may be a risk that your information (which includes your genetic information and information from your medical records) could be seen by unauthorized individuals. However, we have procedures and security measures in place designed to minimize this risk and protect the confidentiality of your information.

Investigators do not plan on returning the research test results to you, and the results will not be placed in your regular medical record. However, if a research test reveals something that could potentially affect your care (for example, a possible misdiagnosis), they may contact your physician to have the tests confirmed in a clinical lab. Your physician, not the investigator, will then follow-up with you. We

QUORUM REVIEW
APPROVED
INSTITUTIONAL
REVIEW BOARD

will obtain consent from you to provide your physician your research results and ask you for updated physician contact information at that time.

If you decide to give blood for this biobank, you may experience pain, bruising, dizziness, or an infection.

6. Will it cost me anything to participate in this study?

No.

7. Who will use my samples and see my information?

Your samples and health information will be available to researchers at the Castleman Disease Collaborative Network (CDCN), a not-for-profit global initiative dedicated to accelerating research and treatment for Castleman disease. Your samples may also be used by outside researchers, both in the U.S. and internationally. After removing your name and other readily identifiable information, we will share tissues and information with qualified researchers, who have undergone review and approval by a CDCN Advisory Board. Results from the research studies may be shared with the greater research community via publications, as well as central data banks at the National Institutes of Health.

8. Can I stop taking part in this research study?

Yes, you can withdraw from this research study at any time. CDCN will destroy all your samples in its possession; if samples and information have already been sent to researchers, the CDCN will notify the researcher of the participant's wishes but the CDCN cannot guarantee these samples will be destroyed. In addition, research data may have already been published before you withdrew and that cannot be retracted. Your information would be removed from future studies.

9. What if I have questions?

If you have any questions, please contact either the Principal Investigator, Dr. David Fajgenbaum at (215) 614-0936. In case you want to inquire via writing, their address is 1001 S. 18th St, A Philadelphia PA 19146.

FULL RESEARCH CONSENT FORM

Adult Participants or Parents/Guardians

The Castleman Disease Collaborative Network Biobank: A Collection of Biospecimens and Clinical Data to Facilitate Research

QUORUM REVIEW
APPROVED
INSTITUTIONAL
REVIEW BOARD

A. Introduction

You are being invited to participate in a research study that will establish a Castleman Disease biobank. A biobank, sometimes called a biorepository, collects, stores, analyzes and distributes samples of human tissues, blood, and related health information to qualified scientists. Creation of a biobank for this disease may help doctors and researchers better understand why Castleman Disease occurs and develop ways to better treat and prevent it. Because Castleman Disease is so rare, it has not been well studied, due in part to a lack of available biospecimens and data for research use.

You are being asked to participate in the study because you have Castleman Disease. With your decision to participate, the CDCN will have access to your already existing tissues (blood, bone marrow, and/or lymph node) from your hospital. You also have the ability to provide additional blood or saliva samples.

The biobank is being established by the Castleman Disease Collaborative Network (CDCN), a global initiative dedicated to accelerating research and treatment for Castleman Disease. We work to achieve this by facilitating collaboration among the global research community, mobilizing resources, strategically investing in high-impact research, and supporting people with Castleman Disease and their family members. This biobank is paid for by the CDCN.

This form describes the project in order to help you decide if you want to participate. This form will tell you what you will have to do and the risks and benefits of participating.

If you have any questions about or do not understand something in this form, you should ask the research staff. You should also discuss your participation with anyone you choose in order to better understand this project and your options. Do not sign this form unless the staff has answered your questions and you decide that you want to be part of this project.

Participating in research is not the same as getting regular medical care. The purpose of regular medical care is to improve your health. The purpose of medical research is to advance the understanding and treatment of disease. Participating in this project does not replace your regular medical care.

B. What is the purpose of this biobank?

The Castleman Disease Collaborative Network is creating this biobank to support research. We want scientists to better understand Castleman Disease and work toward developing more effective therapies. We would like to partner directly with people with Castleman, so we can study many more aspects of Castleman than has previously been possible. In addition, because we are enrolling people with Castleman across the country regardless of where they are being treated, this study will allow many more people to directly contribute to research than might otherwise be feasible.

By participating in this biobank project, you will be asked to provide blood or saliva now or in the future, tissue (lymph node, bone marrow samples) left over from past medical tests, and/or medical information to be used for research. We would also like your permission to access and share (after removing your name and other personal identifiers) your medical information along with your specimens.

QUORUM REVIEW
APPROVED
INSTITUTIONAL
REVIEW BOARD

Some of the research might involve studying DNA contained in your samples. DNA is the material that makes up genes. Genes determine things like hair color and eye color, and may play a role in Castleman Disease. Using DNA from your blood or tissue sample, researchers will study your entire genetic sequence, known as your genome, and store this information. Your genomic data and health information will be studied along with information from other participants in this study, and it will be stored for future studies.

Some of the research might involve creating and distributing cell lines from your tissue so that your cells can grow indefinitely to be used in various projects. Cell lines may be useful because of the characteristics of the cells and/or the products they may produce.

Samples may be studied immediately or may be stored for future use by researchers from universities, the government, health-related companies, and other research institutes. Some researchers will be from the United States and some may be from other countries around the world. A Biobank Advisory Board (BAB) composed of physicians and scientists will review research requests to help ensure that tissues are being utilized in the most impactful manner to aid our understanding of Castleman Disease. The BAB will not to attempt to re-identify you. Future researchers must also sign an agreement they will not attempt to re-identify you. Because technology is changing all the time it is not possible to know all of the techniques that researchers may use now or in the future.

We are asking many people to participate in the biobank, both people with Castleman, their family members, and healthy individuals (as a comparison group). We don't know how many people will participate.

C. What other options are there?

Your participation in the biobank project is voluntary. Your alternative is not to participate. The biobank project does not involve treatment for any condition, so your decision to participate or not to participate will not affect your medical care. Your decision not to participate or to stop participating at any time will not involve any penalty to you and will not involve any loss of benefits to which you might otherwise be entitled.

D. What is involved in the biobank project?

With your consent, project staff will collect medical information through a secure questionnaire regarding your health, the doctors you have seen, as well as your medical and family history. Alternatively, a staff member where you receive your care may review your medical records to collect such information. The medical information may include your patient history, response to treatment, pathology (disease) reports, and radiographic (imaging) reports.

The types of samples the Castleman Disease Collaborative Network wants to collect include:

- leftover tissue (such as lymph nodes and/or bone marrow) that have been taken, are scheduled to be taken, or that may be taken in the future, as part of your regular medical care
- blood samples already stored, scheduled to be taken, or that may be taken in the future

QUORUM REVIEW
APPROVED
INSTITUTIONAL
REVIEW BOARD

- saliva

Each type of sample collection is optional. You can decide to participate in each type of sample collection separately, which means you can agree to some, all, or none of the different types of samples. You can indicate your choice at the end of this form.

If you have had surgery in the past and you provide consent, project staff will contact the hospital(s) where you had surgery to get leftover tissue or fluids that may be stored there. If you are scheduled for a medical procedure or surgery for your care, no additional tissue will be taken beyond what is needed for your care. After the medical staff has used your tissue for diagnosis and care, any leftover samples you have agreed to provide will be transferred to the biobank.

If you consent to have blood drawn for the biobank, it may be collected at the same time as other blood tests or during your surgery, if possible. The biobank will send you blood collection kits with tubes and instructions that you can take to your doctor or local laboratory so they can obtain one or more blood samples. The amount of blood collected will be no greater than 50 mL (about 3 tablespoons) over a 2-month period.

We will link the results of the tissue collected with medical information that has been generated during the course of your regular treatment. We are asking your permission to obtain a copy of your medical record from places where you have received care for your disease.

If you give your consent to be re-contacted, biobank staff may contact you periodically to update your medical information or to ask you to provide new samples for research. You may also choose if you would like the Castleman Disease Collaborative Network to notify you about other research studies in the future.

People who work with the sponsor (CDCN) will store your samples in a secure laboratory. Your samples will be stored as long as the sponsor decides to keep them.

E. How long will I be in this research study?

Your direct involvement will be limited initially to the time it takes to obtain your consent and to collect samples and medical information. Because this project involves a biobank, your samples, data, and health information may be used immediately or may be stored indefinitely for future use. If you agree, we may also re-contact you to get updates about your health and medical care, to ask whether you will give additional samples, or to tell you about other studies.

If a participant is a minor when his/her samples are first placed in the biobank, and he/she later becomes an adult, the project staff will contact the participant to seek his/her consent to continue to use the samples in the biobank. At that time, the participant can decide to continue in this biobank project, or ask that the samples be destroyed. If such a participant cannot be contacted, any samples still in the bank will be retained and will continue to be distributed in de-identified form.

If you want to stop being in the biobank project, contact the Principal Investigator or project staff using the contact information provided later in this form. If you decide to withdraw from the project early, the Principal Investigator or project staff may ask you some questions about being in the project to gain

QUORUM REVIEW
APPROVED
INSTITUTIONAL
REVIEW BOARD

feedback on ways in which biobank may be improved. The Principal Investigator or the sponsor can remove you from the project at any time, even if you want to stay in the project.

F. What kind of information could be found in this study and will I be able to see it?

This research is being done to add to our knowledge of Castleman Disease, and is not intended to provide information about your individual health or treatment. Investigators do not plan on returning the research test results to you, and the results will not be placed in your regular medical record. However, if a research test reveals something that could potentially affect your care (such as a possible misdiagnosis) they may contact your physician to have the tests confirmed in a clinical lab. Your physician, not the investigator, will then follow-up with you.

If you choose, the Castleman Disease Collaborative Network can also keep you up to date on the research outcomes of projects using samples or data from this biobank; however not all research projects will use samples from all the donors to the bank, so your own tissue may or may not have been part of a particular research project. You do not need to participate in this biobank to be kept up-to-date on the Castleman Disease Collaborative Network's research efforts; instead, you can sign up online for our email list here at cdcn.org. We regularly update the website.

If you choose, your samples and information may also be used for other central databanks. To allow research collaborations and sharing of data, the National Institutes of Health (NIH) and other research organizations have developed special data (information) repositories that analyze data and collect the results of certain types of genetic studies. These central banks will store your genetic information and provide them to qualified researchers to do more studies. This information will be available for any research question, such as research to understand what causes certain diseases (for example heart disease, cancer, or psychiatric disorders), development of new scientific methods, or the study of where different groups of people may have come from. We are also asking your permission to share your results with these special controlled-access banks. Controlled-access means that only researchers who apply for and get permission to use the information for a specific research project will be able to access the information. Your genomic data and health information will not be labeled with your name or other information that could be used to identify you. Researchers approved to access information in the database will agree not to attempt to identify you. Your information will be sent only with a code number attached. All directly identifiable information, such as your name, will not be shared with data banks or other investigators. Although there may be a small risk of loss of privacy when sharing this information with these banks, we have established procedures to encode your samples and information and protect your data. Although we will do everything we can to protect the privacy of your data, we cannot absolutely guarantee its privacy or predict how genetic information will be used in the future.

We will publish important discoveries found through these studies in the scientific literature so that the entire research community can work together to better understand Castleman Disease. Your individual data will not be published in a way in which you could be readily identified. Abstracts, which are plain language summaries of the published reports, will be available to you and the general public.

G. What are the risks or discomforts of the biobank project?

QUORUM REVIEW
APPROVED
INSTITUTIONAL
REVIEW BOARD

Your regular medical care will not change if you participate in this biobank project. The collection of tissue samples would only take place in the context of your routine care, and therefore does not add any physical risk to you that you would not already be undertaking. If you agree to give an additional blood sample, the medical staff will take your blood by sticking a needle in your arm or they may collect the blood during surgery. The amount of blood that will be collected will not exceed 50 mL (about 3 tablespoons) over a 2-month period. Some problems you might have from this are:

- It may hurt.
- You may get a bruise.
- You may feel dizzy.
- You may get an infection.

There are some risks related to the use of your medical information and specimens. There could be an accidental release of your medical information to people who are not involved in your medical care. An accidental release of your genetic information could be used to identify you and your family members. We have tried to minimize this risk by carefully limiting access to the computers that would house your information and having other strict data security measures in place. There is still a risk someone could get access to the information we have stored about you, including information about a family member (for example, revealing that you or a blood relative carry a genetic disease). Because your DNA information is unique to you, there is a chance that someone could trace it back to you. The risk of this happening is small, but may be greater in the future.

Federal and State laws also provide some protections against discrimination based on genetic information. For example, the Genetic Information Nondiscrimination Act (GINA) makes it illegal for health insurance companies, group health plans, and most employers to discriminate against you based on your genetic information. However, it does not prevent companies that sell life insurance, disability insurance, or long-term care insurance from using genetic information as a reason to deny coverage or set premiums.

H. What are the benefits of the research study?

Research conducted with your information and specimens will not help you directly, but it may help people with Castleman in the future. Research performed with your information and specimens could result in the discovery and development of new drugs, tests or commercial products.

I. Can I stop being in the research study and what are my rights?

You can stop being in the research study at any time. You may choose to leave your samples and/or information in the biobank but request not to be re-contacted. Or, if you want to end all participation, the CDCN will destroy all of your sample identifiers and medical information in its possession; the CDCN will retain your original consent and the documentation of your withdrawal of consent. Any specimens or data that have already been distributed in a de-identified manner cannot be destroyed, and some research data may have already been published. If data has already been distributed to third parties, it

QUORUM REVIEW
APPROVED
INSTITUTIONAL
REVIEW BOARD

cannot be retrieved (as it will have been de-identified). Any specimens or identifiable data you have given can be removed upon request, but any de-identified, already distributed information or specimens cannot be withdrawn.

To withdraw from research, you must notify, in writing, the Principal Investigator (Address: 1001 S. 18th St, A Philadelphia PA 19146). There are three ways you can choose to stop participating in the Biobank:

1. You may choose to leave your data and unused samples in the Biobank while requesting not to be re-contacted in the future.

2. You may request that your personally identifying information be destroyed, but allow some or all of your samples to continue being used anonymously for future research.

3. You can choose to end all participation, in which case all of the unused samples in the Biobank will be destroyed and your medical information will be deleted.

J. Will I be paid to take part in this research study?

You will not get payment for being in this biobank project. The sponsor does not plan to give you any money or payment resulting from any discoveries or products made from this research project.

K. What are the costs?

Participating in the biobank project should not cost you anything beyond your routine, scheduled medical costs. If you decide to donate saliva and blood samples, we recommend you get these drawn during your regularly scheduled clinical appointments. You and your insurance company will already be paying for the costs of the blood draws as a part of your standard medical care. That way there's no additional cost. If you don't have regularly scheduled follow-up but still want to participate, please visit an outpatient clinic to get your blood drawn. You will need to pay for the cost of the blood draw but the CDCN can reimburse you if you fill out a W2 form and provide a copy of the original receipt. If you have a future scheduled biopsy planned, you and your insurance company will already be paying for the costs of that procedure, so there will be no additional cost. Neither you nor your insurance company will be asked to pay for research tests.

L. What happens if I am injured or sick because I took part in this research study?

If injury (physical, psychological, social, financial, or otherwise) occurs as a result of participation in this project, there are no plans for providing compensation to you or your family.

Be aware that your health care payer/insurer might not cover the costs of study-related injuries.

You do not give up any of your legal rights by signing this form.

M. What about confidentiality?

QUORUM REVIEW
APPROVED
INSTITUTIONAL
REVIEW BOARD

Your individual data and health information (without your name, address, or other details that might identify you) will be put in a controlled-access database. This means that only researchers who apply for and get permission to use the information for a specific research project will be able to access the information. Researchers approved to access information in the database will agree not to attempt to identify you. Access to information with your name, such as the consent form, will be restricted to healthcare staff, the Principal Investigator, designated CDCN staff, and approved CDCN contractors. Documents will be kept in a locked cabinet and information in computers will be protected by passwords. In order to protect your private information, only a unique code number, not your name or medical record number, will appear on your samples or on information sent to researchers outside the CDCN. Some people who work for the biobank will be able to find your name from the code so they can possibly have your sample(s) destroyed if you change your mind later. Except when required by law, your personally identifying information will not be disclosed outside of the CDCN.

N. Whom do I contact if I have questions about the research study?

If you have any questions about the study, please contact the research doctor or study staff listed below by email davidfajgenbaum@gmail.com or calling 215-573-8101:

- David Fajgenbaum, MD (Principal Investigator)

If you prefer to do so in writing, please send written correspondence to the address 1001 S. 18th St, A Philadelphia PA 19146.

You can learn more about the Castleman Disease Collaborative Network by visiting our website at www.cdcn.org.

Quorum Review Institutional Review Board (IRB) has reviewed this project. An IRB is a group of people who review research studies to protect the rights and welfare of research participants. If you have questions about your rights as a research participant, if you are not able to resolve your concerns with the Principal Investigator, if you have a complaint, or if you have general questions about what it means to be in a research project, you can call Quorum Review or visit the Quorum Review website at www.quorumreview.com.

Quorum Review is located in Seattle, Washington.

Office hours are 8:00 AM to 5:00 PM Pacific Time, Monday through Friday.

Ask to speak with a Research Participant Liaison at 888-776-9115 (toll free).

O. Conflict of Interest

Dr. David Fajgenbaum, the person responsible for the conduct of this research study also serves as the Executive Director of the CDCN. You should be aware that you are not under any obligation to participate in this research study.

QUORUM REVIEW
APPROVED
INSTITUTIONAL
REVIEW BOARD

P. Authorization to use your health information for research purposes

Because information about you and your health is personal and private, it generally cannot be used in this research study without your written authorization. Federal law called HIPAA requires that your health care providers and healthcare institutions (hospitals, clinics, doctor's offices) protect the privacy of information that identifies you and relates to your past, present, and future physical and mental health conditions.

If you agree to take part in this research, we will request that your health care providers and healthcare institutions disclose your protected health information to the Castleman Disease Collaborative Network for use in this research study. This may mean that you need to sign an additional form specifically authorizing release to the Network, but this will depend on your doctor's local practices, and we will work with you on this.

This section informs you about how your health information will be used or disclosed in the study. Your information will only be used in accordance with what is described here and as required or allowed by law. If you do not want to allow the uses described here, you cannot be in this biobank. You can still be in the biobank even if you do not give permission for the use and sharing of your information for the optional parts of the biobank.

1. What personal information about me will be used or shared with others during this research?

- Your medical records
- Your tissue samples relevant to this research study and related records
- New health information created from study-related tests and/or questionnaires

2. Why will protected information about me be used or shared with others?

The main reasons include the following:

- To conduct and oversee the research described earlier in this form;
- To ensure the research meets legal, institutional, and accreditation requirements;
- To conduct public health activities (including reporting of adverse events or situations where you or others may be at risk of harm)

3. Who will use or share protected health information about me?

With your consent, biobank staff will obtain information about you either directly from you or by having healthcare staff review your medical records. They may share your consent information in connection with the research study.

QUORUM REVIEW
APPROVED
INSTITUTIONAL
REVIEW BOARD

4. With whom outside of the Castleman Disease Collaborative Network may my personal health information be shared?

While all reasonable efforts will be made to protect the confidentiality of your protected health information, it may also be shared with the following entities:

- Federal and state agencies (for example, the Department of Health and Human Services, the Food and Drug Administration, the National Institutes of Health, and/or the Office for Human Research Protections), or other domestic or foreign government bodies if required by law and/or necessary for oversight purposes. A qualified representative of the FDA, the National Cancer Institute, or Quorum Review may review your medical records.
- Outside individuals or entities that have a need to access this information to perform functions relating to the conduct of this research such as data storage companies.

5. For how long will protected health information about me be used or shared with others?

There is no scheduled date at which your protected health information that is being used or shared for this research will be destroyed, because research is an ongoing process.

6. Statement of privacy rights:

- You have the right to withdraw your permission for the doctors and researchers to use or share your protected health information. We will not be able to withdraw all the information that already has been used or shared with others to carry out related activities such as oversight, or that is needed to ensure quality of the study. To withdraw your permission, you must do so in writing by contacting the researcher listed above in the section: “Whom do I contact if I have questions about the research study?” If you do so, you will no longer be a participant in this study. You can withdraw your permission for the optional parts of the biobank and still be in the biobank.
- After your information is shared with the Network, federal law will no longer require it be protected. The Network may also share your information with other people who the federal law does not apply too. The Network will nonetheless only use or disclose your information as described in this form and will have its own security measures in place to ensure your information and privacy remains protected.

Q. Participation Information

If you decide to sign this consent form, we will ask you for information about contacting your physicians and the hospitals that you were treated at for Castleman. We will not disclose details about the results of your participation in this study with any of the individuals that we contact, but rather ask them to provide us with your medical history and your tissue samples.

QUORUM REVIEW
APPROVED
INSTITUTIONAL
REVIEW BOARD

R. Consent

I have read this form, and I have had the opportunity ask questions about this study. I understand that my participation is voluntary, and if I decide not to participate it will have no impact on my medical care. If decide to participate now, I can decide to stop being in the study at any time. I voluntarily agree to take part in this research biobank. By signing this form, I do not give up any of my legal rights.

I Consent

X Yes I Consent

REQUIRED ACTIVITIES:

I agree to the following activities as part of this biobank. (You must agree to all of the following to participate in the biobank. If you do not agree to all of these, you should not join the biobank.)

You can request my stored tissue samples (blood and/or tissue (lymph nodes, bone marrow)) from my doctors and hospitals. You can perform (or collaborate with others to perform) research on the samples, and store the samples until this research study is complete.

_ Yes I Agree

You can request my tissue samples (blood and/or tissue (lymph nodes, bone marrow)) from my currently scheduled medical procedures from my doctor and hospital. You can perform (or collaborate with others to perform) research on the samples, and store the samples until this research study is complete.

_ Yes I Agree

I am willing to provide leftovers from any future tissue samples that may be taken for my regular medical care (blood and/or tissue (lymph nodes, bone marrow)) to the biobank. I will allow research to be performed on the samples, and the samples to be stored until this research study is complete. (Even if you check this now, you can change your mind in the future.)

_ Yes I Agree

You can request my medical records from my doctors and the hospitals and other places where I received and/or continue to receive my treatment. You can link results of the research you perform on my samples with my medical information from my medical records.

_ Yes I Agree

I am willing to be re-contacted by study staff later to update my medical information. Even if I agree now by checking this box, I can decide not to provide this information when I am re-contacted.

QUORUM REVIEW
APPROVED
INSTITUTIONAL
REVIEW BOARD

_ Yes I Agree

You can use the results of the research on my samples and my medical information for future research studies, including studies that have not yet been designed, studies for diseases other than Castleman Disease, and/or studies that may be for commercial purposes.

_ Yes I Agree

You can share the results of the research on my samples and my medical information with central data banks (e.g., the NIH) and with other qualified researchers. It will be shared in a manner that does not include my name, social security number, or any other information that could be used to readily identify me. It can be used by other qualified researchers to perform future research studies, including studies that have not yet been designed, studies for diseases other than Castleman Disease, and studies that may be for commercial purposes.

_ Yes I Agree

OPTIONAL ACTIVITIES:

Please check below if you are willing to allow any of these optional activities as part of this research biobank. You can still be in the study even if you do not agree to any of these activities:

I am willing to have blood drawn for the biobank. You can perform (or collaborate with others to perform) research on the blood sample that I will send you and store the sample until this research study is complete.

_ Yes

I am willing to have my saliva collected for the biobank. You can perform (or collaborate with others to perform) research on the saliva sample that I will send you and store the sample until this research study is complete.

_ Yes

I am willing to be re-contacted by study staff later to provide new samples for research. Even if I agree now by checking this box, I can decide not to provide this information or additional samples when I am re-contacted.

_ Yes

You may re-contact me to notify me about clinical trials and other research opportunities.

_ Yes

QUORUM REVIEW
APPROVED
INSTITUTIONAL
REVIEW BOARD

I am willing to be re-contacted to obtain my consent in the event that return of my personal research results becomes possible.

_ Yes

You should print a copy of this form for your records.

Signature of Parent or Legal Guardian

First

Last

Date of Parent or Legal Guardian Signature

MM/DD/YYYY

Relationship of Parent/Guardian to Participant

--NEXT PAGE--

If you selected “Parent of pediatric CD patient,” at the beginning of this survey please respond on behalf of the PATIENT.

If you selected “Adult CD patient”/ “Related family member of CD patient”/ “Patient with related disease”/ “Healthy volunteer,” at the beginning of this survey, please enter YOUR information.

Please provide your current legal first name.

Please provide your current legal middle name. Please type in 0 if none.

Please provide your current legal last name.

QUORUM REVIEW
APPROVED
INSTITUTIONAL
REVIEW BOARD

Has your name changed since birth?

_ Yes

_ No

What is your date of birth?

MM/DD/YYYY

What was your physical sex at birth?

_Male

_Female

What city/municipality were you born in?

What country were you born in?

Please provide your current full mailing address below.

Street Address

Address Line 2

City

State / Province / Region

Postal / Zip Code

Country

QUORUM REVIEW
APPROVED
INSTITUTIONAL
REVIEW BOARD

_ Which of the following selections describes your ancestry? Please select all that apply.

_ American Indian or Alaska Native

_ Asian

_ Black or African American

_ Hispanic or Latino

_ Native Hawaiian or Other Pacific Islander

_ Caucasian, Non-Finnish Ancestry

_ Caucasian, Finnish Ancestry

_ I prefer not to say

_ Other (please specify)

Please enter your primary phone number where we may contact you if needed.

Please enter your email address.

--NEXT PAGE—

If you selected "Parent of pediatric CD patient," at the beginning of this survey please respond on behalf of the PATIENT.

If you selected "Adult CD patient"/ "Related family member of CD patient"/ "Patient with related disease"/ "Healthy volunteer," at the beginning of this survey, please enter YOUR information.

Are you interested in donating a blood sample?

_ Yes

_ No

Are you interested in having your existing tissue block (lymph node and/or bone marrow) transferred?

_ Yes

_ No

QUORUM REVIEW
APPROVED
INSTITUTIONAL
REVIEW BOARD

If you answered “yes” to either of the previous questions, could you please provide the name and city/state of all of the hospitals where you may have samples stored (Please list all hospitals even if you are unsure if samples remain)?

--NEXT PAGE—

This is the next section of the CDCN Survey.

Please select "Next Page" to continue with the survey. Depending on your prior response, certain pages will not apply to you and you will automatically bypass them.

--NEXT PAGE—

If you selected “Parent of pediatric CD patient,” at the beginning of this survey please respond on behalf of the PATIENT.

If you selected “Adult CD patient”/ “Related family member of CD patient”/ “Patient with related disease”/ “Healthy volunteer,” at the beginning of this survey, please enter YOUR information.

Please list full name of all physicians and institutions.

What is the name of your current Castleman disease treating physician(s) and their corresponding institution(s)?

Please list any other treating physician(s) and their corresponding institution(s) that you have seen since you Castleman disease symptoms started?

QUORUM REVIEW
APPROVED
INSTITUTIONAL
REVIEW BOARD

Have you had a lymph node biopsy pathology report state "consistent with", "suggestive of", "compatible with", or "diagnostic of" Castleman disease?

_ Yes

_ No

Has your clinician diagnosed you with unicentric (enlargement of a single region of lymph nodes) or multicentric (enlargement of more than one region of lymph nodes) Castleman disease?

_ Unicentric

_ Multicentric

_ Unsure

What date were you diagnosed?

MM/DD/YYYY

Have you been told that you have or may have TAFRO syndrome?

_ Yes

_ No

_ Unsure

Have you ever been diagnosed with HIV?

_ Yes

_ No

_ I do not feel comfortable answering this question (prefer not to answer)

Has your clinician told you if your disease is HHV-8 positive, HHV-8-negative, or do you not know?

_ HHV-8-positive

QUORUM REVIEW
APPROVED
INSTITUTIONAL
REVIEW BOARD

_ HHV-8-negative

_ Do Not Know

If you had a HHV-8 test done, which test was performed to determine your HHV-8 status?

_ LANA 1

_ PCR of blood

_ PCR of Lymph node

_ Unsure

Which of the following clinical features have you experienced during active disease/flare? Please select "None" if you did not experience any.

_ None

Clinical features experienced during active disease/flare:

_ Flu-like symptoms

_ Night sweats

_ Fevers (>100.5F)

_ Weight loss

_ Fatigue

_ Large spleen and/or liver

_ Edema/anasarca, effusions, ascites, or other form of fluid overload

_ Eruption of cherry hemangiomas on the skin or violaceous papules

_ Lymphocytic Interstitial pneumonitis

Do you have any other symptoms believed to be related to Castleman disease? If so, please list these symptoms. Also, please comment on the severity of these symptoms and/or the above symptoms. Please type in NA if this does not apply to you.

QUORUM REVIEW
APPROVED
INSTITUTIONAL
REVIEW BOARD

Which of the following laboratory abnormalities have you experienced during active disease/flare? Please select "None" if you did not experience any.

_ None

Laboratory abnormalities experienced during active disease/flare: *

_ Elevated C-Reactive Protein (CRP) (>10mg/dL)

_ Elevated Erythrocyte Sedimentation Rate (ESR) (>15mm/hr)

_ Low Hemoglobin/Anemia (Hgc<12.5 for males, <11.5 for females)

_ Elevated Platelets/Thrombocytosis (>400k/uL)

_ Low Platelets/Thrombocytopenia (<150K/uL)

_ Low Albumin/Hypoalbuminemia (<3.5 gm/dL)

_ Kidney dysfunction (elevated Creatinine or BUN) or proteinuria (protein in urine)

_ High Gamma Globin

_ Levels/Hypergammaglobulinemia (>1700mg/dL)

Do you have any other laboratory abnormalities believed to be related to Castleman disease? If so, please list these symptoms. Also, please comment on the severity of these laboratory abnormalities and/or the above laboratory abnormalities. Please type in NA if this does not apply to you.

Did you have a bone marrow biopsy during active disease/flare? Please specify if the pathology report stated fibrosis, myelofibrosis, or reticulin fibrosis.

_ Yes, stated fibrosis/myelofibrosis/reticulin fibrosis

_ Yes, did not specify

_ No, did not have bone marrow biopsy done

_ Unsure

Have you ever been hospitalized for Castleman disease or associated complications? If so, for how long?

_ Never been hospitalized

_ Less than 3 days

QUORUM REVIEW
APPROVED
INSTITUTIONAL
REVIEW BOARD

_ 3 – 6 days

_ 1 – 4 weeks

_ More than 4 weeks

Please select any disorders listed below that you have been diagnosed with or select "Other" if you have been diagnosed with a disease not on this list.

_ Clinical Epstein Barr Virus (EBV)

_ lymphoproliferative disorders

_ Cytomegalovirus (CMV)

_ Toxoplasmosis

_ Active Tuberculosis

_ Systemic lupus erythematosus

_ Rheumatoid arthritis

_ Adult-onset Still's disease

_ Juvenile Idiopathic Arthritis

_ IgG4-related disease

_ Hodgkin's lymphoma

_ Non-Hodgkin's lymphoma

_ Multiple myeloma

_ Primary lymph node plasmacytoma

_ Follicular dendritic cell sarcoma

_ POEMS syndrome

_ Other (Please Specify)

If you selected "Other", please specify the disease(s).

Please supply any reports or medical records associated with those diagnoses if you have any.

[FILE UPLOAD BUTTON]

QUORUM REVIEW
APPROVED
INSTITUTIONAL
REVIEW BOARD

--NEXT PAGE—

If you selected “Parent of pediatric CD patient,” at the beginning of this survey please respond on behalf of the PATIENT.

If you selected “Adult CD patient”/ “Related family member of CD patient”/ “Patient with related disease”/ “Healthy volunteer,” at the beginning of this survey, please enter YOUR information.

What was the first treatment regimen you received for your Castleman disease (CD)? (Examples of treatment regimen include Prednisone, Siltuximab, Siltuximab+ Prednisone, CHOP, Tocilizumab, Tocilizumab+Prednisone, Surgery, Radiation, Stem Cell Transplant, etc.)

What was the date you began your first treatment regimen?

MM/DD/YYYY

Is this first treatment regimen still ongoing?

_ Yes

X No

What is the date you discontinued this treatment regimen?

MM/DD/YYYY

Do you believe that your symptoms/laboratory values improved on treatment completely, partially, or not at all as a result of your first treatment regimen?

_ Complete improvement in Castleman disease symptoms

X Partial improvement in Castleman disease symptoms

_ No improvement in Castleman disease symptoms

If you improved on your first treatment regimen, did you ever experience progression of the disease at a later time?

_ Yes

_ No

QUORUM REVIEW
APPROVED
INSTITUTIONAL
REVIEW BOARD

What was the date that your disease progressed?

MM/DD/YYYY

Have you received a second treatment regimen?

X Yes

_ No

What was the second treatment regimen you received for your Castleman disease (CD)? (Examples of treatment regimen include Prednisone, Siltuximab, Siltuximab+ Prednisone, CHOP, Tocilizumab, Tocilizumab+Prednisone, Surgery, Radiation, Stem Cell Transplant, etc.)

What was the date you began your second treatment regimen?

MM/DD/YYYY

Is this second treatment regimen still ongoing?

X Yes

_ No

What is the date you discontinued this treatment regimen?

MM/DD/YYYY

Do you believe that your symptoms/laboratory values improved on treatment completely, partially, or not at all as a result of your first treatment regimen?

_ Complete improvement in Castleman disease symptoms

X Partial improvement in Castleman disease symptoms

_ No improvement in Castleman disease symptoms

QUORUM REVIEW
APPROVED
INSTITUTIONAL
REVIEW BOARD

If you improved on your second treatment regimen, did you ever experience progression of the disease at a later time?

X Yes

_ No

What was the date that your disease progressed?

MM/DD/YYYY

Have you received a third treatment regimen?

_ Yes

X No

--NEXT PAGE—

If you selected "Parent of pediatric CD patient," at the beginning of this survey please respond on behalf of the PATIENT.

If you selected "Adult CD patient"/ "Related family member of CD patient"/ "Patient with related disease"/ "Healthy volunteer," at the beginning of this survey, please enter YOUR information.

When was the last time that you had blood work done?

MM/DD/YYYY

When was the last time that you visited your physician?

MM/DD/YYYY

Please upload a copy of the results of your most recent blood work or clinical note here if you have a copy

[FILE UPLOAD BUTTON]

QUORUM REVIEW
APPROVED
INSTITUTIONAL
REVIEW BOARD

At time of this blood draw, do you have active disease/flare (e.g. presence of symptoms related to your Castleman disease)?

X Yes

_ No

At what stage of the disease do you feel you are in currently?

_ Early stages of disease activity

_ At the peak of disease activity

_ Improving since worst disease activity

When was the last time you experienced active disease/flare (presence of symptoms related to your Castleman disease)?

MM/DD/YYYY

Which of the following clinical features are you currently experiencing? Please select "None" if you have not experienced any or select the items from the corresponding list.

_ None

Clinical features "Currently" experienced:

_ Flu-like symptoms

_ Night sweats

_ Fevers (>100.5F)

_ Weight loss

_ Fatigue

_ Large spleen and/or liver

_ Edema/anasarca, effusions, ascites, or other form of fluid overload

_ Eruption of cherry hemangiomas on the skin or violaceous papules

_ Lymphocytic Interstitial pneumonitis

QUORUM REVIEW
APPROVED
INSTITUTIONAL
REVIEW BOARD

Which of the following laboratory abnormalities did you experience when your blood was last drawn? Please select "None" if you have not experienced any or select the items from the corresponding list.

_ None

Laboratory abnormalities experience when your blood was last drawn:

_ Elevated C-Reactive Protein (CRP) (>10mg/dL)

_ Elevated Erythrocyte Sedimentation Rate (ESR) (>15mm/hr)

_ Low Hemoglobin/Anemia (Hgc<12.5 for males, <11.5 for females)

_ Elevated Platelets/Thrombocytosis (>400k/uL)

_ Low Platelets/Thrombocytopenia (<150K/uL)

_ Low Albumin/Hypoalbuminemia (<3.5 gm/dL)

_ Kidney dysfunction (elevated Creatinine or BUN) or proteinuria (protein in urine)

_ High Gamma Globin

_ Levels/Hypergammaglobulinemia (>1700mg/dL)

Are you currently registered in the ACCELERATE registry? (Please visit http://www.cdcn.org/accelerate to get more details if you are interested in joining.)

_ Yes

_ No

Are you currently on any medications (for Castleman disease or other conditions)?

X Yes

_ No

Please list any medications that you are currently taking.

--NEXT PAGE—

QUORUM REVIEW
APPROVED
INSTITUTIONAL
REVIEW BOARD

Thank you for completing our survey! We really appreciate your support in fighting back against Castleman disease.

The CDCN will review the information you have provided and determine how you can be involved in providing samples for research. We will be in touch once we have gathered enough information to be able to send you a kit in the mail with empty tubes or if we have any questions for you.

QUORUM REVIEW
APPROVED
INSTITUTIONAL
REVIEW BOARD

Parent of pediatric CD patient > = 14 < 18 (minors)

Thank you for filling out this Survey, so that we will be able to include your samples in the BioBank.

If you are presented with a Survey Page that does not contain any questions, please select "Next Page" to continue through the Survey. That particular Survey Page does not contain any questions that pertain to you based on your prior responses.

If you have any questions, please contact Dustin Shilling at 1-215-614-0689
or dustin@castlemannetwork.org

The following form will enable you to participate in the CDCN Biobank (Castlebank). Tissue samples such as blood or excess biopsy samples from patients will enable scientists to better characterize and understand Castleman disease and develop better treatments. Any blood or tissue donation will be used for high-impact research to battle this disease!

Is this your first time filling out this Survey? (If you do not remember if you have participated in the past, please call 1-215-614-0689)

X First Time Participant

_ Repeat Participant

--NEXT PAGE--

Please select the appropriate category that describes the PERSON completing this survey. (This question refers to the person completing the survey, NOT necessarily the patient)

_ Adult CD patient (> 18 years old)

X Parent of pediatric CD patient

_ Related family member of CD patient (without CD and > 18 and NOT parent of pediatric CD patient)

_ Patient with related disease (e.g. Lupus, Rheumatoid Arthritis and > 18 years old)

_ Healthy volunteer (> 18 years old)

Please select the appropriate age band for the patient. (Please let us know the age band of the patient. This is needed to determine the consent requirements.)

X Patient > = 14 or < 18

_ Patient > = 7 or < 14

QUORUM REVIEW
APPROVED
INSTITUTIONAL
REVIEW BOARD

_ Patient > 7

RESEARCH CONSENT/ASSENT FORM – KEY POINTS

For Adult Participants or Parents/Guardians

For Minors Age 14 and Older

For Participants Who Become Adults While in the Study

Study Title: The Castleman Disease Collaborative Network Biobank: A Collection of Biospecimens and Clinical Data to Facilitate Research
Study #: 001
Sponsor: Castleman Disease Collaborative Network
Study Investigator: David Fajgenbaum
Castleman Disease Collaborative Network
1001 S. 18th St., Unit A., Philadelphia, PA 19146
Telephone Number: (610) 304-0696

Potential Participants 18 years and older: This is a consent form. It provides a summary of the information the research team will discuss with you. If you decide that you would like to take part in this research study, you would sign this form to confirm your decision.

Potential Teen Participants: This form also serves as an assent form. That means that if you choose to take part in this research study, you would sign this form to confirm your choice. Your parent or guardian would also need to give their permission and sign this form for you to join the study.

Parents/Guardians: You have the option of having your child or teen join a research study. This is a parental permission form. It provides a summary of the information the research team will discuss with you. If you decide that your child can take part in this study, you would sign this form to confirm your decision.

When reading this form, please note that the words “you” and “your” refer to the person in the project rather than to a parent or guardian who might sign this form on behalf of the person in the project.

Participants who become adults during the study: Because you are now an adult, you have been asked to read this consent form. After you have read the form, you will be asked to sign it if you still agree to have your samples and information stored and used in a variety of research studies.

The goal of the “Castleman Disease Collaborative Network Biobank” project is to be a patient-driven movement that empowers people with Castleman Disease to directly transform research and treatment of disease by providing blood, sharing part of their stored tissue, and copies of their medical records for

QUORUM REVIEW
APPROVED
INSTITUTIONAL
REVIEW BOARD

use in a variety of research projects. Because we are enrolling people with Castleman across the country regardless of where they are being treated, this study will allow many more people to contribute to research than has previously been possible.

1. What is the purpose of this study?

We want to understand Castleman Disease better so that we can develop more effective therapies. The Castleman Disease Collaborative Network would like to partner directly with people with Castleman Disease, so we can study many more aspects of Castleman Disease than would otherwise be possible.

2. What will I have to do if I agree to participate in this study?

You will answer some questions about your disease and medical care and send a blood sample and/or a saliva sample to us in a pre-stamped package that we will provide. The blood sample should be collected during your regularly scheduled clinical visit if possible. We will take care of obtaining copies of your medical records as well as small amounts of your stored tissues (if available) from the hospitals or centers where you receive your medical care.

3. Do I have to participate in this study?

No. Taking part in this study is voluntary. Even if you decide to participate, you can always change your mind and leave the study.

4. Will I benefit from participating?

While taking part in this study will not improve your own health, the information we collect will aid in research efforts to provide better treatment and prevention options to people with Castleman in the future. We will provide updates about key research discoveries made possible by this biobank on our website, and directly to you via email if you so choose.

5. What are the risks of taking part in this research?

There may be a risk that your information (which includes your genetic information and information from your medical records) could be seen by unauthorized individuals. However, we have procedures and security measures in place designed to minimize this risk and protect the confidentiality of your information.

Investigators do not plan on returning the research test results to you, and the results will not be placed in your regular medical record. However, if a research test reveals something that could potentially affect your care (for example, a possible misdiagnosis), they may contact your physician to have the tests confirmed in a clinical lab. Your physician, not the investigator, will then follow-up with you. We

QUORUM REVIEW
APPROVED
INSTITUTIONAL
REVIEW BOARD

will obtain consent from you to provide your physician your research results and ask you for updated physician contact information at that time.

If you decide to give blood for this biobank, you may experience pain, bruising, dizziness, or an infection.

6. Will it cost me anything to participate in this study?

No.

7. Who will use my samples and see my information?

Your samples and health information will be available to researchers at the Castleman Disease Collaborative Network (CDCN), a not-for-profit global initiative dedicated to accelerating research and treatment for Castleman disease. Your samples may also be used by outside researchers, both in the U.S. and internationally. After removing your name and other readily identifiable information, we will share tissues and information with qualified researchers, who have undergone review and approval by a CDCN Advisory Board. Results from the research studies may be shared with the greater research community via publications, as well as central data banks at the National Institutes of Health.

8. Can I stop taking part in this research study?

Yes, you can withdraw from this research study at any time. CDCN will destroy all your samples in its possession; if samples and information have already been sent to researchers, the CDCN will notify the researcher of the participant's wishes but the CDCN cannot guarantee these samples will be destroyed. In addition, research data may have already been published before you withdrew and that cannot be retracted. Your information would be removed from future studies.

9. What if I have questions?

If you have any questions, please contact either the Principal Investigator, Dr. David Fajgenbaum at (215) 614-0936. In case you want to inquire via writing, their address is 1001 S. 18th St, A Philadelphia PA 19146.

FULL RESEARCH CONSENT FORM

Adult Participants or Parents/Guardians

The Castleman Disease Collaborative Network Biobank: A Collection of Biospecimens and Clinical Data to Facilitate Research

QUORUM REVIEW
APPROVED
INSTITUTIONAL
REVIEW BOARD

A. Introduction

You are being invited to participate in a research study that will establish a Castleman Disease biobank. A biobank, sometimes called a biorepository, collects, stores, analyzes and distributes samples of human tissues, blood, and related health information to qualified scientists. Creation of a biobank for this disease may help doctors and researchers better understand why Castleman Disease occurs and develop ways to better treat and prevent it. Because Castleman Disease is so rare, it has not been well studied, due in part to a lack of available biospecimens and data for research use.

You are being asked to participate in the study because you have Castleman Disease. With your decision to participate, the CDCN will have access to your already existing tissues (blood, bone marrow, and/or lymph node) from your hospital. You also have the ability to provide additional blood or saliva samples.

The biobank is being established by the Castleman Disease Collaborative Network (CDCN), a global initiative dedicated to accelerating research and treatment for Castleman Disease. We work to achieve this by facilitating collaboration among the global research community, mobilizing resources, strategically investing in high-impact research, and supporting people with Castleman Disease and their family members. This biobank is paid for by the CDCN.

This form describes the project in order to help you decide if you want to participate. This form will tell you what you will have to do and the risks and benefits of participating.

If you have any questions about or do not understand something in this form, you should ask the research staff. You should also discuss your participation with anyone you choose in order to better understand this project and your options. Do not sign this form unless the staff has answered your questions and you decide that you want to be part of this project.

Participating in research is not the same as getting regular medical care. The purpose of regular medical care is to improve your health. The purpose of medical research is to advance the understanding and treatment of disease. Participating in this project does not replace your regular medical care.

B. What is the purpose of this biobank?

The Castleman Disease Collaborative Network is creating this biobank to support research. We want scientists to better understand Castleman Disease and work toward developing more effective therapies. We would like to partner directly with people with Castleman, so we can study many more aspects of Castleman than has previously been possible. In addition, because we are enrolling people with Castleman across the country regardless of where they are being treated, this study will allow many more people to directly contribute to research than might otherwise be feasible.

By participating in this biobank project, you will be asked to provide blood or saliva now or in the future, tissue (lymph node, bone marrow samples) left over from past medical tests, and/or medical information to be used for research. We would also like your permission to access and share (after removing your name and other personal identifiers) your medical information along with your specimens.

QUORUM REVIEW
APPROVED
INSTITUTIONAL
REVIEW BOARD

Some of the research might involve studying DNA contained in your samples. DNA is the material that makes up genes. Genes determine things like hair color and eye color, and may play a role in Castleman Disease. Using DNA from your blood or tissue sample, researchers will study your entire genetic sequence, known as your genome, and store this information. Your genomic data and health information will be studied along with information from other participants in this study, and it will be stored for future studies.

Some of the research might involve creating and distributing cell lines from your tissue so that your cells can grow indefinitely to be used in various projects. Cell lines may be useful because of the characteristics of the cells and/or the products they may produce.

Samples may be studied immediately or may be stored for future use by researchers from universities, the government, health-related companies, and other research institutes. Some researchers will be from the United States and some may be from other countries around the world. A Biobank Advisory Board (BAB) composed of physicians and scientists will review research requests to help ensure that tissues are being utilized in the most impactful manner to aid our understanding of Castleman Disease. The BAB will not to attempt to re-identify you. Future researchers must also sign an agreement they will not attempt to re-identify you. Because technology is changing all the time it is not possible to know all of the techniques that researchers may use now or in the future.

We are asking many people to participate in the biobank, both people with Castleman, their family members, and healthy individuals (as a comparison group). We don't know how many people will participate.

C. What other options are there?

Your participation in the biobank project is voluntary. Your alternative is not to participate. The biobank project does not involve treatment for any condition, so your decision to participate or not to participate will not affect your medical care. Your decision not to participate or to stop participating at any time will not involve any penalty to you and will not involve any loss of benefits to which you might otherwise be entitled.

D. What is involved in the biobank project?

With your consent, project staff will collect medical information through a secure questionnaire regarding your health, the doctors you have seen, as well as your medical and family history. Alternatively, a staff member where you receive your care may review your medical records to collect such information. The medical information may include your patient history, response to treatment, pathology (disease) reports, and radiographic (imaging) reports.

The types of samples the Castleman Disease Collaborative Network wants to collect include:

- leftover tissue (such as lymph nodes and/or bone marrow) that have been taken, are scheduled to be taken, or that may be taken in the future, as part of your regular medical care
- blood samples already stored, scheduled to be taken, or that may be taken in the future

QUORUM REVIEW
APPROVED
INSTITUTIONAL
REVIEW BOARD

- saliva

Each type of sample collection is optional. You can decide to participate in each type of sample collection separately, which means you can agree to some, all, or none of the different types of samples. You can indicate your choice at the end of this form.

If you have had surgery in the past and you provide consent, project staff will contact the hospital(s) where you had surgery to get leftover tissue or fluids that may be stored there. If you are scheduled for a medical procedure or surgery for your care, no additional tissue will be taken beyond what is needed for your care. After the medical staff has used your tissue for diagnosis and care, any leftover samples you have agreed to provide will be transferred to the biobank.

If you consent to have blood drawn for the biobank, it may be collected at the same time as other blood tests or during your surgery, if possible. The biobank will send you blood collection kits with tubes and instructions that you can take to your doctor or local laboratory so they can obtain one or more blood samples. The amount of blood collected will be no greater than 50 mL (about 3 tablespoons) over a 2-month period.

We will link the results of the tissue collected with medical information that has been generated during the course of your regular treatment. We are asking your permission to obtain a copy of your medical record from places where you have received care for your disease.

If you give your consent to be re-contacted, biobank staff may contact you periodically to update your medical information or to ask you to provide new samples for research. You may also choose if you would like the Castleman Disease Collaborative Network to notify you about other research studies in the future.

People who work with the sponsor (CDCN) will store your samples in a secure laboratory. Your samples will be stored as long as the sponsor decides to keep them.

E. How long will I be in this research study?

Your direct involvement will be limited initially to the time it takes to obtain your consent and to collect samples and medical information. Because this project involves a biobank, your samples, data, and health information may be used immediately or may be stored indefinitely for future use. If you agree, we may also re-contact you to get updates about your health and medical care, to ask whether you will give additional samples, or to tell you about other studies.

If a participant is a minor when his/her samples are first placed in the biobank, and he/she later becomes an adult, the project staff will contact the participant to seek his/her consent to continue to use the samples in the biobank. At that time, the participant can decide to continue in this biobank project, or ask that the samples be destroyed. If such a participant cannot be contacted, any samples still in the bank will be retained and will continue to be distributed in de-identified form.

If you want to stop being in the biobank project, contact the Principal Investigator or project staff using the contact information provided later in this form. If you decide to withdraw from the project early, the Principal Investigator or project staff may ask you some questions about being in the project to gain

QUORUM REVIEW
APPROVED
INSTITUTIONAL
REVIEW BOARD

feedback on ways in which biobank may be improved. The Principal Investigator or the sponsor can remove you from the project at any time, even if you want to stay in the project.

F. What kind of information could be found in this study and will I be able to see it?

This research is being done to add to our knowledge of Castleman Disease, and is not intended to provide information about your individual health or treatment. Investigators do not plan on returning the research test results to you, and the results will not be placed in your regular medical record. However, if a research test reveals something that could potentially affect your care (such as a possible misdiagnosis) they may contact your physician to have the tests confirmed in a clinical lab. Your physician, not the investigator, will then follow-up with you.

If you choose, the Castleman Disease Collaborative Network can also keep you up to date on the research outcomes of projects using samples or data from this biobank; however not all research projects will use samples from all the donors to the bank, so your own tissue may or may not have been part of a particular research project. You do not need to participate in this biobank to be kept up-to-date on the Castleman Disease Collaborative Network's research efforts; instead, you can sign up online for our email list here at cdcn.org. We regularly update the website.

If you choose, your samples and information may also be used for other central databanks. To allow research collaborations and sharing of data, the National Institutes of Health (NIH) and other research organizations have developed special data (information) repositories that analyze data and collect the results of certain types of genetic studies. These central banks will store your genetic information and provide them to qualified researchers to do more studies. This information will be available for any research question, such as research to understand what causes certain diseases (for example heart disease, cancer, or psychiatric disorders), development of new scientific methods, or the study of where different groups of people may have come from. We are also asking your permission to share your results with these special controlled-access banks. Controlled-access means that only researchers who apply for and get permission to use the information for a specific research project will be able to access the information. Your genomic data and health information will not be labeled with your name or other information that could be used to identify you. Researchers approved to access information in the database will agree not to attempt to identify you. Your information will be sent only with a code number attached. All directly identifiable information, such as your name, will not be shared with data banks or other investigators. Although there may be a small risk of loss of privacy when sharing this information with these banks, we have established procedures to encode your samples and information and protect your data. Although we will do everything we can to protect the privacy of your data, we cannot absolutely guarantee its privacy or predict how genetic information will be used in the future.

We will publish important discoveries found through these studies in the scientific literature so that the entire research community can work together to better understand Castleman Disease. Your individual data will not be published in a way in which you could be readily identified. Abstracts, which are plain language summaries of the published reports, will be available to you and the general public.

G. What are the risks or discomforts of the biobank project?

QUORUM REVIEW
APPROVED
INSTITUTIONAL
REVIEW BOARD

Your regular medical care will not change if you participate in this biobank project. The collection of tissue samples would only take place in the context of your routine care, and therefore does not add any physical risk to you that you would not already be undertaking. If you agree to give an additional blood sample, the medical staff will take your blood by sticking a needle in your arm or they may collect the blood during surgery. The amount of blood that will be collected will not exceed 50 mL (about 3 tablespoons) over a 2-month period. Some problems you might have from this are:

- It may hurt.
- You may get a bruise.
- You may feel dizzy.
- You may get an infection.

There are some risks related to the use of your medical information and specimens. There could be an accidental release of your medical information to people who are not involved in your medical care. An accidental release of your genetic information could be used to identify you and your family members. We have tried to minimize this risk by carefully limiting access to the computers that would house your information and having other strict data security measures in place. There is still a risk someone could get access to the information we have stored about you, including information about a family member (for example, revealing that you or a blood relative carry a genetic disease). Because your DNA information is unique to you, there is a chance that someone could trace it back to you. The risk of this happening is small, but may be greater in the future.

Federal and State laws also provide some protections against discrimination based on genetic information. For example, the Genetic Information Nondiscrimination Act (GINA) makes it illegal for health insurance companies, group health plans, and most employers to discriminate against you based on your genetic information. However, it does not prevent companies that sell life insurance, disability insurance, or long-term care insurance from using genetic information as a reason to deny coverage or set premiums.

H. What are the benefits of the research study?

Research conducted with your information and specimens will not help you directly, but it may help people with Castleman in the future. Research performed with your information and specimens could result in the discovery and development of new drugs, tests or commercial products.

I. Can I stop being in the research study and what are my rights?

You can stop being in the research study at any time. You may choose to leave your samples and/or information in the biobank but request not to be re-contacted. Or, if you want to end all participation, the CDCN will destroy all of your sample identifiers and medical information in its possession; the CDCN will retain your original consent and the documentation of your withdrawal of consent. Any specimens or data that have already been distributed in a de-identified manner cannot be destroyed, and some research data may have already been published. If data has already been distributed to third parties, it

QUORUM REVIEW
APPROVED
INSTITUTIONAL
REVIEW BOARD

cannot be retrieved (as it will have been de-identified). Any specimens or identifiable data you have given can be removed upon request, but any de-identified, already distributed information or specimens cannot be withdrawn.

To withdraw from research, you must notify, in writing, the Principal Investigator (Address: 1001 S. 18th St, A Philadelphia PA 19146). There are three ways you can choose to stop participating in the Biobank:

1. You may choose to leave your data and unused samples in the Biobank while requesting not to be re-contacted in the future.

2. You may request that your personally identifying information be destroyed, but allow some or all of your samples to continue being used anonymously for future research.

3. You can choose to end all participation, in which case all of the unused samples in the Biobank will be destroyed and your medical information will be deleted.

J. Will I be paid to take part in this research study?

You will not get payment for being in this biobank project. The sponsor does not plan to give you any money or payment resulting from any discoveries or products made from this research project.

K. What are the costs?

Participating in the biobank project should not cost you anything beyond your routine, scheduled medical costs. If you decide to donate saliva and blood samples, we recommend you get these drawn during your regularly scheduled clinical appointments. You and your insurance company will already be paying for the costs of the blood draws as a part of your standard medical care. That way there's no additional cost. If you don't have regularly scheduled follow-up but still want to participate, please visit an outpatient clinic to get your blood drawn. You will need to pay for the cost of the blood draw but the CDCN can reimburse you if you fill out a W2 form and provide a copy of the original receipt. If you have a future scheduled biopsy planned, you and your insurance company will already be paying for the costs of that procedure, so there will be no additional cost. Neither you nor your insurance company will be asked to pay for research tests.

L. What happens if I am injured or sick because I took part in this research study?

If injury (physical, psychological, social, financial, or otherwise) occurs as a result of participation in this project, there are no plans for providing compensation to you or your family.

Be aware that your health care payer/insurer might not cover the costs of study-related injuries.

You do not give up any of your legal rights by signing this form.

M. What about confidentiality?

QUORUM REVIEW
APPROVED
INSTITUTIONAL
REVIEW BOARD

Your individual data and health information (without your name, address, or other details that might identify you) will be put in a controlled-access database. This means that only researchers who apply for and get permission to use the information for a specific research project will be able to access the information. Researchers approved to access information in the database will agree not to attempt to identify you. Access to information with your name, such as the consent form, will be restricted to healthcare staff, the Principal Investigator, designated CDCN staff, and approved CDCN contractors. Documents will be kept in a locked cabinet and information in computers will be protected by passwords. In order to protect your private information, only a unique code number, not your name or medical record number, will appear on your samples or on information sent to researchers outside the CDCN. Some people who work for the biobank will be able to find your name from the code so they can possibly have your sample(s) destroyed if you change your mind later. Except when required by law, your personally identifying information will not be disclosed outside of the CDCN.

N. Whom do I contact if I have questions about the research study?

If you have any questions about the study, please contact the research doctor or study staff listed below by email davidfajgenbaum@gmail.com or calling 215-573-8101:

- David Fajgenbaum, MD (Principal Investigator)

If you prefer to do so in writing, please send written correspondence to the address 1001 S. 18th St, A Philadelphia PA 19146.

You can learn more about the Castleman Disease Collaborative Network by visiting our website at www.cdcn.org.

Quorum Review Institutional Review Board (IRB) has reviewed this project. An IRB is a group of people who review research studies to protect the rights and welfare of research participants. If you have questions about your rights as a research participant, if you are not able to resolve your concerns with the Principal Investigator, if you have a complaint, or if you have general questions about what it means to be in a research project, you can call Quorum Review or visit the Quorum Review website at www.quorumreview.com.

Quorum Review is located in Seattle, Washington.

Office hours are 8:00 AM to 5:00 PM Pacific Time, Monday through Friday.

Ask to speak with a Research Participant Liaison at 888-776-9115 (toll free).

O. Conflict of Interest

Dr. David Fajgenbaum, the person responsible for the conduct of this research study also serves as the Executive Director of the CDCN. You should be aware that you are not under any obligation to participate in this research study.

QUORUM REVIEW
APPROVED
INSTITUTIONAL
REVIEW BOARD

P. Authorization to use your health information for research purposes

Because information about you and your health is personal and private, it generally cannot be used in this research study without your written authorization. Federal law called HIPAA requires that your health care providers and healthcare institutions (hospitals, clinics, doctor's offices) protect the privacy of information that identifies you and relates to your past, present, and future physical and mental health conditions.

If you agree to take part in this research, we will request that your health care providers and healthcare institutions disclose your protected health information to the Castleman Disease Collaborative Network for use in this research study. This may mean that you need to sign an additional form specifically authorizing release to the Network, but this will depend on your doctor's local practices, and we will work with you on this.

This section informs you about how your health information will be used or disclosed in the study. Your information will only be used in accordance with what is described here and as required or allowed by law. If you do not want to allow the uses described here, you cannot be in this biobank. You can still be in the biobank even if you do not give permission for the use and sharing of your information for the optional parts of the biobank.

1. What personal information about me will be used or shared with others during this research?

- Your medical records
- Your tissue samples relevant to this research study and related records
- New health information created from study-related tests and/or questionnaires

2. Why will protected information about me be used or shared with others?

The main reasons include the following:

- To conduct and oversee the research described earlier in this form;
- To ensure the research meets legal, institutional, and accreditation requirements;
- To conduct public health activities (including reporting of adverse events or situations where you or others may be at risk of harm)

3. Who will use or share protected health information about me?

With your consent, biobank staff will obtain information about you either directly from you or by having healthcare staff review your medical records. They may share your consent information in connection with the research study.

QUORUM REVIEW
APPROVED
INSTITUTIONAL
REVIEW BOARD

4. With whom outside of the Castleman Disease Collaborative Network may my personal health information be shared?

While all reasonable efforts will be made to protect the confidentiality of your protected health information, it may also be shared with the following entities:

- Federal and state agencies (for example, the Department of Health and Human Services, the Food and Drug Administration, the National Institutes of Health, and/or the Office for Human Research Protections), or other domestic or foreign government bodies if required by law and/or necessary for oversight purposes. A qualified representative of the FDA, the National Cancer Institute, or Quorum Review may review your medical records.
- Outside individuals or entities that have a need to access this information to perform functions relating to the conduct of this research such as data storage companies.

5. For how long will protected health information about me be used or shared with others?

There is no scheduled date at which your protected health information that is being used or shared for this research will be destroyed, because research is an ongoing process.

6. Statement of privacy rights:

- You have the right to withdraw your permission for the doctors and researchers to use or share your protected health information. We will not be able to withdraw all the information that already has been used or shared with others to carry out related activities such as oversight, or that is needed to ensure quality of the study. To withdraw your permission, you must do so in writing by contacting the researcher listed above in the section: “Whom do I contact if I have questions about the research study?” If you do so, you will no longer be a participant in this study. You can withdraw your permission for the optional parts of the biobank and still be in the biobank.
- After your information is shared with the Network, federal law will no longer require it be protected. The Network may also share your information with other people who the federal law does not apply too. The Network will nonetheless only use or disclose your information as described in this form and will have its own security measures in place to ensure your information and privacy remains protected.

Q. Participation Information

If you decide to sign this consent form, we will ask you for information about contacting your physicians and the hospitals that you were treated at for Castleman. We will not disclose details about the results of your participation in this study with any of the individuals that we contact, but rather ask them to provide us with your medical history and your tissue samples.

QUORUM REVIEW
APPROVED
INSTITUTIONAL
REVIEW BOARD

R. Consent

I have read this form, and I have had the opportunity ask questions about this study. I understand that my participation is voluntary, and if I decide not to participate it will have no impact on my medical care. If decide to participate now, I can decide to stop being in the study at any time. I voluntarily agree to take part in this research biobank. By signing this form, I do not give up any of my legal rights.

I Consent

X **Yes I Consent**

REQUIRED ACTIVITIES:

I agree to the following activities as part of this biobank. (You must agree to all of the following to participate in the biobank. If you do not agree to all of these, you should not join the biobank.)

You can request my stored tissue samples (blood and/or tissue (lymph nodes, bone marrow)) from my doctors and hospitals. You can perform (or collaborate with others to perform) research on the samples, and store the samples until this research study is complete.

_ Yes I Agree

You can request my tissue samples (blood and/or tissue (lymph nodes, bone marrow)) from my currently scheduled medical procedures from my doctor and hospital. You can perform (or collaborate with others to perform) research on the samples, and store the samples until this research study is complete.

_ Yes I Agree

I am willing to provide leftovers from any future tissue samples that may be taken for my regular medical care (blood and/or tissue (lymph nodes, bone marrow)) to the biobank. I will allow research to be performed on the samples, and the samples to be stored until this research study is complete. (Even if you check this now, you can change your mind in the future.)

_ Yes I Agree

You can request my medical records from my doctors and the hospitals and other places where I received and/or continue to receive my treatment. You can link results of the research you perform on my samples with my medical information from my medical records.

_ Yes I Agree

I am willing to be re-contacted by study staff later to update my medical information. Even if I agree now by checking this box, I can decide not to provide this information when I am re-contacted.

QUORUM REVIEW
APPROVED
INSTITUTIONAL
REVIEW BOARD

_ Yes I Agree

You can use the results of the research on my samples and my medical information for future research studies, including studies that have not yet been designed, studies for diseases other than Castleman Disease, and/or studies that may be for commercial purposes.

_ Yes I Agree

You can share the results of the research on my samples and my medical information with central data banks (e.g., the NIH) and with other qualified researchers. It will be shared in a manner that does not include my name, social security number, or any other information that could be used to readily identify me. It can be used by other qualified researchers to perform future research studies, including studies that have not yet been designed, studies for diseases other than Castleman Disease, and studies that may be for commercial purposes.

_ Yes I Agree

OPTIONAL ACTIVITIES:

Please check below if you are willing to allow any of these optional activities as part of this research biobank. You can still be in the study even if you do not agree to any of these activities:

I am willing to have blood drawn for the biobank. You can perform (or collaborate with others to perform) research on the blood sample that I will send you and store the sample until this research study is complete.

_ Yes

I am willing to have my saliva collected for the biobank. You can perform (or collaborate with others to perform) research on the saliva sample that I will send you and store the sample until this research study is complete.

_ Yes

I am willing to be re-contacted by study staff later to provide new samples for research. Even if I agree now by checking this box, I can decide not to provide this information or additional samples when I am re-contacted.

_ Yes

You may re-contact me to notify me about clinical trials and other research opportunities.

_ Yes

QUORUM REVIEW
APPROVED
INSTITUTIONAL
REVIEW BOARD

I am willing to be re-contacted to obtain my consent in the event that return of my personal research results becomes possible.

_ Yes

You should print a copy of this form for your records.

Participant Signature

First

Last

Date of Participant Signature

MM/DD/YYYY

Signature of Parent or Legal Guardian

First

Last

Date of Parent or Legal Guardian Signature

MM/DD/YYYY

Relationship of Parent/Guardian to Participant

--NEXT PAGE--

QUORUM REVIEW
APPROVED
INSTITUTIONAL
REVIEW BOARD

If you selected “Parent of pediatric CD patient,” at the beginning of this survey please respond on behalf of the PATIENT.

If you selected “Adult CD patient”/ “Related family member of CD patient”/ “Patient with related disease”/ “Healthy volunteer,” at the beginning of this survey, please enter YOUR information.

Please provide your current legal first name.

Please provide your current legal middle name. Please type in 0 if none.

Please provide your current legal last name.

Has your name changed since birth?

_Yes

_ No

What is your date of birth?

MM/DD/YYYY

What was your physical sex at birth?

_ Male

_Female

What city/municipality were you born in?

What country were you born in?

Please provide your current full mailing address below.

Street Address

QUORUM REVIEW
APPROVED
INSTITUTIONAL
REVIEW BOARD

Address Line 2

City

State / Province / Region

Postal / Zip Code

Country

_ Which of the following selections describes your ancestry? Please select all that apply.

_ American Indian or Alaska Native

_ Asian

_ Black or African American

_ Hispanic or Latino

_ Native Hawaiian or Other Pacific Islander

_ Caucasian, Non-Finnish Ancestry

_ Caucasian, Finnish Ancestry

_ I prefer not to say

_ Other (please specify)

Please enter your primary phone number where we may contact you if needed.

Please enter your email address.

--NEXT PAGE—

If you selected "Parent of pediatric CD patient," at the beginning of this survey please respond on behalf of the PATIENT.

QUORUM REVIEW
APPROVED
INSTITUTIONAL
REVIEW BOARD

If you selected “Adult CD patient”/ “Related family member of CD patient”/ “Patient with related disease”/ “Healthy volunteer,” at the beginning of this survey, please enter YOUR information.

Are you interested in donating a blood sample?

_ Yes

_ No

Are you interested in having your existing tissue block (lymph node and/or bone marrow) transferred?

_ Yes

_ No

If you answered “yes” to either of the previous questions, could you please provide the name and city/state of all of the hospitals where you may have samples stored (Please list all hospitals even if you are unsure if samples remain)?

--NEXT PAGE—

This is the next section of the CDCN Survey.

Please select "Next Page" to continue with the survey. Depending on your prior response, certain pages will not apply to you and you will automatically bypass them.

--NEXT PAGE—

If you selected “Parent of pediatric CD patient,” at the beginning of this survey please respond on behalf of the PATIENT.

If you selected “Adult CD patient”/ “Related family member of CD patient”/ “Patient with related disease”/ “Healthy volunteer,” at the beginning of this survey, please enter YOUR information.

Please list full name of all physicians and institutions.

QUORUM REVIEW
APPROVED
INSTITUTIONAL
REVIEW BOARD

What is the name of your current Castleman disease treating physician(s) and their corresponding institution(s)?

Please list any other treating physician(s) and their corresponding institution(s) that you have seen since you Castleman disease symptoms started?

Have you had a lymph node biopsy pathology report state "consistent with", "suggestive of", "compatible with", or "diagnostic of" Castleman disease?

_ Yes

_ No

Has your clinician diagnosed you with unicentric (enlargement of a single region of lymph nodes) or multicentric (enlargement of more than one region of lymph nodes) Castleman disease?

_ Unicentric

_ Multicentric

_ Unsure

What date were you diagnosed?

MM/DD/YYYY

Have you been told that you have or may have TAFRO syndrome?

_ Yes

QUORUM REVIEW
APPROVED
INSTITUTIONAL
REVIEW BOARD

_ No

_ Unsure

Have you ever been diagnosed with HIV?

_ Yes

_ No

_ I do not feel comfortable answering this question (prefer not to answer)

Has your clinician told you if your disease is HHV-8 positive, HHV-8-negative, or do you not know?

_ HHV-8-positive

_ HHV-8-negative

_ Do Not Know

If you had a HHV-8 test done, which test was performed to determine your HHV-8 status?

_ LANA 1

_ PCR of blood

_ PCR of Lymph node

_ Unsure

Which of the following clinical features have you experienced during active disease/flare? Please select "None" if you did not experience any.

_ None

Clinical features experienced during active disease/flare:

_ Flu-like symptoms

_ Night sweats

_ Fevers (>100.5F)

_ Weight loss

_ Fatigue

QUORUM REVIEW
APPROVED
INSTITUTIONAL
REVIEW BOARD

_ Large spleen and/or liver

_ Edema/anasarca, effusions, ascites, or other form of fluid overload

_ Eruption of cherry hemangiomas on the skin or violaceous papules

_ Lymphocytic Interstitial pneumonitis

Do you have any other symptoms believed to be related to Castleman disease? If so, please list these symptoms. Also, please comment on the severity of these symptoms and/or the above symptoms. Please type in NA if this does not apply to you.

Which of the following laboratory abnormalities have you experienced during active disease/flare? Please select "None" if you did not experience any.

_ None

Laboratory abnormalities experienced during active disease/flare: *

_ Elevated C-Reactive Protein (CRP) (>10mg/dL)

_ Elevated Erythrocyte Sedimentation Rate (ESR) (>15mm/hr)

_ Low Hemoglobin/Anemia (Hgc<12.5 for males, <11.5 for females)

_ Elevated Platelets/Thrombocytosis (>400k/uL)

_ Low Platelets/Thrombocytopenia (<150K/uL)

_ Low Albumin/Hypoalbuminemia (<3.5 gm/dL)

_ Kidney dysfunction (elevated Creatinine or BUN) or proteinuria (protein in urine)

_ High Gamma Globin

_ Levels/Hypergammaglobulinemia (>1700mg/dL)

Do you have any other laboratory abnormalities believed to be related to Castleman disease? If so, please list these symptoms. Also, please comment on the severity of these laboratory abnormalities and/or the above laboratory abnormalities. Please type in NA if this does not apply to you.

Did you have a bone marrow biopsy during active disease/flare? Please specify if the pathology report stated fibrosis, myelofibrosis, or reticulin fibrosis.

QUORUM REVIEW
APPROVED
INSTITUTIONAL
REVIEW BOARD

_ Yes, stated fibrosis/myelofibrosis/reticulin fibrosis

_ Yes, did not specify

_ No, did not have bone marrow biopsy done

_ Unsure

Have you ever been hospitalized for Castleman disease or associated complications? If so, for how long?

_ Never been hospitalized

_ Less than 3 days

_ 3 – 6 days

_ 1 – 4 weeks

_ More than 4 weeks

Please select any disorders listed below that you have been diagnosed with or select "Other" if you have been diagnosed with a disease not on this list.

_ Clinical Epstein Barr Virus (EBV)

_ lymphoproliferative disorders

_ Cytomegalovirus (CMV)

_ Toxoplasmosis

_ Active Tuberculosis

_ Systemic lupus erythematosus

_ Rheumatoid arthritis

_ Adult-onset Still's disease

_ Juvenile Idiopathic Arthritis

_ IgG4-related disease

_ Hodgkin's lymphoma

_ Non-Hodgkin's lymphoma

_ Multiple myeloma

_ Primary lymph node plasmacytoma

QUORUM REVIEW
APPROVED
INSTITUTIONAL
REVIEW BOARD

_ Follicular dendritic cell sarcoma

_ POEMS syndrome

_ Other (Please Specify)

If you selected "Other", please specify the disease(s).

Please supply any reports or medical records associated with those diagnoses if you have any.

[FILE UPLOAD BUTTON]

--NEXT PAGE—

If you selected "Parent of pediatric CD patient," at the beginning of this survey please respond on behalf of the PATIENT.

If you selected "Adult CD patient"/ "Related family member of CD patient"/ "Patient with related disease"/ "Healthy volunteer," at the beginning of this survey, please enter YOUR information.

What was the first treatment regimen you received for your Castleman disease (CD)? (Examples of treatment regimen include Prednisone, Siltuximab, Siltuximab+ Prednisone, CHOP, Tocilizumab, Tocilizumab+Prednisone, Surgery, Radiation, Stem Cell Transplant, etc.)

What was the date you began your first treatment regimen?

MM/DD/YYYY

Is this first treatment regimen still ongoing?

_ Yes

X No

What is the date you discontinued this treatment regimen?

MM/DD/YYYY

QUORUM REVIEW
APPROVED
INSTITUTIONAL
REVIEW BOARD

Do you believe that your symptoms/laboratory values improved on treatment completely, partially, or not at all as a result of your first treatment regimen?

_ Complete improvement in Castleman disease symptoms

X Partial improvement in Castleman disease symptoms

_ No improvement in Castleman disease symptoms

If you improved on your first treatment regimen, did you ever experience progression of the disease at a later time?

_ Yes

_ No

What was the date that your disease progressed?

MM/DD/YYYY

Have you received a second treatment regimen?

X Yes

_ No

What was the second treatment regimen you received for your Castleman disease (CD)? (Examples of treatment regimen include Prednisone, Siltuximab, Siltuximab+ Prednisone, CHOP, Tocilizumab, Tocilizumab+Prednisone, Surgery, Radiation, Stem Cell Transplant, etc.)

What was the date you began your second treatment regimen?

MM/DD/YYYY

Is this second treatment regimen still ongoing?

X Yes

_ No

QUORUM REVIEW
APPROVED
INSTITUTIONAL
REVIEW BOARD

What is the date you discontinued this treatment regimen?

MM/DD/YYYY

Do you believe that your symptoms/laboratory values improved on treatment completely, partially, or not at all as a result of your first treatment regimen?

_ Complete improvement in Castleman disease symptoms

X Partial improvement in Castleman disease symptoms

_ No improvement in Castleman disease symptoms

If you improved on your second treatment regimen, did you ever experience progression of the disease at a later time?

X Yes

_ No

What was the date that your disease progressed?

MM/DD/YYYY

Have you received a third treatment regimen?

_ Yes

X No

--NEXT PAGE—

If you selected "Parent of pediatric CD patient," at the beginning of this survey please respond on behalf of the PATIENT.

If you selected "Adult CD patient"/ "Related family member of CD patient"/ "Patient with related disease"/ "Healthy volunteer," at the beginning of this survey, please enter YOUR information.

When was the last time that you had blood work done?

QUORUM REVIEW
APPROVED
INSTITUTIONAL
REVIEW BOARD

MM/DD/YYYY

When was the last time that you visited your physician?

MM/DD/YYYY

Please upload a copy of the results of your most recent blood work or clinical note here if you have a copy

[FILE UPLOAD BUTTON]

At time of this blood draw, do you have active disease/flare (e.g. presence of symptoms related to your Castleman disease)?

X Yes

_ No

At what stage of the disease do you feel you are in currently?

_ Early stages of disease activity

_ At the peak of disease activity

_ Improving since worst disease activity

When was the last time you experienced active disease/flare (presence of symptoms related to your Castleman disease)?

MM/DD/YYYY

Which of the following clinical features are you currently experiencing? Please select "None" if you have not experienced any or select the items from the corresponding list.

_ None

Clinical features "Currently" experienced:

_ Flu-like symptoms

_ Night sweats

QUORUM REVIEW
APPROVED
INSTITUTIONAL
REVIEW BOARD

_ Fevers (>100.5F)

_ Weight loss

_ Fatigue

_ Large spleen and/or liver

_ Edema/anasarca, effusions, ascites, or other form of fluid overload

_ Eruption of cherry hemangiomas on the skin or violaceous papules

_ Lymphocytic Interstitial pneumonitis

Which of the following laboratory abnormalities did you experience when your blood was last drawn? Please select "None" if you have not experienced any or select the items from the corresponding list.

_ None

Laboratory abnormalities experience when your blood was last drawn:

_ Elevated C-Reactive Protein (CRP) (>10mg/dL)

_ Elevated Erythrocyte Sedimentation Rate (ESR) (>15mm/hr)

_ Low Hemoglobin/Anemia (Hgc<12.5 for males, <11.5 for females)

_ Elevated Platelets/Thrombocytosis (>400k/uL)

_ Low Platelets/Thrombocytopenia (<150K/uL)

_ Low Albumin/Hypoalbuminemia (<3.5 gm/dL)

_ Kidney dysfunction (elevated Creatinine or BUN) or proteinuria (protein in urine)

_ High Gamma Globin

_ Levels/Hypergammaglobulinemia (>1700mg/dL)

Are you currently registered in the ACCELERATE registry? (Please visit http://www.cdcn.org/accelerate to get more details if you are interested in joining.)

_ Yes

_ No

Are you currently on any medications (for Castleman disease or other conditions)?

QUORUM REVIEW
APPROVED
INSTITUTIONAL
REVIEW BOARD

X Yes

_ No

Please list any medications that you are currently taking.

--NEXT PAGE—

Thank you for completing our survey! We really appreciate your support in fighting back against Castleman disease.

The CDCN will review the information you have provided and determine how you can be involved in providing samples for research. We will be in touch once we have gathered enough information to be able to send you a kit in the mail with empty tubes or if we have any questions for you.

QUORUM REVIEW
APPROVED
INSTITUTIONAL
REVIEW BOARD

Parent of pediatric CD patient > = 7 < 14 (minors)

Thank you for filling out this Survey, so that we will be able to include your samples in the BioBank.

If you are presented with a Survey Page that does not contain any questions, please select "Next Page" to continue through the Survey. That particular Survey Page does not contain any questions that pertain to you based on your prior responses.

If you have any questions, please contact Dustin Shilling at 1-215-614-0689
or dustin@castlemannetwork.org

The following form will enable you to participate in the CDCN Biobank (Castlebank). Tissue samples such as blood or excess biopsy samples from patients will enable scientists to better characterize and understand Castleman disease and develop better treatments. Any blood or tissue donation will be used for high-impact research to battle this disease!

Is this your first time filling out this Survey? (If you do not remember if you have participated in the past, please call 1-215-614-0689)

X First Time Participant

_ Repeat Participant

--NEXT PAGE--

Please select the appropriate category that describes the PERSON completing this survey. (This question refers to the person completing the survey, NOT necessarily the patient)

_ Adult CD patient (> 18 years old)

X Parent of pediatric CD patient

_ Related family member of CD patient (without CD and > 18 and NOT parent of pediatric CD patient)

_ Patient with related disease (e.g. Lupus, Rheumatoid Arthritis and > 18 years old)

_ Healthy volunteer (> 18 years old)

Please select the appropriate age band for the patient. (Please let us know the age band of the patient. This is needed to determine the consent requirements.)

_ Patient > = 14 or < 18

X Patient > = 7 or < 14

QUORUM REVIEW
APPROVED
INSTITUTIONAL
REVIEW BOARD

_ Patient > 7

RESEARCH CONSENT/ASSENT FORM – KEY POINTS

For Adult Participants or Parents/Guardians

For Minors Age 14 and Older

For Participants Who Become Adults While in the Study

Study Title: The Castleman Disease Collaborative Network Biobank: A Collection of Biospecimens and Clinical Data to Facilitate Research
Study #: 001
Sponsor: Castleman Disease Collaborative Network
Study Investigator: David Fajgenbaum
Castleman Disease Collaborative Network
1001 S. 18th St., Unit A., Philadelphia, PA 19146
Telephone Number: (610) 304-0696

Potential Participants 18 years and older: This is a consent form. It provides a summary of the information the research team will discuss with you. If you decide that you would like to take part in this research study, you would sign this form to confirm your decision.

Potential Teen Participants: This form also serves as an assent form. That means that if you choose to take part in this research study, you would sign this form to confirm your choice. Your parent or guardian would also need to give their permission and sign this form for you to join the study.

Parents/Guardians: You have the option of having your child or teen join a research study. This is a parental permission form. It provides a summary of the information the research team will discuss with you. If you decide that your child can take part in this study, you would sign this form to confirm your decision.

When reading this form, please note that the words "you" and "your" refer to the person in the project rather than to a parent or guardian who might sign this form on behalf of the person in the project.

Participants who become adults during the study: Because you are now an adult, you have been asked to read this consent form. After you have read the form, you will be asked to sign it if you still agree to have your samples and information stored and used in a variety of research studies.

The goal of the "Castleman Disease Collaborative Network Biobank" project is to be a patient-driven movement that empowers people with Castleman Disease to directly transform research and treatment of disease by providing blood, sharing part of their stored tissue, and copies of their medical records for

QUORUM REVIEW
APPROVED
INSTITUTIONAL
REVIEW BOARD

use in a variety of research projects. Because we are enrolling people with Castleman across the country regardless of where they are being treated, this study will allow many more people to contribute to research than has previously been possible.

1. What is the purpose of this study?

We want to understand Castleman Disease better so that we can develop more effective therapies. The Castleman Disease Collaborative Network would like to partner directly with people with Castleman Disease, so we can study many more aspects of Castleman Disease than would otherwise be possible.

2. What will I have to do if I agree to participate in this study?

You will answer some questions about your disease and medical care and send a blood sample and/or a saliva sample to us in a pre-stamped package that we will provide. The blood sample should be collected during your regularly scheduled clinical visit if possible. We will take care of obtaining copies of your medical records as well as small amounts of your stored tissues (if available) from the hospitals or centers where you receive your medical care.

3. Do I have to participate in this study?

No. Taking part in this study is voluntary. Even if you decide to participate, you can always change your mind and leave the study.

4. Will I benefit from participating?

While taking part in this study will not improve your own health, the information we collect will aid in research efforts to provide better treatment and prevention options to people with Castleman in the future. We will provide updates about key research discoveries made possible by this biobank on our website, and directly to you via email if you so choose.

5. What are the risks of taking part in this research?

There may be a risk that your information (which includes your genetic information and information from your medical records) could be seen by unauthorized individuals. However, we have procedures and security measures in place designed to minimize this risk and protect the confidentiality of your information.

Investigators do not plan on returning the research test results to you, and the results will not be placed in your regular medical record. However, if a research test reveals something that could potentially affect your care (for example, a possible misdiagnosis), they may contact your physician to have the tests confirmed in a clinical lab. Your physician, not the investigator, will then follow-up with you. We

QUORUM REVIEW
APPROVED
INSTITUTIONAL
REVIEW BOARD

will obtain consent from you to provide your physician your research results and ask you for updated physician contact information at that time.

If you decide to give blood for this biobank, you may experience pain, bruising, dizziness, or an infection.

6. Will it cost me anything to participate in this study?

No.

7. Who will use my samples and see my information?

Your samples and health information will be available to researchers at the Castleman Disease Collaborative Network (CDCN), a not-for-profit global initiative dedicated to accelerating research and treatment for Castleman disease. Your samples may also be used by outside researchers, both in the U.S. and internationally. After removing your name and other readily identifiable information, we will share tissues and information with qualified researchers, who have undergone review and approval by a CDCN Advisory Board. Results from the research studies may be shared with the greater research community via publications, as well as central data banks at the National Institutes of Health.

8. Can I stop taking part in this research study?

Yes, you can withdraw from this research study at any time. CDCN will destroy all your samples in its possession; if samples and information have already been sent to researchers, the CDCN will notify the researcher of the participant's wishes but the CDCN cannot guarantee these samples will be destroyed. In addition, research data may have already been published before you withdrew and that cannot be retracted. Your information would be removed from future studies.

9. What if I have questions?

If you have any questions, please contact either the Principal Investigator, Dr. David Fajgenbaum at (215) 614-0936. In case you want to inquire via writing, their address is 1001 S. 18th St, A Philadelphia PA 19146.

FULL RESEARCH CONSENT FORM

Adult Participants or Parents/Guardians

The Castleman Disease Collaborative Network Biobank: A Collection of Biospecimens and Clinical Data to Facilitate Research

QUORUM REVIEW
APPROVED
INSTITUTIONAL
REVIEW BOARD

A. Introduction

You are being invited to participate in a research study that will establish a Castleman Disease biobank. A biobank, sometimes called a biorepository, collects, stores, analyzes and distributes samples of human tissues, blood, and related health information to qualified scientists. Creation of a biobank for this disease may help doctors and researchers better understand why Castleman Disease occurs and develop ways to better treat and prevent it. Because Castleman Disease is so rare, it has not been well studied, due in part to a lack of available biospecimens and data for research use.

You are being asked to participate in the study because you have Castleman Disease. With your decision to participate, the CDCN will have access to your already existing tissues (blood, bone marrow, and/or lymph node) from your hospital. You also have the ability to provide additional blood or saliva samples.

The biobank is being established by the Castleman Disease Collaborative Network (CDCN), a global initiative dedicated to accelerating research and treatment for Castleman Disease. We work to achieve this by facilitating collaboration among the global research community, mobilizing resources, strategically investing in high-impact research, and supporting people with Castleman Disease and their family members. This biobank is paid for by the CDCN.

This form describes the project in order to help you decide if you want to participate. This form will tell you what you will have to do and the risks and benefits of participating.

If you have any questions about or do not understand something in this form, you should ask the research staff. You should also discuss your participation with anyone you choose in order to better understand this project and your options. Do not sign this form unless the staff has answered your questions and you decide that you want to be part of this project.

Participating in research is not the same as getting regular medical care. The purpose of regular medical care is to improve your health. The purpose of medical research is to advance the understanding and treatment of disease. Participating in this project does not replace your regular medical care.

B. What is the purpose of this biobank?

The Castleman Disease Collaborative Network is creating this biobank to support research. We want scientists to better understand Castleman Disease and work toward developing more effective therapies. We would like to partner directly with people with Castleman, so we can study many more aspects of Castleman than has previously been possible. In addition, because we are enrolling people with Castleman across the country regardless of where they are being treated, this study will allow many more people to directly contribute to research than might otherwise be feasible.

By participating in this biobank project, you will be asked to provide blood or saliva now or in the future, tissue (lymph node, bone marrow samples) left over from past medical tests, and/or medical information to be used for research. We would also like your permission to access and share (after removing your name and other personal identifiers) your medical information along with your specimens.

QUORUM REVIEW
APPROVED
INSTITUTIONAL
REVIEW BOARD

Some of the research might involve studying DNA contained in your samples. DNA is the material that makes up genes. Genes determine things like hair color and eye color, and may play a role in Castleman Disease. Using DNA from your blood or tissue sample, researchers will study your entire genetic sequence, known as your genome, and store this information. Your genomic data and health information will be studied along with information from other participants in this study, and it will be stored for future studies.

Some of the research might involve creating and distributing cell lines from your tissue so that your cells can grow indefinitely to be used in various projects. Cell lines may be useful because of the characteristics of the cells and/or the products they may produce.

Samples may be studied immediately or may be stored for future use by researchers from universities, the government, health-related companies, and other research institutes. Some researchers will be from the United States and some may be from other countries around the world. A Biobank Advisory Board (BAB) composed of physicians and scientists will review research requests to help ensure that tissues are being utilized in the most impactful manner to aid our understanding of Castleman Disease. The BAB will not to attempt to re-identify you. Future researchers must also sign an agreement they will not attempt to re-identify you. Because technology is changing all the time it is not possible to know all of the techniques that researchers may use now or in the future.

We are asking many people to participate in the biobank, both people with Castleman, their family members, and healthy individuals (as a comparison group). We don't know how many people will participate.

C. What other options are there?

Your participation in the biobank project is voluntary. Your alternative is not to participate. The biobank project does not involve treatment for any condition, so your decision to participate or not to participate will not affect your medical care. Your decision not to participate or to stop participating at any time will not involve any penalty to you and will not involve any loss of benefits to which you might otherwise be entitled.

D. What is involved in the biobank project?

With your consent, project staff will collect medical information through a secure questionnaire regarding your health, the doctors you have seen, as well as your medical and family history. Alternatively, a staff member where you receive your care may review your medical records to collect such information. The medical information may include your patient history, response to treatment, pathology (disease) reports, and radiographic (imaging) reports.

The types of samples the Castleman Disease Collaborative Network wants to collect include:

- leftover tissue (such as lymph nodes and/or bone marrow) that have been taken, are scheduled to be taken, or that may be taken in the future, as part of your regular medical care
- blood samples already stored, scheduled to be taken, or that may be taken in the future

QUORUM REVIEW
APPROVED
INSTITUTIONAL
REVIEW BOARD

- saliva

Each type of sample collection is optional. You can decide to participate in each type of sample collection separately, which means you can agree to some, all, or none of the different types of samples. You can indicate your choice at the end of this form.

If you have had surgery in the past and you provide consent, project staff will contact the hospital(s) where you had surgery to get leftover tissue or fluids that may be stored there. If you are scheduled for a medical procedure or surgery for your care, no additional tissue will be taken beyond what is needed for your care. After the medical staff has used your tissue for diagnosis and care, any leftover samples you have agreed to provide will be transferred to the biobank.

If you consent to have blood drawn for the biobank, it may be collected at the same time as other blood tests or during your surgery, if possible. The biobank will send you blood collection kits with tubes and instructions that you can take to your doctor or local laboratory so they can obtain one or more blood samples. The amount of blood collected will be no greater than 50 mL (about 3 tablespoons) over a 2-month period.

We will link the results of the tissue collected with medical information that has been generated during the course of your regular treatment. We are asking your permission to obtain a copy of your medical record from places where you have received care for your disease.

If you give your consent to be re-contacted, biobank staff may contact you periodically to update your medical information or to ask you to provide new samples for research. You may also choose if you would like the Castleman Disease Collaborative Network to notify you about other research studies in the future.

People who work with the sponsor (CDCN) will store your samples in a secure laboratory. Your samples will be stored as long as the sponsor decides to keep them.

E. How long will I be in this research study?

Your direct involvement will be limited initially to the time it takes to obtain your consent and to collect samples and medical information. Because this project involves a biobank, your samples, data, and health information may be used immediately or may be stored indefinitely for future use. If you agree, we may also re-contact you to get updates about your health and medical care, to ask whether you will give additional samples, or to tell you about other studies.

If a participant is a minor when his/her samples are first placed in the biobank, and he/she later becomes an adult, the project staff will contact the participant to seek his/her consent to continue to use the samples in the biobank. At that time, the participant can decide to continue in this biobank project, or ask that the samples be destroyed. If such a participant cannot be contacted, any samples still in the bank will be retained and will continue to be distributed in de-identified form.

If you want to stop being in the biobank project, contact the Principal Investigator or project staff using the contact information provided later in this form. If you decide to withdraw from the project early, the Principal Investigator or project staff may ask you some questions about being in the project to gain

QUORUM REVIEW
APPROVED
INSTITUTIONAL
REVIEW BOARD

feedback on ways in which biobank may be improved. The Principal Investigator or the sponsor can remove you from the project at any time, even if you want to stay in the project.

F. What kind of information could be found in this study and will I be able to see it?

This research is being done to add to our knowledge of Castleman Disease, and is not intended to provide information about your individual health or treatment. Investigators do not plan on returning the research test results to you, and the results will not be placed in your regular medical record. However, if a research test reveals something that could potentially affect your care (such as a possible misdiagnosis) they may contact your physician to have the tests confirmed in a clinical lab. Your physician, not the investigator, will then follow-up with you.

If you choose, the Castleman Disease Collaborative Network can also keep you up to date on the research outcomes of projects using samples or data from this biobank; however not all research projects will use samples from all the donors to the bank, so your own tissue may or may not have been part of a particular research project. You do not need to participate in this biobank to be kept up-to-date on the Castleman Disease Collaborative Network's research efforts; instead, you can sign up online for our email list here at cdcn.org. We regularly update the website.

If you choose, your samples and information may also be used for other central databanks. To allow research collaborations and sharing of data, the National Institutes of Health (NIH) and other research organizations have developed special data (information) repositories that analyze data and collect the results of certain types of genetic studies. These central banks will store your genetic information and provide them to qualified researchers to do more studies. This information will be available for any research question, such as research to understand what causes certain diseases (for example heart disease, cancer, or psychiatric disorders), development of new scientific methods, or the study of where different groups of people may have come from. We are also asking your permission to share your results with these special controlled-access banks. Controlled-access means that only researchers who apply for and get permission to use the information for a specific research project will be able to access the information. Your genomic data and health information will not be labeled with your name or other information that could be used to identify you. Researchers approved to access information in the database will agree not to attempt to identify you. Your information will be sent only with a code number attached. All directly identifiable information, such as your name, will not be shared with data banks or other investigators. Although there may be a small risk of loss of privacy when sharing this information with these banks, we have established procedures to encode your samples and information and protect your data. Although we will do everything we can to protect the privacy of your data, we cannot absolutely guarantee its privacy or predict how genetic information will be used in the future.

We will publish important discoveries found through these studies in the scientific literature so that the entire research community can work together to better understand Castleman Disease. Your individual data will not be published in a way in which you could be readily identified. Abstracts, which are plain language summaries of the published reports, will be available to you and the general public.

G. What are the risks or discomforts of the biobank project?

QUORUM REVIEW
APPROVED
INSTITUTIONAL
REVIEW BOARD

Your regular medical care will not change if you participate in this biobank project. The collection of tissue samples would only take place in the context of your routine care, and therefore does not add any physical risk to you that you would not already be undertaking. If you agree to give an additional blood sample, the medical staff will take your blood by sticking a needle in your arm or they may collect the blood during surgery. The amount of blood that will be collected will not exceed 50 mL (about 3 tablespoons) over a 2-month period. Some problems you might have from this are:

- It may hurt.
- You may get a bruise.
- You may feel dizzy.
- You may get an infection.

There are some risks related to the use of your medical information and specimens. There could be an accidental release of your medical information to people who are not involved in your medical care. An accidental release of your genetic information could be used to identify you and your family members. We have tried to minimize this risk by carefully limiting access to the computers that would house your information and having other strict data security measures in place. There is still a risk someone could get access to the information we have stored about you, including information about a family member (for example, revealing that you or a blood relative carry a genetic disease). Because your DNA information is unique to you, there is a chance that someone could trace it back to you. The risk of this happening is small, but may be greater in the future.

Federal and State laws also provide some protections against discrimination based on genetic information. For example, the Genetic Information Nondiscrimination Act (GINA) makes it illegal for health insurance companies, group health plans, and most employers to discriminate against you based on your genetic information. However, it does not prevent companies that sell life insurance, disability insurance, or long-term care insurance from using genetic information as a reason to deny coverage or set premiums.

H. What are the benefits of the research study?

Research conducted with your information and specimens will not help you directly, but it may help people with Castleman in the future. Research performed with your information and specimens could result in the discovery and development of new drugs, tests or commercial products.

I. Can I stop being in the research study and what are my rights?

You can stop being in the research study at any time. You may choose to leave your samples and/or information in the biobank but request not to be re-contacted. Or, if you want to end all participation, the CDCN will destroy all of your sample identifiers and medical information in its possession; the CDCN will retain your original consent and the documentation of your withdrawal of consent. Any specimens or data that have already been distributed in a de-identified manner cannot be destroyed, and some research data may have already been published. If data has already been distributed to third parties, it

QUORUM REVIEW
APPROVED
INSTITUTIONAL
REVIEW BOARD

cannot be retrieved (as it will have been de-identified). Any specimens or identifiable data you have given can be removed upon request, but any de-identified, already distributed information or specimens cannot be withdrawn.

To withdraw from research, you must notify, in writing, the Principal Investigator (Address: 1001 S. 18th St, A Philadelphia PA 19146). There are three ways you can choose to stop participating in the Biobank:

1. You may choose to leave your data and unused samples in the Biobank while requesting not to be re-contacted in the future.

2. You may request that your personally identifying information be destroyed, but allow some or all of your samples to continue being used anonymously for future research.

3. You can choose to end all participation, in which case all of the unused samples in the Biobank will be destroyed and your medical information will be deleted.

J. Will I be paid to take part in this research study?

You will not get payment for being in this biobank project. The sponsor does not plan to give you any money or payment resulting from any discoveries or products made from this research project.

K. What are the costs?

Participating in the biobank project should not cost you anything beyond your routine, scheduled medical costs. If you decide to donate saliva and blood samples, we recommend you get these drawn during your regularly scheduled clinical appointments. You and your insurance company will already be paying for the costs of the blood draws as a part of your standard medical care. That way there's no additional cost. If you don't have regularly scheduled follow-up but still want to participate, please visit an outpatient clinic to get your blood drawn. You will need to pay for the cost of the blood draw but the CDCN can reimburse you if you fill out a W2 form and provide a copy of the original receipt. If you have a future scheduled biopsy planned, you and your insurance company will already be paying for the costs of that procedure, so there will be no additional cost. Neither you nor your insurance company will be asked to pay for research tests.

L. What happens if I am injured or sick because I took part in this research study?

If injury (physical, psychological, social, financial, or otherwise) occurs as a result of participation in this project, there are no plans for providing compensation to you or your family.

Be aware that your health care payer/insurer might not cover the costs of study-related injuries.

You do not give up any of your legal rights by signing this form.

M. What about confidentiality?

QUORUM REVIEW
APPROVED
INSTITUTIONAL
REVIEW BOARD

Your individual data and health information (without your name, address, or other details that might identify you) will be put in a controlled-access database. This means that only researchers who apply for and get permission to use the information for a specific research project will be able to access the information. Researchers approved to access information in the database will agree not to attempt to identify you. Access to information with your name, such as the consent form, will be restricted to healthcare staff, the Principal Investigator, designated CDCN staff, and approved CDCN contractors. Documents will be kept in a locked cabinet and information in computers will be protected by passwords. In order to protect your private information, only a unique code number, not your name or medical record number, will appear on your samples or on information sent to researchers outside the CDCN. Some people who work for the biobank will be able to find your name from the code so they can possibly have your sample(s) destroyed if you change your mind later. Except when required by law, your personally identifying information will not be disclosed outside of the CDCN.

N. Whom do I contact if I have questions about the research study?

If you have any questions about the study, please contact the research doctor or study staff listed below by email davidfajgenbaum@gmail.com or calling 215-573-8101:

- David Fajgenbaum, MD (Principal Investigator)

If you prefer to do so in writing, please send written correspondence to the address 1001 S. 18th St, A Philadelphia PA 19146.

You can learn more about the Castleman Disease Collaborative Network by visiting our website at www.cdcn.org.

Quorum Review Institutional Review Board (IRB) has reviewed this project. An IRB is a group of people who review research studies to protect the rights and welfare of research participants. If you have questions about your rights as a research participant, if you are not able to resolve your concerns with the Principal Investigator, if you have a complaint, or if you have general questions about what it means to be in a research project, you can call Quorum Review or visit the Quorum Review website at www.quorumreview.com.

Quorum Review is located in Seattle, Washington.

Office hours are 8:00 AM to 5:00 PM Pacific Time, Monday through Friday.

Ask to speak with a Research Participant Liaison at 888-776-9115 (toll free).

O. Conflict of Interest

Dr. David Fajgenbaum, the person responsible for the conduct of this research study also serves as the Executive Director of the CDCN. You should be aware that you are not under any obligation to participate in this research study.

QUORUM REVIEW
APPROVED
INSTITUTIONAL
REVIEW BOARD

P. Authorization to use your health information for research purposes

Because information about you and your health is personal and private, it generally cannot be used in this research study without your written authorization. Federal law called HIPAA requires that your health care providers and healthcare institutions (hospitals, clinics, doctor's offices) protect the privacy of information that identifies you and relates to your past, present, and future physical and mental health conditions.

If you agree to take part in this research, we will request that your health care providers and healthcare institutions disclose your protected health information to the Castleman Disease Collaborative Network for use in this research study. This may mean that you need to sign an additional form specifically authorizing release to the Network, but this will depend on your doctor's local practices, and we will work with you on this.

This section informs you about how your health information will be used or disclosed in the study. Your information will only be used in accordance with what is described here and as required or allowed by law. If you do not want to allow the uses described here, you cannot be in this biobank. You can still be in the biobank even if you do not give permission for the use and sharing of your information for the optional parts of the biobank.

1. What personal information about me will be used or shared with others during this research?

- Your medical records
- Your tissue samples relevant to this research study and related records
- New health information created from study-related tests and/or questionnaires

2. Why will protected information about me be used or shared with others?

The main reasons include the following:

- To conduct and oversee the research described earlier in this form;
- To ensure the research meets legal, institutional, and accreditation requirements;
- To conduct public health activities (including reporting of adverse events or situations where you or others may be at risk of harm)

3. Who will use or share protected health information about me?

With your consent, biobank staff will obtain information about you either directly from you or by having healthcare staff review your medical records. They may share your consent information in connection with the research study.

QUORUM REVIEW
APPROVED
INSTITUTIONAL
REVIEW BOARD

4. With whom outside of the Castleman Disease Collaborative Network may my personal health information be shared?

While all reasonable efforts will be made to protect the confidentiality of your protected health information, it may also be shared with the following entities:

- Federal and state agencies (for example, the Department of Health and Human Services, the Food and Drug Administration, the National Institutes of Health, and/or the Office for Human Research Protections), or other domestic or foreign government bodies if required by law and/or necessary for oversight purposes. A qualified representative of the FDA, the National Cancer Institute, or Quorum Review may review your medical records.
- Outside individuals or entities that have a need to access this information to perform functions relating to the conduct of this research such as data storage companies.

5. For how long will protected health information about me be used or shared with others?

There is no scheduled date at which your protected health information that is being used or shared for this research will be destroyed, because research is an ongoing process.

6. Statement of privacy rights:

- You have the right to withdraw your permission for the doctors and researchers to use or share your protected health information. We will not be able to withdraw all the information that already has been used or shared with others to carry out related activities such as oversight, or that is needed to ensure quality of the study. To withdraw your permission, you must do so in writing by contacting the researcher listed above in the section: “Whom do I contact if I have questions about the research study?” If you do so, you will no longer be a participant in this study. You can withdraw your permission for the optional parts of the biobank and still be in the biobank.
- After your information is shared with the Network, federal law will no longer require it be protected. The Network may also share your information with other people who the federal law does not apply too. The Network will nonetheless only use or disclose your information as described in this form and will have its own security measures in place to ensure your information and privacy remains protected.

Q. Participation Information

If you decide to sign this consent form, we will ask you for information about contacting your physicians and the hospitals that you were treated at for Castleman. We will not disclose details about the results of your participation in this study with any of the individuals that we contact, but rather ask them to provide us with your medical history and your tissue samples.

QUORUM REVIEW
APPROVED
INSTITUTIONAL
REVIEW BOARD

R. Consent

I have read this form, and I have had the opportunity ask questions about this study. I understand that my participation is voluntary, and if I decide not to participate it will have no impact on my medical care. If decide to participate now, I can decide to stop being in the study at any time. I voluntarily agree to take part in this research biobank. By signing this form, I do not give up any of my legal rights.

I Consent

X Yes I Consent

REQUIRED ACTIVITIES:

I agree to the following activities as part of this biobank. (You must agree to all of the following to participate in the biobank. If you do not agree to all of these, you should not join the biobank.)

You can request my stored tissue samples (blood and/or tissue (lymph nodes, bone marrow)) from my doctors and hospitals. You can perform (or collaborate with others to perform) research on the samples, and store the samples until this research study is complete.

_ Yes I Agree

You can request my tissue samples (blood and/or tissue (lymph nodes, bone marrow)) from my currently scheduled medical procedures from my doctor and hospital. You can perform (or collaborate with others to perform) research on the samples, and store the samples until this research study is complete.

_ Yes I Agree

I am willing to provide leftovers from any future tissue samples that may be taken for my regular medical care (blood and/or tissue (lymph nodes, bone marrow)) to the biobank. I will allow research to be performed on the samples, and the samples to be stored until this research study is complete. (Even if you check this now, you can change your mind in the future.)

_ Yes I Agree

You can request my medical records from my doctors and the hospitals and other places where I received and/or continue to receive my treatment. You can link results of the research you perform on my samples with my medical information from my medical records.

_ Yes I Agree

I am willing to be re-contacted by study staff later to update my medical information. Even if I agree now by checking this box, I can decide not to provide this information when I am re-contacted.

QUORUM REVIEW
APPROVED
INSTITUTIONAL
REVIEW BOARD

_ Yes I Agree

You can use the results of the research on my samples and my medical information for future research studie**s**, including studies that have not yet been designed, studies for diseases other than Castleman Disease, and/or studies that may be for commercial purposes.

_ Yes I Agree

You can share the results of the research on my samples and my medical information with central data banks (e.g., the NIH) and with other qualified researchers. It will be shared in a manner that does not include my name, social security number, or any other information that could be used to readily identify me. It can be used by other qualified researchers to perform future research studies, including studies that have not yet been designed, studies for diseases other than Castleman Disease, and studies that may be for commercial purposes.

_ Yes I Agree

OPTIONAL ACTIVITIES:

Please check below if you are willing to allow any of these optional activities as part of this research biobank. You can still be in the study even if you do not agree to any of these activities:

I am willing to have blood drawn for the biobank. You can perform (or collaborate with others to perform) research on the blood sample that I will send you and store the sample until this research study is complete.

_ Yes

I am willing to have my saliva collected for the biobank. You can perform (or collaborate with others to perform) research on the saliva sample that I will send you and store the sample until this research study is complete.

_ Yes

I am willing to be re-contacted by study staff later to provide new samples for research. Even if I agree now by checking this box, I can decide not to provide this information or additional samples when I am re-contacted.

_ Yes

You may re-contact me to notify me about clinical trials and other research opportunities.

_ Yes

QUORUM REVIEW
APPROVED
INSTITUTIONAL
REVIEW BOARD

I am willing to be re-contacted to obtain my consent in the event that return of my personal research results becomes possible.

_ Yes

You should print a copy of this form for your records.

Signature of Parent or Legal Guardian

First

Last

Date of Parent or Legal Guardian Signature

MM/DD/YYYY

Relationship of Parent/Guardian to Participant

Research Assent Form

Ages 7 to 13

Study Title: The Castleman Disease Collaborative Network Biobank: A Collection of Biospecimens and Clinical Data to Facilitate Research
Study #: 001
Sponsor: Castleman Disease Collaborative Network
Study Investigator: David Fajgenbaum
Castleman Disease Collaborative Network
1001 S. 18th St., Unit A., Philadelphia, PA 19146
Telephone Number: (610) 304-0696

What is a research study?

QUORUM REVIEW
APPROVED
INSTITUTIONAL
REVIEW BOARD

Research studies help us learn new things. We can test new ideas. First, we ask a question. Then we try to find the answer.

This form talks about our research and the choice that you have to take part in it. We want you to ask us any questions that you have. You can ask questions any time.

Important things to know…

- You get to decide if you want to take part.
- You can say ‘No’ or you can say ‘Yes’.
- No one will be upset if you say ‘No’.
- If you say ‘Yes’, you can always say ‘No’ later.
- You can say ‘No’ at anytime.
- Your doctors will still take good care of you no matter what you decide.
- You can ask the doctor or nurse questions before you make up your mind. You can also talk to your mom or dad or anyone you want to about the study.
- You can ask to read the information the doctor gives your mom or dad about this study.

Why are we doing this research?

We are doing this research to find out more about Castleman Disease.

What would happen if I join this research?

If you decide to be in the research, we would ask you to do the following:

- Blood draws: You may need a needle poke so we could test some of your blood. If possible, we will try to get blood without a new poke.
- Saliva sample: you may give some saliva (spit) for tests.
- We might ask you for leftovers of other types of samples. The doctor may get these from any special tests or operations you might have as part of your regular care.
- Questions: We would ask you to read questions, then you would mark your answers on the paper.
- Medical Records: You would allow for medical records to be shared with the research team.

QUORUM REVIEW
APPROVED
INSTITUTIONAL
REVIEW BOARD

- Your samples and information will be stored for a long time. The could be used in lots of different research studies about Castleman or other diseases.
- Some parts of the study are optional. With your parent/guardian, you will decide your level of participation.

Could bad things happen if I join this research?

Some of the tests might make you uncomfortable or the questions might be hard to answer. We will try to make sure that no bad things happen.

The poke to test your blood can hurt. Sometimes the needle can leave a bruise on the skin.

You can say ‘no’ to what we ask you to do for the research at any time and we will stop.

Could the research help me?

This research will not help you. We do hope to learn something from this research though. And someday we hope it will help other kids who have Castleman Disease like you do.

What else should I know about this research?

If you don’t want to be in the study, you don’t have to be.

It is also OK to say yes and change your mind later. You can stop being in the research at any time. If you want to stop, please tell the research doctors.

You will not be paid to be in the study.

You can ask questions any time. You can talk to David Fajgenbaum. His phone number is (215)-614-0936. Ask us any questions you have. If you prefer to do so in writing, Dr. Fajgenbaum’s address is 1001 S. 18th A Philadelphia, PA 19146. Take the time you need to make your choice.

If you want to ask questions about what it means to be in a research study, you or your mom or dad can call Quorum Review at 1-888-776-9115 (toll free).

QUORUM REVIEW
APPROVED
INSTITUTIONAL
REVIEW BOARD

Is there anything else?

If you want to be in the research after we talk, please write your name below. This shows we talked about the research and that you want to take part.

If you have any questions, please contact Dustin Shilling at 1-215-614-0689 or dustin@castlemannetwork.org

Printed Name of Child

First

Last

Signature of Parent/Legal Guardian

First

Last

Date of Parent/Legal Guardian Signature

MM/DD/YYYY

You should print a copy of this form for your records.

--NEXT PAGE--

If you selected "Parent of pediatric CD patient," at the beginning of this survey please respond on behalf of the PATIENT.

If you selected "Adult CD patient"/ "Related family member of CD patient"/ "Patient with related disease"/ "Healthy volunteer," at the beginning of this survey, please enter YOUR information.

Please provide your current legal first name.

QUORUM REVIEW
APPROVED
INSTITUTIONAL
REVIEW BOARD

Please provide your current legal middle name. Please type in 0 if none.

Please provide your current legal last name.

Has your name changed since birth?

_ Yes

_ No

What is your date of birth?

MM/DD/YYYY

What was your physical sex at birth?

_ Male

_ Female

What city/municipality were you born in?

What country were you born in?

Please provide your current full mailing address below.

Street Address

Address Line 2

City

State / Province / Region

QUORUM REVIEW
APPROVED
INSTITUTIONAL
REVIEW BOARD

Postal / Zip Code

Country

_ Which of the following selections describes your ancestry? Please select all that apply.

_ American Indian or Alaska Native

_ Asian

_ Black or African American

_ Hispanic or Latino

_ Native Hawaiian or Other Pacific Islander

_ Caucasian, Non-Finnish Ancestry

_ Caucasian, Finnish Ancestry

_ I prefer not to say

_ Other (please specify)

Please enter your primary phone number where we may contact you if needed.

Please enter your email address.

--NEXT PAGE—

If you selected "Parent of pediatric CD patient," at the beginning of this survey please respond on behalf of the PATIENT.

If you selected "Adult CD patient"/ "Related family member of CD patient"/ "Patient with related disease"/ "Healthy volunteer," at the beginning of this survey, please enter YOUR information.

Are you interested in donating a blood sample?

_ Yes

_ No

QUORUM REVIEW
APPROVED
INSTITUTIONAL
REVIEW BOARD

Are you interested in having your existing tissue block (lymph node and/or bone marrow) transferred?

_ Yes

_ No

If you answered "yes" to either of the previous questions, could you please provide the name and city/state of all of the hospitals where you may have samples stored (Please list all hospitals even if you are unsure if samples remain)?

--NEXT PAGE—

This is the next section of the CDCN Survey.

Please select "Next Page" to continue with the survey. Depending on your prior response, certain pages will not apply to you and you will automatically bypass them.

--NEXT PAGE—

If you selected "Parent of pediatric CD patient," at the beginning of this survey please respond on behalf of the PATIENT.

If you selected "Adult CD patient"/ "Related family member of CD patient"/ "Patient with related disease"/ "Healthy volunteer," at the beginning of this survey, please enter YOUR information.

Please list full name of all physicians and institutions.

What is the name of your current Castleman disease treating physician(s) and their corresponding institution(s)?

QUORUM REVIEW
APPROVED
INSTITUTIONAL
REVIEW BOARD

Please list any other treating physician(s) and their corresponding institution(s) that you have seen since you Castleman disease symptoms started?

Have you had a lymph node biopsy pathology report state "consistent with", "suggestive of", "compatible with", or "diagnostic of" Castleman disease?

_ Yes

_ No

Has your clinician diagnosed you with unicentric (enlargement of a single region of lymph nodes) or multicentric (enlargement of more than one region of lymph nodes) Castleman disease?

_ Unicentric

_ Multicentric

_ Unsure

What date were you diagnosed?

MM/DD/YYYY

Have you been told that you have or may have TAFRO syndrome?

_ Yes

_ No

_ Unsure

Have you ever been diagnosed with HIV?

_ Yes

_ No

_ I do not feel comfortable answering this question (prefer not to answer)

QUORUM REVIEW
APPROVED
INSTITUTIONAL
REVIEW BOARD

Has your clinician told you if your disease is HHV-8 positive, HHV-8-negative, or do you not know?

_ HHV-8-positive

_ HHV-8-negative

_ Do Not Know

If you had a HHV-8 test done, which test was performed to determine your HHV-8 status?

_ LANA 1

_ PCR of blood

_ PCR of Lymph node

_ Unsure

Which of the following clinical features have you experienced during active disease/flare? Please select "None" if you did not experience any.

_ None

Clinical features experienced during active disease/flare:

_ Flu-like symptoms

_ Night sweats

_ Fevers (>100.5F)

_ Weight loss

_ Fatigue

_ Large spleen and/or liver

_ Edema/anasarca, effusions, ascites, or other form of fluid overload

_ Eruption of cherry hemangiomas on the skin or violaceous papules

_ Lymphocytic Interstitial pneumonitis

Do you have any other symptoms believed to be related to Castleman disease? If so, please list these symptoms. Also, please comment on the severity of these symptoms and/or the above symptoms. Please type in NA if this does not apply to you.

QUORUM REVIEW
APPROVED
INSTITUTIONAL
REVIEW BOARD

Which of the following laboratory abnormalities have you experienced during active disease/flare? Please select "None" if you did not experience any.

_ None

Laboratory abnormalities experienced during active disease/flare: *

_ Elevated C-Reactive Protein (CRP) (>10mg/dL)

_ Elevated Erythrocyte Sedimentation Rate (ESR) (>15mm/hr)

_ Low Hemoglobin/Anemia (Hgc<12.5 for males, <11.5 for females)

_ Elevated Platelets/Thrombocytosis (>400k/uL)

_ Low Platelets/Thrombocytopenia (<150K/uL)

_ Low Albumin/Hypoalbuminemia (<3.5 gm/dL)

_ Kidney dysfunction (elevated Creatinine or BUN) or proteinuria (protein in urine)

_ High Gamma Globin

_ Levels/Hypergammaglobulinemia (>1700mg/dL)

Do you have any other laboratory abnormalities believed to be related to Castleman disease? If so, please list these symptoms. Also, please comment on the severity of these laboratory abnormalities and/or the above laboratory abnormalities. Please type in NA if this does not apply to you.

Did you have a bone marrow biopsy during active disease/flare? Please specify if the pathology report stated fibrosis, myelofibrosis, or reticulin fibrosis.

_ Yes, stated fibrosis/myelofibrosis/reticulin fibrosis

_ Yes, did not specify

_ No, did not have bone marrow biopsy done

_ Unsure

Have you ever been hospitalized for Castleman disease or associated complications? If so, for how long?

QUORUM REVIEW
APPROVED
INSTITUTIONAL
REVIEW BOARD

_ Never been hospitalized

_ Less than 3 days

_ 3 – 6 days

_ 1 – 4 weeks

_ More than 4 weeks

Please select any disorders listed below that you have been diagnosed with or select "Other" if you have been diagnosed with a disease not on this list.

_ Clinical Epstein Barr Virus (EBV)

_ lymphoproliferative disorders

_ Cytomegalovirus (CMV)

_ Toxoplasmosis

_ Active Tuberculosis

_ Systemic lupus erythematosus

_ Rheumatoid arthritis

_ Adult-onset Still's disease

_ Juvenile Idiopathic Arthritis

_ IgG4-related disease

_ Hodgkin's lymphoma

_ Non-Hodgkin's lymphoma

_ Multiple myeloma

_ Primary lymph node plasmacytoma

_ Follicular dendritic cell sarcoma

_ POEMS syndrome

_ Other (Please Specify)

If you selected "Other", please specify the disease(s).

Please supply any reports or medical records associated with those diagnoses if you have any.

QUORUM REVIEW
APPROVED
INSTITUTIONAL
REVIEW BOARD

[FILE UPLOAD BUTTON]

--NEXT PAGE—

If you selected "Parent of pediatric CD patient," at the beginning of this survey please respond on behalf of the PATIENT.

If you selected "Adult CD patient"/ "Related family member of CD patient"/ "Patient with related disease"/ "Healthy volunteer," at the beginning of this survey, please enter YOUR information.

What was the first treatment regimen you received for your Castleman disease (CD)? (Examples of treatment regimen include Prednisone, Siltuximab, Siltuximab+ Prednisone, CHOP, Tocilizumab, Tocilizumab+Prednisone, Surgery, Radiation, Stem Cell Transplant, etc.)

What was the date you began your first treatment regimen?

MM/DD/YYYY

Is this first treatment regimen still ongoing?

_ Yes

X No

What is the date you discontinued this treatment regimen?

MM/DD/YYYY

Do you believe that your symptoms/laboratory values improved on treatment completely, partially, or not at all as a result of your first treatment regimen?

_ Complete improvement in Castleman disease symptoms

X Partial improvement in Castleman disease symptoms

_ No improvement in Castleman disease symptoms

If you improved on your first treatment regimen, did you ever experience progression of the disease at a later time?

QUORUM REVIEW
APPROVED
INSTITUTIONAL
REVIEW BOARD

_ Yes

_ No

What was the date that your disease progressed?

MM/DD/YYYY

Have you received a second treatment regimen?

X Yes

_ No

What was the second treatment regimen you received for your Castleman disease (CD)? (Examples of treatment regimen include Prednisone, Siltuximab, Siltuximab+ Prednisone, CHOP, Tocilizumab, Tocilizumab+Prednisone, Surgery, Radiation, Stem Cell Transplant, etc.)

What was the date you began your second treatment regimen?

MM/DD/YYYY

Is this second treatment regimen still ongoing?

X Yes

_ No

What is the date you discontinued this treatment regimen?

MM/DD/YYYY

Do you believe that your symptoms/laboratory values improved on treatment completely, partially, or not at all as a result of your first treatment regimen?

_ Complete improvement in Castleman disease symptoms

X Partial improvement in Castleman disease symptoms

_ No improvement in Castleman disease symptoms

QUORUM REVIEW
APPROVED
INSTITUTIONAL
REVIEW BOARD

If you improved on your second treatment regimen, did you ever experience progression of the disease at a later time?

X Yes

_ No

What was the date that your disease progressed?

MM/DD/YYYY

Have you received a third treatment regimen?

_ Yes

X No

--NEXT PAGE—

If you selected "Parent of pediatric CD patient," at the beginning of this survey please respond on behalf of the PATIENT.

If you selected "Adult CD patient"/ "Related family member of CD patient"/ "Patient with related disease"/ "Healthy volunteer," at the beginning of this survey, please enter YOUR information.

When was the last time that you had blood work done?

MM/DD/YYYY

When was the last time that you visited your physician?

MM/DD/YYYY

Please upload a copy of the results of your most recent blood work or clinical note here if you have a copy

[FILE UPLOAD BUTTON]

QUORUM REVIEW
APPROVED
INSTITUTIONAL
REVIEW BOARD

At time of this blood draw, do you have active disease/flare (e.g. presence of symptoms related to your Castleman disease)?

X Yes

_ No

At what stage of the disease do you feel you are in currently?

_ Early stages of disease activity

_ At the peak of disease activity

_ Improving since worst disease activity

When was the last time you experienced active disease/flare (presence of symptoms related to your Castleman disease)?

MM/DD/YYYY

Which of the following clinical features are you currently experiencing? Please select "None" if you have not experienced any or select the items from the corresponding list.

_ None

Clinical features "Currently" experienced:

_ Flu-like symptoms

_ Night sweats

_ Fevers (>100.5F)

_ Weight loss

_ Fatigue

_ Large spleen and/or liver

_ Edema/anasarca, effusions, ascites, or other form of fluid overload

_ Eruption of cherry hemangiomas on the skin or violaceous papules

_ Lymphocytic Interstitial pneumonitis

QUORUM REVIEW
APPROVED
INSTITUTIONAL
REVIEW BOARD

Which of the following laboratory abnormalities did you experience when your blood was last drawn? Please select "None" if you have not experienced any or select the items from the corresponding list.

_ None

Laboratory abnormalities experience when your blood was last drawn:

_ Elevated C-Reactive Protein (CRP) (>10mg/dL)

_ Elevated Erythrocyte Sedimentation Rate (ESR) (>15mm/hr)

_ Low Hemoglobin/Anemia (Hgc<12.5 for males, <11.5 for females)

_ Elevated Platelets/Thrombocytosis (>400k/uL)

_ Low Platelets/Thrombocytopenia (<150K/uL)

_ Low Albumin/Hypoalbuminemia (<3.5 gm/dL)

_ Kidney dysfunction (elevated Creatinine or BUN) or proteinuria (protein in urine)

_ High Gamma Globin

_ Levels/Hypergammaglobulinemia (>1700mg/dL)

Are you currently registered in the ACCELERATE registry? (Please visit http://www.cdcn.org/accelerate to get more details if you are interested in joining.)

_ Yes

_ No

Are you currently on any medications (for Castleman disease or other conditions)?

X Yes

_ No

Please list any medications that you are currently taking.

--NEXT PAGE—

QUORUM REVIEW
APPROVED
INSTITUTIONAL
REVIEW BOARD

Thank you for completing our survey! We really appreciate your support in fighting back against Castleman disease.

The CDCN will review the information you have provided and determine how you can be involved in providing samples for research. We will be in touch once we have gathered enough information to be able to send you a kit in the mail with empty tubes or if we have any questions for you.

QUORUM REVIEW
APPROVED
INSTITUTIONAL
REVIEW BOARD

Castleman Patients > 18 (adults)

Thank you for filling out this Survey, so that we will be able to include your samples in the BioBank.

If you are presented with a Survey Page that does not contain any questions, please select "Next Page" to continue through the Survey. That particular Survey Page does not contain any questions that pertain to you based on your prior responses.

If you have any questions, please contact Dustin Shilling at 1-215-614-0689
or dustin@castlemannetwork.org

The following form will enable you to participate in the CDCN Biobank (Castlebank). Tissue samples such as blood or excess biopsy samples from patients will enable scientists to better characterize and understand Castleman disease and develop better treatments. Any blood or tissue donation will be used for high-impact research to battle this disease!

Is this your first time filling out this Survey? (If you do not remember if you have participated in the past, please call 1-215-614-0689)

X First Time Participant

_ Repeat Participant

--NEXT PAGE--

Please select the appropriate category that describes the PERSON completing this survey. (This question refers to the person completing the survey, NOT necessarily the patient)

X Adult CD patient (> 18 years old)

_ Parent of pediatric CD patient

_ Related family member of CD patient (without CD and > 18 and NOT parent of pediatric CD patient)

_ Patient with related disease (e.g. Lupus, Rheumatoid Arthritis and > 18 years old)

_ Healthy volunteer (> 18 years old)

RESEARCH CONSENT/ASSENT FORM – KEY POINTS

For Adult Participants or Parents/Guardians

For Minors Age 14 and Older

QUORUM REVIEW
APPROVED
INSTITUTIONAL
REVIEW BOARD

For Participants Who Become Adults While in the Study

Study Title: The Castleman Disease Collaborative Network Biobank: A Collection of Biospecimens and Clinical Data to Facilitate Research
Study #: 001
Sponsor: Castleman Disease Collaborative Network
Study Investigator: David Fajgenbaum
Castleman Disease Collaborative Network
1001 S. 18th St., Unit A., Philadelphia, PA 19146
Telephone Number: (610) 304-0696

Potential Participants 18 years and older: This is a consent form. It provides a summary of the information the research team will discuss with you. If you decide that you would like to take part in this research study, you would sign this form to confirm your decision.

Potential Teen Participants: This form also serves as an assent form. That means that if you choose to take part in this research study, you would sign this form to confirm your choice. Your parent or guardian would also need to give their permission and sign this form for you to join the study.

Parents/Guardians: You have the option of having your child or teen join a research study. This is a parental permission form. It provides a summary of the information the research team will discuss with you. If you decide that your child can take part in this study, you would sign this form to confirm your decision.

When reading this form, please note that the words “you” and “your” refer to the person in the project rather than to a parent or guardian who might sign this form on behalf of the person in the project.

Participants who become adults during the study: Because you are now an adult, you have been asked to read this consent form. After you have read the form, you will be asked to sign it if you still agree to have your samples and information stored and used in a variety of research studies.

The goal of the “Castleman Disease Collaborative Network Biobank” project is to be a patient-driven movement that empowers people with Castleman Disease to directly transform research and treatment of disease by providing blood, sharing part of their stored tissue, and copies of their medical records for use in a variety of research projects. Because we are enrolling people with Castleman across the country regardless of where they are being treated, this study will allow many more people to contribute to research than has previously been possible.

1. What is the purpose of this study?

QUORUM REVIEW
APPROVED
INSTITUTIONAL
REVIEW BOARD

We want to understand Castleman Disease better so that we can develop more effective therapies. The Castleman Disease Collaborative Network would like to partner directly with people with Castleman Disease, so we can study many more aspects of Castleman Disease than would otherwise be possible.

2. What will I have to do if I agree to participate in this study?

You will answer some questions about your disease and medical care and send a blood sample and/or a saliva sample to us in a pre-stamped package that we will provide. The blood sample should be collected during your regularly scheduled clinical visit if possible. We will take care of obtaining copies of your medical records as well as small amounts of your stored tissues (if available) from the hospitals or centers where you receive your medical care.

3. Do I have to participate in this study?

No. Taking part in this study is voluntary. Even if you decide to participate, you can always change your mind and leave the study.

4. Will I benefit from participating?

While taking part in this study will not improve your own health, the information we collect will aid in research efforts to provide better treatment and prevention options to people with Castleman in the future. We will provide updates about key research discoveries made possible by this biobank on our website, and directly to you via email if you so choose.

5. What are the risks of taking part in this research?

There may be a risk that your information (which includes your genetic information and information from your medical records) could be seen by unauthorized individuals. However, we have procedures and security measures in place designed to minimize this risk and protect the confidentiality of your information.

Investigators do not plan on returning the research test results to you, and the results will not be placed in your regular medical record. However, if a research test reveals something that could potentially affect your care (for example, a possible misdiagnosis), they may contact your physician to have the tests confirmed in a clinical lab. Your physician, not the investigator, will then follow-up with you. We will obtain consent from you to provide your physician your research results and ask you for updated physician contact information at that time.

If you decide to give blood for this biobank, you may experience pain, bruising, dizziness, or an infection.

6. Will it cost me anything to participate in this study?

QUORUM REVIEW
APPROVED
INSTITUTIONAL
REVIEW BOARD

No.

7. Who will use my samples and see my information?

Your samples and health information will be available to researchers at the Castleman Disease Collaborative Network (CDCN), a not-for-profit global initiative dedicated to accelerating research and treatment for Castleman disease. Your samples may also be used by outside researchers, both in the U.S. and internationally. After removing your name and other readily identifiable information, we will share tissues and information with qualified researchers, who have undergone review and approval by a CDCN Advisory Board. Results from the research studies may be shared with the greater research community via publications, as well as central data banks at the National Institutes of Health.

8. Can I stop taking part in this research study?

Yes, you can withdraw from this research study at any time. CDCN will destroy all your samples in its possession; if samples and information have already been sent to researchers, the CDCN will notify the researcher of the participant's wishes but the CDCN cannot guarantee these samples will be destroyed. In addition, research data may have already been published before you withdrew and that cannot be retracted. Your information would be removed from future studies.

9. What if I have questions?

If you have any questions, please contact either the Principal Investigator, Dr. David Fajgenbaum at (215) 614-0936. In case you want to inquire via writing, their address is 1001 S. 18th St, A Philadelphia PA 19146.

FULL RESEARCH CONSENT FORM

Adult Participants or Parents/Guardians

The Castleman Disease Collaborative Network Biobank: A Collection of Biospecimens and Clinical Data to Facilitate Research

A. Introduction

You are being invited to participate in a research study that will establish a Castleman Disease biobank. A biobank, sometimes called a biorepository, collects, stores, analyzes and distributes samples of human tissues, blood, and related health information to qualified scientists. Creation of a biobank for this disease may help doctors and researchers better understand why Castleman Disease occurs and develop

QUORUM REVIEW
APPROVED
INSTITUTIONAL
REVIEW BOARD

ways to better treat and prevent it. Because Castleman Disease is so rare, it has not been well studied, due in part to a lack of available biospecimens and data for research use.

You are being asked to participate in the study because you have Castleman Disease. With your decision to participate, the CDCN will have access to your already existing tissues (blood, bone marrow, and/or lymph node) from your hospital. You also have the ability to provide additional blood or saliva samples.

The biobank is being established by the Castleman Disease Collaborative Network (CDCN), a global initiative dedicated to accelerating research and treatment for Castleman Disease. We work to achieve this by facilitating collaboration among the global research community, mobilizing resources, strategically investing in high-impact research, and supporting people with Castleman Disease and their family members. This biobank is paid for by the CDCN.

This form describes the project in order to help you decide if you want to participate. This form will tell you what you will have to do and the risks and benefits of participating.

If you have any questions about or do not understand something in this form, you should ask the research staff. You should also discuss your participation with anyone you choose in order to better understand this project and your options. Do not sign this form unless the staff has answered your questions and you decide that you want to be part of this project.

Participating in research is not the same as getting regular medical care. The purpose of regular medical care is to improve your health. The purpose of medical research is to advance the understanding and treatment of disease. Participating in this project does not replace your regular medical care.

B. What is the purpose of this biobank?

The Castleman Disease Collaborative Network is creating this biobank to support research. We want scientists to better understand Castleman Disease and work toward developing more effective therapies. We would like to partner directly with people with Castleman, so we can study many more aspects of Castleman than has previously been possible. In addition, because we are enrolling people with Castleman across the country regardless of where they are being treated, this study will allow many more people to directly contribute to research than might otherwise be feasible.

By participating in this biobank project, you will be asked to provide blood or saliva now or in the future, tissue (lymph node, bone marrow samples) left over from past medical tests, and/or medical information to be used for research. We would also like your permission to access and share (after removing your name and other personal identifiers) your medical information along with your specimens.

Some of the research might involve studying DNA contained in your samples. DNA is the material that makes up genes. Genes determine things like hair color and eye color, and may play a role in Castleman Disease. Using DNA from your blood or tissue sample, researchers will study your entire genetic sequence, known as your genome, and store this information. Your genomic data and health information will be studied along with information from other participants in this study, and it will be stored for future studies.

QUORUM REVIEW
APPROVED
INSTITUTIONAL
REVIEW BOARD

Some of the research might involve creating and distributing cell lines from your tissue so that your cells can grow indefinitely to be used in various projects. Cell lines may be useful because of the characteristics of the cells and/or the products they may produce.

Samples may be studied immediately or may be stored for future use by researchers from universities, the government, health-related companies, and other research institutes. Some researchers will be from the United States and some may be from other countries around the world. A Biobank Advisory Board (BAB) composed of physicians and scientists will review research requests to help ensure that tissues are being utilized in the most impactful manner to aid our understanding of Castleman Disease. The BAB will not to attempt to re-identify you. Future researchers must also sign an agreement they will not attempt to re-identify you. Because technology is changing all the time it is not possible to know all of the techniques that researchers may use now or in the future.

We are asking many people to participate in the biobank, both people with Castleman, their family members, and healthy individuals (as a comparison group). We don't know how many people will participate.

C. What other options are there?

Your participation in the biobank project is voluntary. Your alternative is not to participate. The biobank project does not involve treatment for any condition, so your decision to participate or not to participate will not affect your medical care. Your decision not to participate or to stop participating at any time will not involve any penalty to you and will not involve any loss of benefits to which you might otherwise be entitled.

D. What is involved in the biobank project?

With your consent, project staff will collect medical information through a secure questionnaire regarding your health, the doctors you have seen, as well as your medical and family history. Alternatively, a staff member where you receive your care may review your medical records to collect such information. The medical information may include your patient history, response to treatment, pathology (disease) reports, and radiographic (imaging) reports.

The types of samples the Castleman Disease Collaborative Network wants to collect include:

- leftover tissue (such as lymph nodes and/or bone marrow) that have been taken, are scheduled to be taken, or that may be taken in the future, as part of your regular medical care
- blood samples already stored, scheduled to be taken, or that may be taken in the future
- saliva

Each type of sample collection is optional. You can decide to participate in each type of sample collection separately, which means you can agree to some, all, or none of the different types of samples. You can indicate your choice at the end of this form.

QUORUM REVIEW
APPROVED
INSTITUTIONAL
REVIEW BOARD

If you have had surgery in the past and you provide consent, project staff will contact the hospital(s) where you had surgery to get leftover tissue or fluids that may be stored there. If you are scheduled for a medical procedure or surgery for your care, no additional tissue will be taken beyond what is needed for your care. After the medical staff has used your tissue for diagnosis and care, any leftover samples you have agreed to provide will be transferred to the biobank.

If you consent to have blood drawn for the biobank, it may be collected at the same time as other blood tests or during your surgery, if possible. The biobank will send you blood collection kits with tubes and instructions that you can take to your doctor or local laboratory so they can obtain one or more blood samples. The amount of blood collected will be no greater than 50 mL (about 3 tablespoons) over a 2-month period.

We will link the results of the tissue collected with medical information that has been generated during the course of your regular treatment. We are asking your permission to obtain a copy of your medical record from places where you have received care for your disease.

If you give your consent to be re-contacted, biobank staff may contact you periodically to update your medical information or to ask you to provide new samples for research. You may also choose if you would like the Castleman Disease Collaborative Network to notify you about other research studies in the future.

People who work with the sponsor (CDCN) will store your samples in a secure laboratory. Your samples will be stored as long as the sponsor decides to keep them.

E. How long will I be in this research study?

Your direct involvement will be limited initially to the time it takes to obtain your consent and to collect samples and medical information. Because this project involves a biobank, your samples, data, and health information may be used immediately or may be stored indefinitely for future use. If you agree, we may also re-contact you to get updates about your health and medical care, to ask whether you will give additional samples, or to tell you about other studies.

If a participant is a minor when his/her samples are first placed in the biobank, and he/she later becomes an adult, the project staff will contact the participant to seek his/her consent to continue to use the samples in the biobank. At that time, the participant can decide to continue in this biobank project, or ask that the samples be destroyed. If such a participant cannot be contacted, any samples still in the bank will be retained and will continue to be distributed in de-identified form.

If you want to stop being in the biobank project, contact the Principal Investigator or project staff using the contact information provided later in this form. If you decide to withdraw from the project early, the Principal Investigator or project staff may ask you some questions about being in the project to gain feedback on ways in which biobank may be improved. The Principal Investigator or the sponsor can remove you from the project at any time, even if you want to stay in the project.

F. What kind of information could be found in this study and will I be able to see it?

QUORUM REVIEW
APPROVED
INSTITUTIONAL
REVIEW BOARD

This research is being done to add to our knowledge of Castleman Disease, and is not intended to provide information about your individual health or treatment. Investigators do not plan on returning the research test results to you, and the results will not be placed in your regular medical record. However, if a research test reveals something that could potentially affect your care (such as a possible misdiagnosis) they may contact your physician to have the tests confirmed in a clinical lab. Your physician, not the investigator, will then follow-up with you.

If you choose, the Castleman Disease Collaborative Network can also keep you up to date on the research outcomes of projects using samples or data from this biobank; however not all research projects will use samples from all the donors to the bank, so your own tissue may or may not have been part of a particular research project. You do not need to participate in this biobank to be kept up-to-date on the Castleman Disease Collaborative Network's research efforts; instead, you can sign up online for our email list here at cdcn.org. We regularly update the website.

If you choose, your samples and information may also be used for other central databanks. To allow research collaborations and sharing of data, the National Institutes of Health (NIH) and other research organizations have developed special data (information) repositories that analyze data and collect the results of certain types of genetic studies. These central banks will store your genetic information and provide them to qualified researchers to do more studies. This information will be available for any research question, such as research to understand what causes certain diseases (for example heart disease, cancer, or psychiatric disorders), development of new scientific methods, or the study of where different groups of people may have come from. We are also asking your permission to share your results with these special controlled-access banks. Controlled-access means that only researchers who apply for and get permission to use the information for a specific research project will be able to access the information. Your genomic data and health information will not be labeled with your name or other information that could be used to identify you. Researchers approved to access information in the database will agree not to attempt to identify you. Your information will be sent only with a code number attached. All directly identifiable information, such as your name, will not be shared with data banks or other investigators. Although there may be a small risk of loss of privacy when sharing this information with these banks, we have established procedures to encode your samples and information and protect your data. Although we will do everything we can to protect the privacy of your data, we cannot absolutely guarantee its privacy or predict how genetic information will be used in the future.

We will publish important discoveries found through these studies in the scientific literature so that the entire research community can work together to better understand Castleman Disease. Your individual data will not be published in a way in which you could be readily identified. Abstracts, which are plain language summaries of the published reports, will be available to you and the general public.

G. What are the risks or discomforts of the biobank project?

Your regular medical care will not change if you participate in this biobank project. The collection of tissue samples would only take place in the context of your routine care, and therefore does not add any physical risk to you that you would not already be undertaking. If you agree to give an additional blood sample, the medical staff will take your blood by sticking a needle in your arm or they may collect the

QUORUM REVIEW
APPROVED
INSTITUTIONAL
REVIEW BOARD

blood during surgery. The amount of blood that will be collected will not exceed 50 mL (about 3 tablespoons) over a 2-month period. Some problems you might have from this are:

- It may hurt.
- You may get a bruise.
- You may feel dizzy.
- You may get an infection.

There are some risks related to the use of your medical information and specimens. There could be an accidental release of your medical information to people who are not involved in your medical care. An accidental release of your genetic information could be used to identify you and your family members. We have tried to minimize this risk by carefully limiting access to the computers that would house your information and having other strict data security measures in place. There is still a risk someone could get access to the information we have stored about you, including information about a family member (for example, revealing that you or a blood relative carry a genetic disease). Because your DNA information is unique to you, there is a chance that someone could trace it back to you. The risk of this happening is small, but may be greater in the future.

Federal and State laws also provide some protections against discrimination based on genetic information. For example, the Genetic Information Nondiscrimination Act (GINA) makes it illegal for health insurance companies, group health plans, and most employers to discriminate against you based on your genetic information. However, it does not prevent companies that sell life insurance, disability insurance, or long-term care insurance from using genetic information as a reason to deny coverage or set premiums.

H. What are the benefits of the research study?

Research conducted with your information and specimens will not help you directly, but it may help people with Castleman in the future. Research performed with your information and specimens could result in the discovery and development of new drugs, tests or commercial products.

I. Can I stop being in the research study and what are my rights?

You can stop being in the research study at any time. You may choose to leave your samples and/or information in the biobank but request not to be re-contacted. Or, if you want to end all participation, the CDCN will destroy all of your sample identifiers and medical information in its possession; the CDCN will retain your original consent and the documentation of your withdrawal of consent. Any specimens or data that have already been distributed in a de-identified manner cannot be destroyed, and some research data may have already been published. If data has already been distributed to third parties, it cannot be retrieved (as it will have been de-identified). Any specimens or identifiable data you have given can be removed upon request, but any de-identified, already distributed information or specimens cannot be withdrawn.

QUORUM REVIEW
APPROVED
INSTITUTIONAL
REVIEW BOARD

To withdraw from research, you must notify, in writing, the Principal Investigator (Address: 1001 S. 18th St, A Philadelphia PA 19146). There are three ways you can choose to stop participating in the Biobank:

1. You may choose to leave your data and unused samples in the Biobank while requesting not to be re-contacted in the future.

2. You may request that your personally identifying information be destroyed, but allow some or all of your samples to continue being used anonymously for future research.

3. You can choose to end all participation, in which case all of the unused samples in the Biobank will be destroyed and your medical information will be deleted.

J. Will I be paid to take part in this research study?

You will not get payment for being in this biobank project. The sponsor does not plan to give you any money or payment resulting from any discoveries or products made from this research project.

K. What are the costs?

Participating in the biobank project should not cost you anything beyond your routine, scheduled medical costs. If you decide to donate saliva and blood samples, we recommend you get these drawn during your regularly scheduled clinical appointments. You and your insurance company will already be paying for the costs of the blood draws as a part of your standard medical care. That way there's no additional cost. If you don't have regularly scheduled follow-up but still want to participate, please visit an outpatient clinic to get your blood drawn. You will need to pay for the cost of the blood draw but the CDCN can reimburse you if you fill out a W2 form and provide a copy of the original receipt. If you have a future scheduled biopsy planned, you and your insurance company will already be paying for the costs of that procedure, so there will be no additional cost. Neither you nor your insurance company will be asked to pay for research tests.

L. What happens if I am injured or sick because I took part in this research study?

If injury (physical, psychological, social, financial, or otherwise) occurs as a result of participation in this project, there are no plans for providing compensation to you or your family.

Be aware that your health care payer/insurer might not cover the costs of study-related injuries.

You do not give up any of your legal rights by signing this form.

M. What about confidentiality?

Your individual data and health information (without your name, address, or other details that might identify you) will be put in a controlled-access database. This means that only researchers who apply for and get permission to use the information for a specific research project will be able to access the

QUORUM REVIEW
APPROVED
INSTITUTIONAL
REVIEW BOARD

information. Researchers approved to access information in the database will agree not to attempt to identify you. Access to information with your name, such as the consent form, will be restricted to healthcare staff, the Principal Investigator, designated CDCN staff, and approved CDCN contractors. Documents will be kept in a locked cabinet and information in computers will be protected by passwords. In order to protect your private information, only a unique code number, not your name or medical record number, will appear on your samples or on information sent to researchers outside the CDCN. Some people who work for the biobank will be able to find your name from the code so they can possibly have your sample(s) destroyed if you change your mind later. Except when required by law, your personally identifying information will not be disclosed outside of the CDCN.

N. Whom do I contact if I have questions about the research study?

If you have any questions about the study, please contact the research doctor or study staff listed below by email davidfajgenbaum@gmail.com or calling 215-573-8101:

- David Fajgenbaum, MD (Principal Investigator)

If you prefer to do so in writing, please send written correspondence to the address 1001 S. 18th St, A Philadelphia PA 19146.

You can learn more about the Castleman Disease Collaborative Network by visiting our website at www.cdcn.org.

Quorum Review Institutional Review Board (IRB) has reviewed this project. An IRB is a group of people who review research studies to protect the rights and welfare of research participants. If you have questions about your rights as a research participant, if you are not able to resolve your concerns with the Principal Investigator, if you have a complaint, or if you have general questions about what it means to be in a research project, you can call Quorum Review or visit the Quorum Review website at www.quorumreview.com.

Quorum Review is located in Seattle, Washington.

Office hours are 8:00 AM to 5:00 PM Pacific Time, Monday through Friday.

Ask to speak with a Research Participant Liaison at 888-776-9115 (toll free).

O. Conflict of Interest

Dr. David Fajgenbaum, the person responsible for the conduct of this research study also serves as the Executive Director of the CDCN. You should be aware that you are not under any obligation to participate in this research study.

P. Authorization to use your health information for research purposes

QUORUM REVIEW
APPROVED
INSTITUTIONAL
REVIEW BOARD

Because information about you and your health is personal and private, it generally cannot be used in this research study without your written authorization. Federal law called HIPAA requires that your health care providers and healthcare institutions (hospitals, clinics, doctor's offices) protect the privacy of information that identifies you and relates to your past, present, and future physical and mental health conditions.

If you agree to take part in this research, we will request that your health care providers and healthcare institutions disclose your protected health information to the Castleman Disease Collaborative Network for use in this research study. This may mean that you need to sign an additional form specifically authorizing release to the Network, but this will depend on your doctor's local practices, and we will work with you on this.

This section informs you about how your health information will be used or disclosed in the study. Your information will only be used in accordance with what is described here and as required or allowed by law. If you do not want to allow the uses described here, you cannot be in this biobank. You can still be in the biobank even if you do not give permission for the use and sharing of your information for the optional parts of the biobank.

1. What personal information about me will be used or shared with others during this research?

- Your medical records
- Your tissue samples relevant to this research study and related records
- New health information created from study-related tests and/or questionnaires

2. Why will protected information about me be used or shared with others?

The main reasons include the following:

- To conduct and oversee the research described earlier in this form;
- To ensure the research meets legal, institutional, and accreditation requirements;
- To conduct public health activities (including reporting of adverse events or situations where you or others may be at risk of harm)

3. Who will use or share protected health information about me?

With your consent, biobank staff will obtain information about you either directly from you or by having healthcare staff review your medical records. They may share your consent information in connection with the research study.

QUORUM REVIEW
APPROVED
INSTITUTIONAL
REVIEW BOARD

4. With whom outside of the Castleman Disease Collaborative Network may my personal health information be shared?

While all reasonable efforts will be made to protect the confidentiality of your protected health information, it may also be shared with the following entities:

- Federal and state agencies (for example, the Department of Health and Human Services, the Food and Drug Administration, the National Institutes of Health, and/or the Office for Human Research Protections), or other domestic or foreign government bodies if required by law and/or necessary for oversight purposes. A qualified representative of the FDA, the National Cancer Institute, or Quorum Review may review your medical records.

- Outside individuals or entities that have a need to access this information to perform functions relating to the conduct of this research such as data storage companies.

5. For how long will protected health information about me be used or shared with others?

There is no scheduled date at which your protected health information that is being used or shared for this research will be destroyed, because research is an ongoing process.

6. Statement of privacy rights:

- You have the right to withdraw your permission for the doctors and researchers to use or share your protected health information. We will not be able to withdraw all the information that already has been used or shared with others to carry out related activities such as oversight, or that is needed to ensure quality of the study. To withdraw your permission, you must do so in writing by contacting the researcher listed above in the section: "Whom do I contact if I have questions about the research study?" If you do so, you will no longer be a participant in this study. You can withdraw your permission for the optional parts of the biobank and still be in the biobank.

- After your information is shared with the Network, federal law will no longer require it be protected. The Network may also share your information with other people who the federal law does not apply too. The Network will nonetheless only use or disclose your information as described in this form and will have its own security measures in place to ensure your information and privacy remains protected.

Q. Participation Information

If you decide to sign this consent form, we will ask you for information about contacting your physicians and the hospitals that you were treated at for Castleman. We will not disclose details about the results of your participation in this study with any of the individuals that we contact, but rather ask them to provide us with your medical history and your tissue samples.

QUORUM REVIEW
APPROVED
INSTITUTIONAL
REVIEW BOARD

R. Consent

I have read this form, and I have had the opportunity ask questions about this study. I understand that my participation is voluntary, and if I decide not to participate it will have no impact on my medical care. If decide to participate now, I can decide to stop being in the study at any time. I voluntarily agree to take part in this research biobank. By signing this form, I do not give up any of my legal rights.

X Yes I Consent

REQUIRED ACTIVITIES:

I agree to the following activities as part of this biobank. (You must agree to all of the following to participate in the biobank. If you do not agree to all of these, you should not join the biobank.)

You can request my stored tissue samples (blood and/or tissue (lymph nodes, bone marrow)) from my doctors and hospitals. You can perform (or collaborate with others to perform) research on the samples, and store the samples until this research study is complete.

_ Yes I Agree

You can request my tissue samples (blood and/or tissue (lymph nodes, bone marrow)) from my currently scheduled medical procedures from my doctor and hospital. You can perform (or collaborate with others to perform) research on the samples, and store the samples until this research study is complete.

_ Yes I Agree

I am willing to provide leftovers from any future tissue samples that may be taken for my regular medical care (blood and/or tissue (lymph nodes, bone marrow)) to the biobank. I will allow research to be performed on the samples, and the samples to be stored until this research study is complete. (Even if you check this now, you can change your mind in the future.)

_ Yes I Agree

You can request my medical records from my doctors and the hospitals and other places where I received and/or continue to receive my treatment. You can link results of the research you perform on my samples with my medical information from my medical records.

_ Yes I Agree

I am willing to be re-contacted by study staff later to update my medical information. Even if I agree now by checking this box, I can decide not to provide this information when I am re-contacted.

QUORUM REVIEW
APPROVED
INSTITUTIONAL
REVIEW BOARD

You can use the results of the research on my samples and my medical information for future research **studies**, including studies that have not yet been designed, studies for diseases other than Castleman Disease, and/or studies that may be for commercial purposes.

_ Yes I Agree

You can share the results of the research on my samples and my medical information with central data banks (e.g., the NIH) and with other qualified researchers. It will be shared in a manner that does not include my name, social security number, or any other information that could be used to readily identify me. It can be used by other qualified researchers to perform future research studies, including studies that have not yet been designed, studies for diseases other than Castleman Disease, and studies that may be for commercial purposes.

_ Yes I Agree

OPTIONAL ACTIVITIES:

Please check below if you are willing to allow any of these optional activities as part of this research biobank. You can still be in the study even if you do not agree to any of these activities:

I am willing to have blood drawn for the biobank. You can perform (or collaborate with others to perform) research on the blood sample that I will send you and store the sample until this research study is complete.

_ Yes

I am willing to have my saliva collected for the biobank. You can perform (or collaborate with others to perform) research on the saliva sample that I will send you and store the sample until this research study is complete.

_ Yes

I am willing to be re-contacted by study staff later to provide new samples for research. Even if I agree now by checking this box, I can decide not to provide this information or additional samples when I am re-contacted.

_ Yes

You may re-contact me to notify me about clinical trials and other research opportunities.

_ Yes

QUORUM REVIEW
APPROVED
INSTITUTIONAL
REVIEW BOARD

I am willing to be re-contacted to obtain my consent in the event that return of my personal research results becomes possible.

_ Yes

You should print a copy of this form for your records.

Participant Signature

First

Last

Date of Participant Signature

MM/DD/YYYY

--NEXT PAGE--

If you selected “Parent of pediatric CD patient,” at the beginning of the survey please respond on behalf of the PATIENT

If you selected “Adult CD patient”/ “Related family member of CD patient”/ “Patient with related disease”/ “Healthy volunteer,” at the beginning of this survey, please enter YOUR information.

Please provide your current legal first name.

Please provide your current legal middle name. Please type in 0 if none.

Please provide your current legal last name.

Has your name changed since birth?

_ Yes

_ No

QUORUM REVIEW
APPROVED
INSTITUTIONAL
REVIEW BOARD

What is your date of birth?

MM/DD/YYYY

What was your physical sex at birth?

_ Male

_ Female

What city/municipality were you born in?

What country were you born in?

Please provide your current full mailing address below.

Street Address

Address Line 2

City

State / Province / Region

Postal / Zip Code

Country

_ Which of the following selections describes your ancestry? Please select all that apply.

_ American Indian or Alaska Native

_ Asian

_ Black or African American

QUORUM REVIEW
APPROVED
INSTITUTIONAL
REVIEW BOARD

_ Hispanic or Latino

_ Native Hawaiian or Other Pacific Islander

_ Caucasian, Non-Finnish Ancestry

_ Caucasian, Finnish Ancestry

_ I prefer not to say

_ Other (please specify)

Please enter your primary phone number where we may contact you if needed.

Please enter your email address.

--NEXT PAGE—

If you selected "Parent of pediatric CD patient," at the beginning of this survey please respond on behalf of the PATIENT.

If you selected "Adult CD patient"/ "Related family member of CD patient"/ "Patient with related disease"/ "Healthy volunteer," at the beginning of this survey, please enter YOUR information.

Are you interested in donating a blood sample?

_ Yes

_ No

Are you interested in having your existing tissue block (lymph node and/or bone marrow) transferred?

_ Yes

_ No

If you answered "yes" to either of the previous questions, could you please provide the name and city/state of all of the hospitals where you may have samples stored (Please list all hospitals even if you are unsure if samples remain)?

QUORUM REVIEW
APPROVED
INSTITUTIONAL
REVIEW BOARD

--NEXT PAGE—

This is the next section of the CDCN Survey.

Please select "Next Page" to continue with the survey. Depending on your prior response, certain pages will not apply to you and you will automatically bypass them.

--NEXT PAGE—

If you selected “Parent of pediatric CD patient,” at the beginning of this survey please respond on behalf of the PATIENT.

If you selected “Adult CD patient”/ “Related family member of CD patient”/ “Patient with related disease”/ “Healthy volunteer,” at the beginning of this survey, please enter YOUR information.

Please list full name of all physicians and institutions.

What is the name of your current Castleman disease treating physician(s) and their corresponding institution(s)?

Please list any other treating physician(s) and their corresponding institution(s) that you have seen since you Castleman disease symptoms started?

Have you had a lymph node biopsy pathology report state "consistent with", "suggestive of", "compatible with", or "diagnostic of" Castleman disease?

_ Yes

QUORUM REVIEW
APPROVED
INSTITUTIONAL
REVIEW BOARD

_ No

Has your clinician diagnosed you with unicentric (enlargement of a single region of lymph nodes) or multicentric (enlargement of more than one region of lymph nodes) Castleman disease?

_ Unicentric

_ Multicentric

_ Unsure

What date were you diagnosed?

MM/DD/YYYY

Have you been told that you have or may have TAFRO syndrome?

_ Yes

_ No

_ Unsure

Have you ever been diagnosed with HIV?

_ Yes

_ No

_ I do not feel comfortable answering this question (prefer not to answer)

Has your clinician told you if your disease is HHV-8 positive, HHV-8-negative, or do you not know?

_ HHV-8-positive

_ HHV-8-negative

_ Do Not Know

If you had a HHV-8 test done, which test was performed to determine your HHV-8 status?

_ LANA 1

_ PCR of blood

QUORUM REVIEW
APPROVED
INSTITUTIONAL
REVIEW BOARD

_ PCR of Lymph node

_ Unsure

Which of the following clinical features have you experienced during active disease/flare? Please select "None" if you did not experience any.

_ None

Clinical features experienced during active disease/flare:

_ Flu-like symptoms

_ Night sweats

_ Fevers (>100.5F)

_ Weight loss

_ Fatigue

_ Large spleen and/or liver

_ Edema/anasarca, effusions, ascites, or other form of fluid overload

_ Eruption of cherry hemangiomas on the skin or violaceous papules

_ Lymphocytic Interstitial pneumonitis

Do you have any other symptoms believed to be related to Castleman disease? If so, please list these symptoms. Also, please comment on the severity of these symptoms and/or the above symptoms. Please type in NA if this does not apply to you.

Which of the following laboratory abnormalities have you experienced during active disease/flare? Please select "None" if you did not experience any.

_ None

Laboratory abnormalities experienced during active disease/flare: *

_ Elevated C-Reactive Protein (CRP) (>10mg/dL)

_ Elevated Erythrocyte Sedimentation Rate (ESR) (>15mm/hr)

QUORUM REVIEW
APPROVED
INSTITUTIONAL
REVIEW BOARD

_ Low Hemoglobin/Anemia (Hgc<12.5 for males, <11.5 for females)

_ Elevated Platelets/Thrombocytosis (>400k/uL)

_ Low Platelets/Thrombocytopenia (<150K/uL)

_ Low Albumin/Hypoalbuminemia (<3.5 gm/dL)

_ Kidney dysfunction (elevated Creatinine or BUN) or proteinuria (protein in urine)

_ High Gamma Globin

_ Levels/Hypergammaglobulinemia (>1700mg/dL)

Do you have any other laboratory abnormalities believed to be related to Castleman disease? If so, please list these symptoms. Also, please comment on the severity of these laboratory abnormalities and/or the above laboratory abnormalities. Please type in NA if this does not apply to you.

Did you have a bone marrow biopsy during active disease/flare? Please specify if the pathology report stated fibrosis, myelofibrosis, or reticulin fibrosis.

_ Yes, stated fibrosis/myelofibrosis/reticulin fibrosis

_ Yes, did not specify

_ No, did not have bone marrow biopsy done

_ Unsure

Have you ever been hospitalized for Castleman disease or associated complications? If so, for how long?

_ Never been hospitalized

_ Less than 3 days

_ 3 – 6 days

_ 1 – 4 weeks

_ More than 4 weeks

Please select any disorders listed below that you have been diagnosed with or select "Other" if you have been diagnosed with a disease not on this list.

_ Clinical Epstein Barr Virus (EBV)

QUORUM REVIEW
APPROVED
INSTITUTIONAL
REVIEW BOARD

_ lymphoproliferative disorders

_ Cytomegalovirus (CMV)

_ Toxoplasmosis

_ Active Tuberculosis

_ Systemic lupus erythematosus

_ Rheumatoid arthritis

_ Adult-onset Still's disease

_ Juvenile Idiopathic Arthritis

_ IgG4-related disease

_ Hodgkin's lymphoma

_ Non-Hodgkin's lymphoma

_ Multiple myeloma

_ Primary lymph node plasmacytoma

_ Follicular dendritic cell sarcoma

_ POEMS syndrome

_ Other (Please Specify)

If you selected "Other", please specify the disease(s).

Please supply any reports or medical records associated with those diagnoses if you have any.

[FILE UPLOAD BUTTON]

--NEXT PAGE—

If you selected “Parent of pediatric CD patient,” at the beginning of this survey please respond on behalf of the PATIENT.

If you selected “Adult CD patient”/ “Related family member of CD patient”/ “Patient with related disease”/ “Healthy volunteer,” at the beginning of this survey, please enter YOUR information.

QUORUM REVIEW
APPROVED
INSTITUTIONAL
REVIEW BOARD

What was the first treatment regimen you received for your Castleman disease (CD)? (Examples of treatment regimen include Prednisone, Siltuximab, Siltuximab+ Prednisone, CHOP, Tocilizumab, Tocilizumab+Prednisone, Surgery, Radiation, Stem Cell Transplant, etc.)

What was the date you began your first treatment regimen?

MM/DD/YYYY

Is this first treatment regimen still ongoing?

_ Yes

X No

What is the date you discontinued this treatment regimen?

MM/DD/YYYY

Do you believe that your symptoms/laboratory values improved on treatment completely, partially, or not at all as a result of your first treatment regimen?

_ Complete improvement in Castleman disease symptoms

X Partial improvement in Castleman disease symptoms

_ No improvement in Castleman disease symptoms

If you improved on your first treatment regimen, did you ever experience progression of the disease at a later time?

_ Yes

_ No

What was the date that your disease progressed?

MM/DD/YYYY

Have you received a second treatment regimen?

QUORUM REVIEW
APPROVED
INSTITUTIONAL
REVIEW BOARD

X Yes

_ No

What was the second treatment regimen you received for your Castleman disease (CD)? (Examples of treatment regimen include Prednisone, Siltuximab, Siltuximab+ Prednisone, CHOP, Tocilizumab, Tocilizumab+Prednisone, Surgery, Radiation, Stem Cell Transplant, etc.)

What was the date you began your second treatment regimen?

MM/DD/YYYY

Is this second treatment regimen still ongoing?

X Yes

_ No

What is the date you discontinued this treatment regimen?

MM/DD/YYYY

Do you believe that your symptoms/laboratory values improved on treatment completely, partially, or not at all as a result of your first treatment regimen?

X Complete improvement in Castleman disease symptoms

_ Partial improvement in Castleman disease symptoms

_ No improvement in Castleman disease symptoms

If you improved on your second treatment regimen, did you ever experience progression of the disease at a later time?

X Yes

_ No

What was the date that your disease progressed?

QUORUM REVIEW
APPROVED
INSTITUTIONAL
REVIEW BOARD

MM/DD/YYYY

Have you received a third treatment regimen?

_ Yes

X No

--NEXT PAGE—

If you selected "Parent of pediatric CD patient," at the beginning of this survey please respond on behalf of the PATIENT.

If you selected "Adult CD patient"/ "Related family member of CD patient"/ "Patient with related disease"/ "Healthy volunteer," at the beginning of this survey, please enter YOUR information.

When was the last time that you had blood work done?

MM/DD/YYYY

When was the last time that you visited your physician?

MM/DD/YYYY

Please upload a copy of the results of your most recent blood work or clinical note here if you have a copy

[FILE UPLOAD BUTTON]

At time of this blood draw, do you have active disease/flare (e.g. presence of symptoms related to your Castleman disease)?

X Yes

_ No

At what stage of the disease do you feel you are in currently?

QUORUM REVIEW
APPROVED
INSTITUTIONAL
REVIEW BOARD

_ Early stages of disease activity

_ At the peak of disease activity

_ Improving since worst disease activity

When was the last time you experienced active disease/flare (presence of symptoms related to your Castleman disease)?

MM/DD/YYYY

Which of the following clinical features are you currently experiencing? Please select "None" if you have not experienced any or select the items from the corresponding list.

_ None

Clinical features "Currently" experienced:

_ Flu-like symptoms

_ Night sweats

_ Fevers (>100.5F)

_ Weight loss

_ Fatigue

_ Large spleen and/or liver

_ Edema/anasarca, effusions, ascites, or other form of fluid overload

_ Eruption of cherry hemangiomas on the skin or violaceous papules

_ Lymphocytic Interstitial pneumonitis

Which of the following laboratory abnormalities did you experience when your blood was last drawn? Please select "None" if you have not experienced any or select the items from the corresponding list.

_ None

Laboratory abnormalities experience when your blood was last drawn:

_ Elevated C-Reactive Protein (CRP) (>10mg/dL)

_ Elevated Erythrocyte Sedimentation Rate (ESR) (>15mm/hr)

QUORUM REVIEW
APPROVED
INSTITUTIONAL
REVIEW BOARD

_ Low Hemoglobin/Anemia (Hgc<12.5 for males, <11.5 for females)

_ Elevated Platelets/Thrombocytosis (>400k/uL)

_ Low Platelets/Thrombocytopenia (<150K/uL)

_ Low Albumin/Hypoalbuminemia (<3.5 gm/dL)

_ Kidney dysfunction (elevated Creatinine or BUN) or proteinuria (protein in urine)

_ High Gamma Globin

_ Levels/Hypergammaglobulinemia (>1700mg/dL)

Are you currently registered in the ACCELERATE registry? (Please visit http://www.cdcn.org/accelerate to get more details if you are interested in joining.)

_ Yes

_ No

Are you currently on any medications (for Castleman disease or other conditions)?

X Yes

_ No

Please list any medications that you are currently taking.

--NEXT PAGE—

Thank you for completing our survey! We really appreciate your support in fighting back against Castleman disease.

The CDCN will review the information you have provided and determine how you can be involved in providing samples for research. We will be in touch once we have gathered enough information to be able to send you a kit in the mail with empty tubes or if we have any questions for you.

QUORUM REVIEW
APPROVED
INSTITUTIONAL
REVIEW BOARD

Related family member of CD patient (without CD and > 18 and NOT parent of pediatric CD patient)

Thank you for filling out this Survey, so that we will be able to include your samples in the BioBank.

If you are presented with a Survey Page that does not contain any questions, please select "Next Page" to continue through the Survey. That particular Survey Page does not contain any questions that pertain to you based on your prior responses.

If you have any questions, please contact Dustin Shilling at 1-215-614-0689 or dustin@castlemannetwork.org

The following form will enable you to participate in the CDCN Biobank (Castlebank). Tissue samples such as blood or excess biopsy samples from patients will enable scientists to better characterize and understand Castleman disease and develop better treatments. Any blood or tissue donation will be used for high-impact research to battle this disease!

Is this your first time filling out this Survey? (If you do not remember if you have participated in the past, please call 1-215-614-0689)

X First Time Participant

_ Repeat Participant

--NEXT PAGE--

Please select the appropriate category that describes the PERSON completing this survey. (This question refers to the person completing the survey, NOT necessarily the patient)

_ Adult CD patient (> 18 years old)

_ Parent of pediatric CD patient

X Related family member of CD patient (without CD and > 18 and NOT parent of pediatric CD patient)

_ Patient with related disease (e.g. Lupus, Rheumatoid Arthritis and > 18 years old)

_ Healthy volunteer (> 18 years old)

--NEXT PAGE--

QUORUM REVIEW
APPROVED
INSTITUTIONAL
REVIEW BOARD

RESEARCH CONSENT FORM – KEY POINTS

For Family Members, Healthy Controls, or Similar Disease Controls

Study Title: The Castleman Disease Collaborative Network Biobank: A Collection of Biospecimens and Clinical Data to Facilitate Research
Study #: 001
Sponsor: Castleman Disease Collaborative Network
Study Investigator: David Fajgenbaum
Castleman Disease Collaborative Network
1001 S. 18th St., Unit A., Philadelphia, PA 19146
Telephone Number: (610) 304-0696

The goal of the "Castleman Disease Collaborative Network Biobank" project is to be a patient-driven movement that empowers people with Castleman Disease to directly transform research and treatment of disease by providing blood, sharing part of their stored tissue, and copies of their medical records for use in a variety of research projects. Because we are enrolling people with Castleman across the country regardless of where they are being treated, this study will allow many more people to contribute to research than has previously been possible. We are also inviting family members of people with Castleman Disease, healthy individuals, as well as individuals who have diseases that share similar characteristics to Castleman Disease to use as comparators to patients with Castleman Disease.

1. What is the purpose of this study?

We want to understand Castleman Disease better so that we can develop more effective therapies. The Castleman Disease Collaborative Network would like to partner directly with people with Castleman Disease, so we can study many more aspects of Castleman Disease than would otherwise be possible.

2. What will I have to do if I agree to participate in this study?

You will answer some questions about your medical history and send a blood and/or saliva sample to us in a pre-stamped package that we will provide.

3. Do I have to participate in this study?

No. Taking part in this study is voluntary. Even if you decide to participate, you can always change your mind and leave the study.

QUORUM REVIEW
APPROVED
INSTITUTIONAL
REVIEW BOARD

4. Will I benefit from participating?

While taking part in this study will not improve your own health, the information we collect will aid in research efforts to provide better treatment and prevention options to people with Castleman in the future. We will provide updates about key research discoveries made possible by this biobank on our website, and directly to you via email if you so choose.

5. What are the risks of taking part in this research?

There may be a risk that your information (which includes your genetic information and medical history information) could be seen by unauthorized individuals. However, we have procedures and security measures in place designed to minimize this risk and protect the confidentiality of your information.

If you decide to give blood for this biobank, you may experience pain, bruising, dizziness, or an infection.

6. Will it cost me anything to participate in this study?

No.

7. Who will use my samples and see my information?

Your samples and health information will be available to researchers at the Castleman Disease Collaborative Network (CDCN), a not-for-profit global initiative dedicated to accelerating research and treatment for Castleman disease. Your samples may also be used by outside researchers, both in the U.S. and internationally. After removing your name and other readily identifiable information, we will share tissues with qualified researchers, who have undergone review and approval by a CDCN Advisory Board. Results from the research studies may be shared with the greater research community via publications, as well as central data banks at the National Institutes of Health.

8. Can I stop taking part in this research study?

Yes, you can withdraw from this research study at any time. CDCN will destroy all your samples in its possession; if samples and information have already been sent to researchers, the CDCN will notify the researcher of the participant's wishes but the CDCN cannot guarantee these samples will be destroyed. In addition, research data may have already been published before you withdrew and that cannot be retracted. Your information would be removed from future studies.

9. What if I have questions?

QUORUM REVIEW
APPROVED
INSTITUTIONAL
REVIEW BOARD

If you have any questions, please contact the Principal Investigator, Dr. David Fajgenbaum at (215) 614-0936. In case you want to inquire via writing, their address is 1001 S. 18th St, A Philadelphia PA 19146.

FULL RESEARCH CONSENT FORM

For Family Members and Healthy Controls

The Castleman Disease Collaborative Network Biobank: A Collection of Biospecimens and Clinical Data to Facilitate Research

A. Introduction

You are being invited to participate in a research study that will establish a Castleman Disease biobank. A biobank, sometimes called a biorepository, collects, stores, analyzes and distributes samples of human tissues, blood, and related health information to qualified scientists. Creation of a biobank for this disease may help doctors and researchers better understand why Castleman Disease occurs and develop ways to better treat and prevent it. Because Castleman Disease is so rare, it has not been well studied, due in part to a lack of available biospecimens and data for research use.

You are being asked to participate in the study because you are either a family member of someone with Castleman Disease, or are a healthy individual. You are being asked to provide blood or saliva samples. In order to execute meaningful research, studies require a comparator in order to determine what makes people with the disease different. Family members and healthy individuals who serve as these comparators or “controls” provide information that can be used to compare to people with Castleman, to better understand the biology of Castleman Disease.

The biobank is being established by the Castleman Disease Collaborative Network (CDCN), a global initiative dedicated to accelerating research and treatment for Castleman Disease. We work to achieve this by facilitating collaboration among the global research community, mobilizing resources, strategically investing in high-impact research, and supporting people with Castleman Disease and their family members. This biobank is paid for by the CDCN.

This form describes the project in order to help you decide if you want to participate. This form will tell you what you will have to do and the risks and benefits of participating.

If you have any questions about or do not understand something in this form, you should ask the research staff. You should also discuss your participation with anyone you choose in order to better understand this project and your options. Do not sign this form unless the staff has answered your questions and you decide that you want to be part of this project.

When reading this form, please note that the words “you” and “your” refer to the person in the project.

B. What is the purpose of this biobank?

QUORUM REVIEW
APPROVED
INSTITUTIONAL
REVIEW BOARD

The Castleman Disease Collaborative Network is creating this biobank to support research. We want scientists to better understand Castleman Disease and work toward developing more effective therapies. We will be able to study many more aspects of Castleman than has previously been possible.

By participating in this biobank project, you will be asked to provide blood and/or saliva now or in the future as well as medical information to be used for research. We would also like your permission to access and share (after removing your name and other personal identifiers) your medical information along with your specimens.

Some of the research might involve studying DNA contained in your samples. DNA is the material that makes up genes. Genes determine things like hair color and eye color, and may play a role in Castleman Disease. Using DNA from your blood or tissue sample, researchers will study your entire genetic sequence, known as your genome, and store this information. Your genomic data and health information will be studied along with information from other participants in this study, and it will be stored for future studies.

Samples may be studied immediately or may be stored for future use by researchers from universities, the government, health-related companies, and other research institutes. Some researchers will be from the United States and some may be from other countries around the world. A Biobank Advisory Board (BAB) composed of physicians and scientists will review research requests to help ensure that tissues are being utilized in the most impactful manner. The BAB will not to attempt to re-identify you. Future researchers must also sign an agreement indicating they will not attempt to re-identify you. Because technology is changing all the time it is not possible to know all of the techniques that researchers may use now or in the future.

We are asking many people to participate in the biobank, both people with Castleman, their family members, and healthy individuals (as a comparison group). We don't know how many people will participate.

C. What other options are there?

Your participation in the biobank project is voluntary. Your alternative is not to participate. The decision not to participate or to stop participating at any time will not involve any penalty to you and will not involve any loss of benefits to which you might otherwise be entitled.

D. What is involved in the biobank project?

With your consent, project staff will collect medical information through a secure questionnaire regarding your health, medical, and family history.

The types of samples the Castleman Disease Collaborative Network wants to collect from family members and health controls include:

- blood samples already stored, scheduled to be taken, or that may be taken in the future
- saliva

QUORUM REVIEW
APPROVED
INSTITUTIONAL
REVIEW BOARD

Each type of sample collection is optional. You can decide to participate in each type of sample collection separately, which means you can agree to some, all, or none of the different types of samples. You can indicate your choice at the end of this form.

If you consent to have blood drawn for the biobank, it may be collected at the same time as routine medical visits, if possible. The biobank will send you blood collection kits with tubes and instructions that you can take to your doctor or local laboratory so they can obtain one or more blood samples. The amount of blood collected will be no greater than 50 mL (about 3 tablespoons) over a 2-month period.

If you give your consent to be re-contacted, biobank staff may contact you periodically to update your medical information or ask you to provide new samples for research. You may also choose if you would like the Castleman Disease Collaborative Network to notify you about other research studies in the future.

People who work with the sponsor (CDCN) will store your samples in a secure laboratory. Your samples will be stored as long as the sponsor decides to keep them.

E. How long will I be in this research study?

Your direct involvement will be limited initially to the time it takes to obtain your consent and to collect samples and medical information. Because this project involves a biobank, your samples, data, and health information may be used immediately or may be stored indefinitely for future use. If you agree, we may also re-contact you to get updates about your health and medical care, to ask whether you will give additional samples, or to tell you about other studies.

If you want to stop being in the biobank project, contact the Principal Investigator or project staff using the contact information provided later in this form. If you decide to withdraw from the project early, the Principal Investigator or project staff may ask you some questions about being in the project to gain feedback on ways in which biobank may be improved. The Principal Investigator or the sponsor can remove you from the project at any time, even if you want to stay in the project.

F. What kind of information could be found in this study and will I be able to see it?

This research is being done to add to our knowledge of Castleman Disease, and is not intended to provide information about your individual health. Investigators do not plan on returning the research test results to you, and the results will not be placed in your regular medical record. However, if a research test reveals something that could potentially affect your medical care, they may contact your physician to have the tests confirmed in a clinical lab. Your physician, not the investigator, will then follow-up with you. We will obtain consent from you to provide your physician your research results and ask you for updated physician contact information at that time.

If you choose, the Castleman Disease Collaborative Network can also keep you up to date on the research outcomes of projects using samples or data from this biobank; however not all research projects will use samples from all the donors to the bank, so your own tissue may or may not have been part of a particular research project. You do not need to participate in this biobank to be kept up-to-date

QUORUM REVIEW
APPROVED
INSTITUTIONAL
REVIEW BOARD

on the Castleman Disease Collaborative Network's research efforts; instead, you can sign up online for our email list here at cdcn.org. We regularly update the website.

If you choose, your samples and information may also be used for other central databanks. To allow research collaborations and sharing of data, the National Institutes of Health (NIH) and other research organizations have developed special data (information) repositories that analyze data and collect the results of certain types of genetic studies. These central banks will store your genetic information and provide them to qualified researchers to do more studies. This information will be available for any research question, such as research to understand what causes certain diseases (for example heart disease, cancer, or psychiatric disorders), development of new scientific methods, or the study of where different groups of people may have come from. We are also asking your permission to share your results with these special controlled-access banks. Controlled-access means that only researchers who apply for and get permission to use the information for a specific research project will be able to access the information. Your genomic data and health information will not be labeled with your name or other information that could be used to identify you. Researchers approved to access information in the database will agree not to attempt to identify you. Your information will be sent only with a code number attached. All directly identifiable information, such as your name, will not be shared with data banks or other investigators. Although there may be a small risk of loss of privacy when sharing this information with these banks, we have established procedures to encode your samples and information and protect your data. Although we will do everything we can to protect the privacy of your data, we cannot absolutely guarantee its privacy or predict how genetic information will be used in the future.

Furthermore, we will publish important discoveries found through these studies in the scientific literature so that the entire research community can work together to better understand Castleman Disease. Your individual data will not be published in a way in which you could be readily identified. Abstracts, which are plain language summaries of the published reports, will be available to you and the general public.

G. What are the risks or discomforts of the biobank project?

Your regular medical care will not change if you participate in this biobank project. If you agree to give a blood sample, the medical staff will take your blood by sticking a needle in your arm. The amount of blood that will be collected will not exceed 50 mL (about 3 tablespoons) over a 2-month period. Some problems you might have from this are:

- It may hurt.
- You may get a bruise.
- You may feel dizzy.
- You may get an infection.

There are some risks related to the use of your medical information and specimens. There could be an accidental release of your medical information to people who are not involved in your medical care. An accidental release of your genetic information could be used to identify you and your family members. We have tried to minimize this risk by carefully limiting access to the computers that would house your

QUORUM REVIEW
APPROVED
INSTITUTIONAL
REVIEW BOARD

information and having other strict data security measures in place. There is still a risk someone could get access to the information we have stored about you, including information about a family member (for example, revealing that you or a blood relative carry a genetic disease). Because your DNA information is unique to you, there is a chance that someone could trace it back to you. The risk of this happening is small, but may be greater in the future.

Federal and State laws also provide some protections against discrimination based on genetic information. For example, the Genetic Information Nondiscrimination Act (GINA) makes it illegal for health insurance companies, group health plans, and most employers to discriminate against you based on your genetic information. However, it does not prevent companies that sell life insurance, disability insurance, or long-term care insurance from using genetic information as a reason to deny coverage or set premiums.

H. What are the benefits of the research study?

Research conducted with your information and specimens will not help you directly, but it may help people with Castleman in the future. Research performed with your information and specimens could result in the discovery and development of new drugs, tests or commercial products.

I. Can I stop being in the research study and what are my rights?

You can stop being in the research study at any time. You may choose to leave your samples and/or information in the biobank but request not to be re-contacted. Or, if you want to end all participation, the CDCN will destroy all of your sample identifiers and medical information in its possession; the CDCN will retain your original consent and the documentation of your withdrawal of consent. Any specimens or data that have already been distributed in a de-identified manner cannot be destroyed, and some research data may have already been published. If data has already been distributed to third parties, it cannot be retrieved (as it will have been de-identified). Any specimens or identifiable data you have given can be removed upon request, but any de-identified, already distributed information or specimens cannot be withdrawn.

To withdraw from research, you must notify, in writing, the Principal Investigator (Address: 1001 S. 18th St, A Philadelphia PA 19146). There are three ways you can choose to stop participating in the Biobank:

1. You may choose to leave your data and unused samples in the Biobank while requesting not to be re-contacted in the future.

2. You may request that your personally identifying information be destroyed, but allow some or all of your samples to continue being used anonymously for future research.

3. You can choose to end all participation, in which case all of the unused samples in the Biobank will be destroyed and your medical information will be deleted.

J. Will I be paid to take part in this research study?

QUORUM REVIEW
APPROVED
INSTITUTIONAL
REVIEW BOARD

You will not get payment for being in this biobank project. The sponsor does not plan to give you any money or payment resulting from any discoveries or products made from this research project.

K. What are the costs?

Participating in the biobank project should not cost you anything beyond your routine, scheduled medical costs. If you decide to donate saliva and blood samples, we recommend you get these drawn during your regularly scheduled clinical appointments. You and your insurance company will already be paying for the costs of the blood draws as a part of your standard medical care. That way there's no additional cost. If you don't have regularly scheduled follow-up but still want to participate, please visit an outpatient clinic to get your blood drawn. You will need to pay for the cost of the blood draw but the CDCN can reimburse you if you fill out a W2 form and provide a copy of the original receipt. Neither you nor your insurance company will be asked to pay for research tests.

L. What happens if I am injured or sick because I took part in this research study?

If injury (physical, psychological, social, financial, or otherwise) occurs as a result of participation in this project, there are no plans for providing compensation to you or your family.

Be aware that your health care payer/insurer might not cover the costs of study-related injuries.

You do not give up any of your legal rights by signing this form.

M. What about confidentiality?

Your individual data and health information (without your name, address, or other details that might identify you) will be put in a controlled-access database. This means that only researchers who apply for and get permission to use the information for a specific research project will be able to access the information. Researchers approved to access information in the database will agree not to attempt to identify you. Access to information with your name, such as the consent form, will be restricted to healthcare staff, the Principal Investigator, designated CDCN staff, and approved CDCN contractors. Documents will be kept in a locked cabinet and information in computers will be protected by passwords. In order to protect your private information, only a unique code number, not your name or medical record number, will appear on your samples or on information sent to researchers outside the CDCN. Some people who work for the biobank will be able to find your name from the code so they can possibly have your sample(s) destroyed if you change your mind later. Except when required by law, your personally identifying information will not be disclosed outside of the Castleman Disease Collaborative Network.

N. Whom do I contact if I have questions about the research study?

If you have any questions about the study, please contact the research doctor or study staff listed below by email davidfajgenbaum@gmail.com or calling 215-614-0936:

QUORUM REVIEW
APPROVED
INSTITUTIONAL
REVIEW BOARD

- David Fajgenbaum, MD (Principal Investigator)

If you prefer to do so in writing, please send written correspondence to the address 1001 S. 18th St, A Philadelphia PA 19146.

You can learn more about the Castleman Disease Collaborative Network by visiting our website at www.cdcn.org.

Quorum Review Institutional Review Board (IRB) has reviewed this project. An IRB is a group of people who review research studies to protect the rights and welfare of research participants. If you have questions about your rights as a research participant, if you are not able to resolve your concerns with the Principal Investigator, if you have a complaint, or if you have general questions about what it means to be in a research project, you can call Quorum Review or visit the Quorum Review website at www.quorumreview.com.

Quorum Review is located in Seattle, Washington.

Office hours are 8:00 AM to 5:00 PM Pacific Time, Monday through Friday.

Ask to speak with a Research Participant Liaison at 888-776-9115 (toll free).

O. Conflict of Interest

Dr. David Fajgenbaum, the person responsible for the conduct of this research study also serves as the Executive Director of the CDCN. You should be aware that you are not under any obligation to participate in this research study.

P. Authorization to use your health information for research purposes

Because information about you and your health is personal and private, it generally cannot be used in this research study without your written authorization. This section is intended to inform you about how your health information will be used or disclosed in the study. Your information will only be used as described here and as required or allowed by law.

If you do not want to allow the uses described here, you cannot be in this biobank. You can still be in the biobank even if you do not give permission for the use and sharing of your information for the optional parts of the biobank.

1. What personal information about me will be used or shared with others during this research?

- Your tissue samples relevant to this research study and related records
- New health information created from study-related tests and/or questionnaires

2. Why will protected information about me be used or shared with others?

QUORUM REVIEW
APPROVED
INSTITUTIONAL
REVIEW BOARD

The main reasons include the following:

- To conduct and oversee the research described earlier in this form;
- To ensure the research meets legal, institutional, and accreditation requirements;
- To conduct public health activities (including reporting of adverse events or situations where you or others may be at risk of harm)

3. Who will use or share protected health information about me?

With your consent, biobank staff will obtain information about your health from you, and they may share your consent information in connection with the research study.

4. With whom outside of the Castleman Disease Collaborative Network may my personal health information be shared?

While all reasonable efforts will be made to protect the confidentiality of your protected health information, it may also be shared with the following entities:

- Federal and state agencies (for example, the Department of Health and Human Services, the Food and Drug Administration, the National Institutes of Health, and/or the Office for Human Research Protections), or other domestic or foreign government bodies if required by law and/or necessary for oversight purposes. A qualified representative of the FDA, the National Cancer Institute, and Quorum Review may review your medical records.
- Outside individuals or entities that have a need to access this information to perform functions relating to the conduct of this research such as data storage companies.

5. For how long will protected health information about me be used or shared with others?

There is no scheduled date at which your protected health information that is being used or shared for this research will be destroyed, because research is an ongoing process.

6. Statement of privacy rights:

- You have the right to withdraw your permission for the doctors and researchers to use or share your protected health information. We will not be able to withdraw all the information that already has been used or shared with others to carry out related activities such as oversight, or that is needed to ensure quality of the study. To withdraw your permission, you must do so in writing by contacting the researcher listed above in the section: “Whom do I contact if I have questions about the research study?” If you do so, you will no longer be a participant in this study. You can withdraw your permission for the optional parts of the biobank and still be in the biobank.

QUORUM REVIEW
APPROVED
INSTITUTIONAL
REVIEW BOARD

•	After your information is shared with the Network, federal law will no longer require it be protected. The Network may also share your information with other people who the federal law does not apply too. The Network will nonetheless only use or disclose your information as described in this form and will have its own security measures in place to ensure your information and privacy remains protected.

Q. Consent

I have read this form, and I have been able to ask questions about this study. I understand that my participation is voluntary, and if I decide not to participate it will have no impact on my medical care. If decide to participate now, I can decide to stop being in the study at any time. I voluntarily agree to take part in this research biobank. By signing this form, I do not give up any of my legal rights.

I Consent

_ Yes I Consent

REQUIRED ACTIVITIES:

I agree to the following activities as part of this biobank. (You must agree to all of the following to participate in the biobank. If you do not agree to all of these, you should not join the biobank.)

I am willing to provide any future specimens to the biobank. (Even if you check this now, you can change your mind in the future.)

_ Yes I Agree

You can use the results of the research on my samples and my medical information for future research studies, including studies that have not yet been designed, studies for diseases other than Castleman disease, and/or studies that may be for commercial purposes.

_ Yes I Agree

You can share the results of the research on my samples and my medical information with central data banks (e.g., the NIH) and with other qualified researchers. It will be shared in a manner that does not include my name, social security number, or any other information that could be used to readily identify me, to be used by other qualified researchers to perform future research studies, including studies that have not yet been designed, studies for diseases other than Castleman disease, and studies that may be for commercial purposes.

QUORUM REVIEW
APPROVED
INSTITUTIONAL
REVIEW BOARD

_ Yes I Agree

OPTIONAL ACTIVITIES:

Please check below if you are willing to allow any of these optional activities as part of this research biobank. You can still be in the study even if you do not agree to any of these activities:

I am willing to have blood drawn for the biobank. You can perform (or collaborate with others to perform) research on the blood sample that I will send you and store the sample until this research study is complete.

_ Yes I Agree

I am willing to have my saliva collected for the biobank. You can perform (or collaborate with others to perform) research on the saliva sample that I will send you and store the sample until this research study is complete.

_ Yes I Agree

I am willing to be re-contacted by study staff later, to update my medical information or provide new samples for research. Even if I agree now by checking this box, I can decide not to provide this information or additional samples when I am re-contacted.

_ Yes I Agree

I am willing to be re-contacted to obtain my consent in the event that return of my personal research results becomes possible.

_ Yes I Agree

You can request my stored tissue samples (blood and/or tissue (lymph nodes, bone marrow)) from my physicians and the hospitals and other places where I received my care, perform (or collaborate with others to perform) research on the samples, and store the samples until this research study is complete.

_ Yes I Agree

You should print a copy of this form for your records.

QUORUM REVIEW
APPROVED
INSTITUTIONAL
REVIEW BOARD

Signature of Participant

First

Last

Date of Participant Signature

MM/DD/YYYY

--NEXT PAGE—

If you selected “Parent of pediatric CD patient,” at the beginning of this survey please respond on behalf of the PATIENT.

If you selected “Adult CD patient”/ “Related family member of CD patient”/ “Patient with related disease”/ “Healthy volunteer,” at the beginning of this survey, please enter YOUR information.

Please provide your current legal first name.

Please provide your current legal middle name. Please type in 0 if none.

Please provide your current legal last name.

Has your name changed since birth?

_ Yes

_ No

What is your date of birth?

MM/DD/YYYY

QUORUM REVIEW
APPROVED
INSTITUTIONAL
REVIEW BOARD

What was your physical sex at birth?

_Male

_Female

What city/municipality were you born in?

What country were you born in?

Please provide your current full mailing address below.

Street Address

Address Line 2

City

State / Province / Region

Postal / Zip Code

Country

_ Which of the following selections describes your ancestry? Please select all that apply.

_ American Indian or Alaska Native

_ Asian

_ Black or African American

_ Hispanic or Latino

_ Native Hawaiian or Other Pacific Islander

_ Caucasian, Non-Finnish Ancestry

_ Caucasian, Finnish Ancestry

QUORUM REVIEW
APPROVED
INSTITUTIONAL
REVIEW BOARD

_ I prefer not to say

_ Other (please specify)

Please enter your primary phone number where we may contact you if needed.

Please enter your email address.

--NEXT PAGE—

Would you consider yourself generally healthy?

_ Yes

_ No

Do you feel healthy today?

_ Yes

_ No

Please list any medical conditions or past diagnoses that we should be aware of or type in NA if this does not apply to you.

Please supply any reports or medical records associated with those diagnoses if you have any.

[FILE UPLOAD BUTTON]

Are you on any medications currently?

_ Yes

_ No

QUORUM REVIEW
APPROVED
INSTITUTIONAL
REVIEW BOARD

If yes, please list any drugs that you are currently taking.

--NEXT PAGE—

Please provide the current legal first name of the patient you are related to.

Please provide the current legal middle name of the patient you are related to. Please type in 0 if none.

Please provide the current legal last name of the patient you are related to.

How are you related to the patient? Patient is my _____.

_ Daughter

_ Granddaughter

_ Grandson

_ Half-brother

_ Half-sister

_ Maternal Aunt

_ Maternal Cousin

_ Maternal Grandfather

_ Maternal Grandmother

_ Maternal Uncle

_ Brother

_ Father

_ Mother

_ Sister

_ Nephew

QUORUM REVIEW
APPROVED
INSTITUTIONAL
REVIEW BOARD

_ Niece

_ Paternal Aunt

_ Paternal Cousin

_ Paternal Grandfather

_ Paternal Grandmother

_ Paternal Uncle

_ Son

_ Other

_ Don’t Know (please specify)

If Other, how are you related to the patient?

Have you experienced any of the following clinical features? Please select "None" if you have not experienced any.

_ None

Clinical features experienced by you:

_ Flu-like symptoms

_ Night sweats

_ Fevers (>100.5F)

_ Weight Loss

_ Fatigue

_ Large Spleen and/or liver

_ Edema/anasarca, effusions, ascites, or other form of fluid overload

_ Eruption of Cherry Hemangiomas on the skin or violaceous papules

_ Interstitial pneumonitis

QUORUM REVIEW
APPROVED
INSTITUTIONAL
REVIEW BOARD

Have you experienced any of the following laboratory abnormalities? Please select "None" if you have not experienced any.

_ None

Laboratory abnormalities you experienced:

_ Elevated C-Reactive Protein (CRP) (>10mg/dL)

_ Elevated Erythrocyte Sedimentation Rate (ESR) (>15mm/hr)

_ Low Hemoglobin/Anemia (Hgc<12.5 for males, <11.5 for females)

_ Elevated Platelets/Thrombocytosis (>400k/uL)

_ Low Platelets/Thrombocytopenia (<150K/uL)

_ Low Albumin/Hypoalbuminemia (<3.5 gm/dL)

_ Kidney dysfunction (elevated Creatinine or BUN) or proteinuria (protein in urine)

_ High Gamma Globin

_ Levels/Hypergammaglobulinemia (>1700mg/dL)

--NEXT PAGE—

Thank you for completing our survey! We really appreciate your support in fighting back against Castleman disease.

The CDCN will review the information you have provided and determine how you can be involved in providing samples for research. We will be in touch once we have gathered enough information to be able to send you a kit in the mail with empty tubes or if we have any questions for you.

QUORUM REVIEW
APPROVED
INSTITUTIONAL
REVIEW BOARD

Healthy volunteer (> 18 years old)

Thank you for filling out this Survey, so that we will be able to include your samples in the BioBank.

If you are presented with a Survey Page that does not contain any questions, please select "Next Page" to continue through the Survey. That particular Survey Page does not contain any questions that pertain to you based on your prior responses.

If you have any questions, please contact Dustin Shilling at 1-215-614-0689
or dustin@castlemannetwork.org

The following form will enable you to participate in the CDCN Biobank (Castlebank). Tissue samples such as blood or excess biopsy samples from patients will enable scientists to better characterize and understand Castleman disease and develop better treatments. Any blood or tissue donation will be used for high-impact research to battle this disease!

Is this your first time filling out this Survey? (If you do not remember if you have participated in the past, please call 1-215-614-0689)

X First Time Participant

_ Repeat Participant

--NEXT PAGE--

Please select the appropriate category that describes the PERSON completing this survey. (This question refers to the person completing the survey, NOT necessarily the patient)

_ Adult CD patient (> 18 years old)

_ Parent of pediatric CD patient

_ Related family member of CD patient (without CD and > 18 and NOT parent of pediatric CD patient)

_ Patient with related disease (e.g. Lupus, Rheumatoid Arthritis and > 18 years old)

X Healthy volunteer (> 18 years old)

--NEXT PAGE--

QUORUM REVIEW
APPROVED
INSTITUTIONAL
REVIEW BOARD

RESEARCH CONSENT FORM – KEY POINTS

For Family Members, Healthy Controls, or Similar Disease Controls

Study Title: The Castleman Disease Collaborative Network Biobank: A Collection of Biospecimens and Clinical Data to Facilitate Research
Study #: 001
Sponsor: Castleman Disease Collaborative Network
Study Investigator: David Fajgenbaum
Castleman Disease Collaborative Network
1001 S. 18th St., Unit A., Philadelphia, PA 19146
Telephone Number: (610) 304-0696

The goal of the "Castleman Disease Collaborative Network Biobank" project is to be a patient-driven movement that empowers people with Castleman Disease to directly transform research and treatment of disease by providing blood, sharing part of their stored tissue, and copies of their medical records for use in a variety of research projects. Because we are enrolling people with Castleman across the country regardless of where they are being treated, this study will allow many more people to contribute to research than has previously been possible. We are also inviting family members of people with Castleman Disease, healthy individuals, as well as individuals who have diseases that share similar characteristics to Castleman Disease to use as comparators to patients with Castleman Disease.

1. What is the purpose of this study?

We want to understand Castleman Disease better so that we can develop more effective therapies. The Castleman Disease Collaborative Network would like to partner directly with people with Castleman Disease, so we can study many more aspects of Castleman Disease than would otherwise be possible.

2. What will I have to do if I agree to participate in this study?

You will answer some questions about your medical history and send a blood and/or saliva sample to us in a pre-stamped package that we will provide.

3. Do I have to participate in this study?

No. Taking part in this study is voluntary. Even if you decide to participate, you can always change your mind and leave the study.

QUORUM REVIEW
APPROVED
INSTITUTIONAL
REVIEW BOARD

4. Will I benefit from participating?

While taking part in this study will not improve your own health, the information we collect will aid in research efforts to provide better treatment and prevention options to people with Castleman in the future. We will provide updates about key research discoveries made possible by this biobank on our website, and directly to you via email if you so choose.

5. What are the risks of taking part in this research?

There may be a risk that your information (which includes your genetic information and medical history information) could be seen by unauthorized individuals. However, we have procedures and security measures in place designed to minimize this risk and protect the confidentiality of your information.

If you decide to give blood for this biobank, you may experience pain, bruising, dizziness, or an infection.

6. Will it cost me anything to participate in this study?

No.

7. Who will use my samples and see my information?

Your samples and health information will be available to researchers at the Castleman Disease Collaborative Network (CDCN), a not-for-profit global initiative dedicated to accelerating research and treatment for Castleman disease. Your samples may also be used by outside researchers, both in the U.S. and internationally. After removing your name and other readily identifiable information, we will share tissues with qualified researchers, who have undergone review and approval by a CDCN Advisory Board. Results from the research studies may be shared with the greater research community via publications, as well as central data banks at the National Institutes of Health.

8. Can I stop taking part in this research study?

Yes, you can withdraw from this research study at any time. CDCN will destroy all your samples in its possession; if samples and information have already been sent to researchers, the CDCN will notify the researcher of the participant’s wishes but the CDCN cannot guarantee these samples will be destroyed. In addition, research data may have already been published before you withdrew and that cannot be retracted. Your information would be removed from future studies.

9. What if I have questions?

If you have any questions, please contact the Principal Investigator, Dr. David Fajgenbaum at (215) 614-0936. In case you want to inquire via writing, their address is 1001 S. 18th St, A Philadelphia PA 19146.

QUORUM REVIEW
APPROVED
INSTITUTIONAL
REVIEW BOARD

FULL RESEARCH CONSENT FORM

For Family Members and Healthy Controls

The Castleman Disease Collaborative Network Biobank: A Collection of Biospecimens and Clinical Data to Facilitate Research

A. Introduction

You are being invited to participate in a research study that will establish a Castleman Disease biobank. A biobank, sometimes called a biorepository, collects, stores, analyzes and distributes samples of human tissues, blood, and related health information to qualified scientists. Creation of a biobank for this disease may help doctors and researchers better understand why Castleman Disease occurs and develop ways to better treat and prevent it. Because Castleman Disease is so rare, it has not been well studied, due in part to a lack of available biospecimens and data for research use.

You are being asked to participate in the study because you are either a family member of someone with Castleman Disease, or are a healthy individual. You are being asked to provide blood or saliva samples. In order to execute meaningful research, studies require a comparator in order to determine what makes people with the disease different. Family members and healthy individuals who serve as these comparators or “controls” provide information that can be used to compare to people with Castleman, to better understand the biology of Castleman Disease.

The biobank is being established by the Castleman Disease Collaborative Network (CDCN), a global initiative dedicated to accelerating research and treatment for Castleman Disease. We work to achieve this by facilitating collaboration among the global research community, mobilizing resources, strategically investing in high-impact research, and supporting people with Castleman Disease and their family members. This biobank is paid for by the CDCN.

This form describes the project in order to help you decide if you want to participate. This form will tell you what you will have to do and the risks and benefits of participating.

If you have any questions about or do not understand something in this form, you should ask the research staff. You should also discuss your participation with anyone you choose in order to better understand this project and your options. Do not sign this form unless the staff has answered your questions and you decide that you want to be part of this project.

When reading this form, please note that the words “you” and “your” refer to the person in the project.

B. What is the purpose of this biobank?

QUORUM REVIEW
APPROVED
INSTITUTIONAL
REVIEW BOARD

The Castleman Disease Collaborative Network is creating this biobank to support research. We want scientists to better understand Castleman Disease and work toward developing more effective therapies. We will be able to study many more aspects of Castleman than has previously been possible.

By participating in this biobank project, you will be asked to provide blood and/or saliva now or in the future as well as medical information to be used for research. We would also like your permission to access and share (after removing your name and other personal identifiers) your medical information along with your specimens.

Some of the research might involve studying DNA contained in your samples. DNA is the material that makes up genes. Genes determine things like hair color and eye color, and may play a role in Castleman Disease. Using DNA from your blood or tissue sample, researchers will study your entire genetic sequence, known as your genome, and store this information. Your genomic data and health information will be studied along with information from other participants in this study, and it will be stored for future studies.

Samples may be studied immediately or may be stored for future use by researchers from universities, the government, health-related companies, and other research institutes. Some researchers will be from the United States and some may be from other countries around the world. A Biobank Advisory Board (BAB) composed of physicians and scientists will review research requests to help ensure that tissues are being utilized in the most impactful manner. The BAB will not to attempt to re-identify you. Future researchers must also sign an agreement indicating they will not attempt to re-identify you. Because technology is changing all the time it is not possible to know all of the techniques that researchers may use now or in the future.

We are asking many people to participate in the biobank, both people with Castleman, their family members, and healthy individuals (as a comparison group). We don’t know how many people will participate.

C. What other options are there?

Your participation in the biobank project is voluntary. Your alternative is not to participate. The decision not to participate or to stop participating at any time will not involve any penalty to you and will not involve any loss of benefits to which you might otherwise be entitled.

D. What is involved in the biobank project?

With your consent, project staff will collect medical information through a secure questionnaire regarding your health, medical, and family history.

The types of samples the Castleman Disease Collaborative Network wants to collect from family members and health controls include:

- blood samples already stored, scheduled to be taken, or that may be taken in the future
- saliva

QUORUM REVIEW
APPROVED
INSTITUTIONAL
REVIEW BOARD

Each type of sample collection is optional. You can decide to participate in each type of sample collection separately, which means you can agree to some, all, or none of the different types of samples. You can indicate your choice at the end of this form.

If you consent to have blood drawn for the biobank, it may be collected at the same time as routine medical visits, if possible. The biobank will send you blood collection kits with tubes and instructions that you can take to your doctor or local laboratory so they can obtain one or more blood samples. The amount of blood collected will be no greater than 50 mL (about 3 tablespoons) over a 2-month period.

If you give your consent to be re-contacted, biobank staff may contact you periodically to update your medical information or ask you to provide new samples for research. You may also choose if you would like the Castleman Disease Collaborative Network to notify you about other research studies in the future.

People who work with the sponsor (CDCN) will store your samples in a secure laboratory. Your samples will be stored as long as the sponsor decides to keep them.

E. How long will I be in this research study?

Your direct involvement will be limited initially to the time it takes to obtain your consent and to collect samples and medical information. Because this project involves a biobank, your samples, data, and health information may be used immediately or may be stored indefinitely for future use. If you agree, we may also re-contact you to get updates about your health and medical care, to ask whether you will give additional samples, or to tell you about other studies.

If you want to stop being in the biobank project, contact the Principal Investigator or project staff using the contact information provided later in this form. If you decide to withdraw from the project early, the Principal Investigator or project staff may ask you some questions about being in the project to gain feedback on ways in which biobank may be improved. The Principal Investigator or the sponsor can remove you from the project at any time, even if you want to stay in the project.

F. What kind of information could be found in this study and will I be able to see it?

This research is being done to add to our knowledge of Castleman Disease, and is not intended to provide information about your individual health. Investigators do not plan on returning the research test results to you, and the results will not be placed in your regular medical record. However, if a research test reveals something that could potentially affect your medical care, they may contact your physician to have the tests confirmed in a clinical lab. Your physician, not the investigator, will then follow-up with you. We will obtain consent from you to provide your physician your research results and ask you for updated physician contact information at that time.

If you choose, the Castleman Disease Collaborative Network can also keep you up to date on the research outcomes of projects using samples or data from this biobank; however not all research projects will use samples from all the donors to the bank, so your own tissue may or may not have been part of a particular research project. You do not need to participate in this biobank to be kept up-to-date

QUORUM REVIEW
APPROVED
INSTITUTIONAL
REVIEW BOARD

on the Castleman Disease Collaborative Network's research efforts; instead, you can sign up online for our email list here at cdcn.org. We regularly update the website.

If you choose, your samples and information may also be used for other central databanks. To allow research collaborations and sharing of data, the National Institutes of Health (NIH) and other research organizations have developed special data (information) repositories that analyze data and collect the results of certain types of genetic studies. These central banks will store your genetic information and provide them to qualified researchers to do more studies. This information will be available for any research question, such as research to understand what causes certain diseases (for example heart disease, cancer, or psychiatric disorders), development of new scientific methods, or the study of where different groups of people may have come from. We are also asking your permission to share your results with these special controlled-access banks. Controlled-access means that only researchers who apply for and get permission to use the information for a specific research project will be able to access the information. Your genomic data and health information will not be labeled with your name or other information that could be used to identify you. Researchers approved to access information in the database will agree not to attempt to identify you. Your information will be sent only with a code number attached. All directly identifiable information, such as your name, will not be shared with data banks or other investigators. Although there may be a small risk of loss of privacy when sharing this information with these banks, we have established procedures to encode your samples and information and protect your data. Although we will do everything we can to protect the privacy of your data, we cannot absolutely guarantee its privacy or predict how genetic information will be used in the future.

Furthermore, we will publish important discoveries found through these studies in the scientific literature so that the entire research community can work together to better understand Castleman Disease. Your individual data will not be published in a way in which you could be readily identified. Abstracts, which are plain language summaries of the published reports, will be available to you and the general public.

G. What are the risks or discomforts of the biobank project?

Your regular medical care will not change if you participate in this biobank project. If you agree to give a blood sample, the medical staff will take your blood by sticking a needle in your arm. The amount of blood that will be collected will not exceed 50 mL (about 3 tablespoons) over a 2-month period. Some problems you might have from this are:

- It may hurt.
- You may get a bruise.
- You may feel dizzy.
- You may get an infection.

There are some risks related to the use of your medical information and specimens. There could be an accidental release of your medical information to people who are not involved in your medical care. An accidental release of your genetic information could be used to identify you and your family members. We have tried to minimize this risk by carefully limiting access to the computers that would house your

QUORUM REVIEW
APPROVED
INSTITUTIONAL
REVIEW BOARD

information and having other strict data security measures in place. There is still a risk someone could get access to the information we have stored about you, including information about a family member (for example, revealing that you or a blood relative carry a genetic disease). Because your DNA information is unique to you, there is a chance that someone could trace it back to you. The risk of this happening is small, but may be greater in the future.

Federal and State laws also provide some protections against discrimination based on genetic information. For example, the Genetic Information Nondiscrimination Act (GINA) makes it illegal for health insurance companies, group health plans, and most employers to discriminate against you based on your genetic information. However, it does not prevent companies that sell life insurance, disability insurance, or long-term care insurance from using genetic information as a reason to deny coverage or set premiums.

H. What are the benefits of the research study?

Research conducted with your information and specimens will not help you directly, but it may help people with Castleman in the future. Research performed with your information and specimens could result in the discovery and development of new drugs, tests or commercial products.

I. Can I stop being in the research study and what are my rights?

You can stop being in the research study at any time. You may choose to leave your samples and/or information in the biobank but request not to be re-contacted. Or, if you want to end all participation, the CDCN will destroy all of your sample identifiers and medical information in its possession; the CDCN will retain your original consent and the documentation of your withdrawal of consent. Any specimens or data that have already been distributed in a de-identified manner cannot be destroyed, and some research data may have already been published. If data has already been distributed to third parties, it cannot be retrieved (as it will have been de-identified). Any specimens or identifiable data you have given can be removed upon request, but any de-identified, already distributed information or specimens cannot be withdrawn.

To withdraw from research, you must notify, in writing, the Principal Investigator (Address: 1001 S. 18th St, A Philadelphia PA 19146). There are three ways you can choose to stop participating in the Biobank:

1. You may choose to leave your data and unused samples in the Biobank while requesting not to be re-contacted in the future.

2. You may request that your personally identifying information be destroyed, but allow some or all of your samples to continue being used anonymously for future research.

3. You can choose to end all participation, in which case all of the unused samples in the Biobank will be destroyed and your medical information will be deleted.

J. Will I be paid to take part in this research study?

QUORUM REVIEW
APPROVED
INSTITUTIONAL
REVIEW BOARD

You will not get payment for being in this biobank project. The sponsor does not plan to give you any money or payment resulting from any discoveries or products made from this research project.

K. What are the costs?

Participating in the biobank project should not cost you anything beyond your routine, scheduled medical costs. If you decide to donate saliva and blood samples, we recommend you get these drawn during your regularly scheduled clinical appointments. You and your insurance company will already be paying for the costs of the blood draws as a part of your standard medical care. That way there's no additional cost. If you don't have regularly scheduled follow-up but still want to participate, please visit an outpatient clinic to get your blood drawn. You will need to pay for the cost of the blood draw but the CDCN can reimburse you if you fill out a W2 form and provide a copy of the original receipt. Neither you nor your insurance company will be asked to pay for research tests.

L. What happens if I am injured or sick because I took part in this research study?

If injury (physical, psychological, social, financial, or otherwise) occurs as a result of participation in this project, there are no plans for providing compensation to you or your family.

Be aware that your health care payer/insurer might not cover the costs of study-related injuries.

You do not give up any of your legal rights by signing this form.

M. What about confidentiality?

Your individual data and health information (without your name, address, or other details that might identify you) will be put in a controlled-access database. This means that only researchers who apply for and get permission to use the information for a specific research project will be able to access the information. Researchers approved to access information in the database will agree not to attempt to identify you. Access to information with your name, such as the consent form, will be restricted to healthcare staff, the Principal Investigator, designated CDCN staff, and approved CDCN contractors. Documents will be kept in a locked cabinet and information in computers will be protected by passwords. In order to protect your private information, only a unique code number, not your name or medical record number, will appear on your samples or on information sent to researchers outside the CDCN. Some people who work for the biobank will be able to find your name from the code so they can possibly have your sample(s) destroyed if you change your mind later. Except when required by law, your personally identifying information will not be disclosed outside of the Castleman Disease Collaborative Network.

N. Whom do I contact if I have questions about the research study?

If you have any questions about the study, please contact the research doctor or study staff listed below by email davidfajgenbaum@gmail.com or calling 215-614-0936:

QUORUM REVIEW
APPROVED
INSTITUTIONAL
REVIEW BOARD

- David Fajgenbaum, MD (Principal Investigator)

If you prefer to do so in writing, please send written correspondence to the address 1001 S. 18th St, A Philadelphia PA 19146.

You can learn more about the Castleman Disease Collaborative Network by visiting our website at www.cdcn.org.

Quorum Review Institutional Review Board (IRB) has reviewed this project. An IRB is a group of people who review research studies to protect the rights and welfare of research participants. If you have questions about your rights as a research participant, if you are not able to resolve your concerns with the Principal Investigator, if you have a complaint, or if you have general questions about what it means to be in a research project, you can call Quorum Review or visit the Quorum Review website at www.quorumreview.com.

Quorum Review is located in Seattle, Washington.

Office hours are 8:00 AM to 5:00 PM Pacific Time, Monday through Friday.

Ask to speak with a Research Participant Liaison at 888-776-9115 (toll free).

O. Conflict of Interest

Dr. David Fajgenbaum, the person responsible for the conduct of this research study also serves as the Executive Director of the CDCN. You should be aware that you are not under any obligation to participate in this research study.

P. Authorization to use your health information for research purposes

Because information about you and your health is personal and private, it generally cannot be used in this research study without your written authorization. This section is intended to inform you about how your health information will be used or disclosed in the study. Your information will only be used as described here and as required or allowed by law.

If you do not want to allow the uses described here, you cannot be in this biobank. You can still be in the biobank even if you do not give permission for the use and sharing of your information for the optional parts of the biobank.

1. What personal information about me will be used or shared with others during this research?

- Your tissue samples relevant to this research study and related records
- New health information created from study-related tests and/or questionnaires

2. Why will protected information about me be used or shared with others?

QUORUM REVIEW
APPROVED
INSTITUTIONAL
REVIEW BOARD

The main reasons include the following:

- To conduct and oversee the research described earlier in this form;
- To ensure the research meets legal, institutional, and accreditation requirements;
- To conduct public health activities (including reporting of adverse events or situations where you or others may be at risk of harm)

3. Who will use or share protected health information about me?

With your consent, biobank staff will obtain information about your health from you, and they may share your consent information in connection with the research study.

4. With whom outside of the Castleman Disease Collaborative Network may my personal health information be shared?

While all reasonable efforts will be made to protect the confidentiality of your protected health information, it may also be shared with the following entities:

- Federal and state agencies (for example, the Department of Health and Human Services, the Food and Drug Administration, the National Institutes of Health, and/or the Office for Human Research Protections), or other domestic or foreign government bodies if required by law and/or necessary for oversight purposes. A qualified representative of the FDA, the National Cancer Institute, and Quorum Review may review your medical records.
- Outside individuals or entities that have a need to access this information to perform functions relating to the conduct of this research such as data storage companies.

5. For how long will protected health information about me be used or shared with others?

There is no scheduled date at which your protected health information that is being used or shared for this research will be destroyed, because research is an ongoing process.

6. Statement of privacy rights:

- You have the right to withdraw your permission for the doctors and researchers to use or share your protected health information. We will not be able to withdraw all the information that already has been used or shared with others to carry out related activities such as oversight, or that is needed to ensure quality of the study. To withdraw your permission, you must do so in writing by contacting the researcher listed above in the section: "Whom do I contact if I have questions about the research study?" If you do so, you will no longer be a participant in this study. You can withdraw your permission for the optional parts of the biobank and still be in the biobank.

QUORUM REVIEW
APPROVED
INSTITUTIONAL
REVIEW BOARD

•	After your information is shared with the Network, federal law will no longer require it be protected. The Network may also share your information with other people who the federal law does not apply too. The Network will nonetheless only use or disclose your information as described in this form and will have its own security measures in place to ensure your information and privacy remains protected.

Q. Consent

I have read this form, and I have been able to ask questions about this study. I understand that my participation is voluntary, and if I decide not to participate it will have no impact on my medical care. If decide to participate now, I can decide to stop being in the study at any time. I voluntarily agree to take part in this research biobank. By signing this form, I do not give up any of my legal rights.

I Consent

_ Yes I Consent

REQUIRED ACTIVITIES:

<u>I agree to the following activities as part of this biobank. (You must agree to all of the following to participate in the biobank. If you do not agree to all of these, you should not join the biobank.)</u>

I am willing to provide any <u>future specimens</u> to the biobank. (Even if you check this now, you can change your mind in the future.)

_ Yes I Agree

You can use the results of the research on my samples and my medical information <u>for future research studies</u>, including studies that have not yet been designed, studies for diseases other than Castleman disease, and/or studies that may be for commercial purposes.

_ Yes I Agree

You can share the results of the research on my samples and my medical information with <u>central data banks</u> (e.g., the NIH) and with other qualified researchers. It will be shared in a manner that does not include my name, social security number, or any other information that could be used to readily identify me, to be used by other qualified researchers to perform future research studies, including studies that have not yet been designed, studies for diseases other than Castleman disease, and studies that may be for commercial purposes.

_ Yes I Agree

QUORUM REVIEW
APPROVED
INSTITUTIONAL
REVIEW BOARD

OPTIONAL ACTIVITIES:

Please check below if you are willing to allow any of these optional activities as part of this research biobank. You can still be in the study even if you do not agree to any of these activities:

I am willing to have blood drawn for the biobank. You can perform (or collaborate with others to perform) research on the blood sample that I will send you and store the sample until this research study is complete.

_ Yes I Agree

I am willing to have my saliva collected for the biobank. You can perform (or collaborate with others to perform) research on the saliva sample that I will send you and store the sample until this research study is complete.

_ Yes I Agree

I am willing to be re-contacted by study staff later, to update my medical information or provide new samples for research. Even if I agree now by checking this box, I can decide not to provide this information or additional samples when I am re-contacted.

_ Yes I Agree

I am willing to be re-contacted to obtain my consent in the event that return of my personal research results becomes possible.

_ Yes I Agree

You can request my stored tissue samples (blood and/or tissue (lymph nodes, bone marrow)) from my physicians and the hospitals and other places where I received my care, perform (or collaborate with others to perform) research on the samples, and store the samples until this research study is complete.

_ Yes I Agree

You should print a copy of this form for your records.

Signature of Participant

QUORUM REVIEW
APPROVED
INSTITUTIONAL
REVIEW BOARD

First

Last

Date of Participant Signature

MM/DD/YYYY

--NEXT PAGE—

If you selected “Parent of pediatric CD patient,” at the beginning of the survey please respond on behalf of the PATIENT

If you selected “Adult CD patient”/ “Related family member of CD patient”/ “Patient with related disease”/ “Healthy volunteer,” at the beginning of this survey, please enter YOUR information.

Please provide your current legal first name.

Please provide your current legal middle name. Please type in 0 if none.

Please provide your current legal last name.

Has your name changed since birth?

_ Yes

_ No

What is your date of birth?

MM/DD/YYYY

What was your physical sex at birth?

_ Male

_Female

QUORUM REVIEW
APPROVED
INSTITUTIONAL
REVIEW BOARD

What city/municipality were you born in?

What country were you born in?

Please provide your current full mailing address below.

Street Address

Address Line 2

City

State / Province / Region

Postal / Zip Code

Country

_ Which of the following selections describes your ancestry? Please select all that apply.

_ American Indian or Alaska Native

_ Asian

_ Black or African American

_ Hispanic or Latino

_ Native Hawaiian or Other Pacific Islander

_ Caucasian, Non-Finnish Ancestry

_ Caucasian, Finnish Ancestry

_ I prefer not to say

_ Other (please specify)

QUORUM REVIEW
APPROVED
INSTITUTIONAL
REVIEW BOARD

Please enter your primary phone number where we may contact you if needed.

Please enter your email address.

--NEXT PAGE—

Would you consider yourself generally healthy?

_ Yes

_ No

Do you feel healthy today?

_ Yes

_ No

Please list any medical conditions or past diagnoses that we should be aware of or type in NA if this does not apply to you.

Please supply any reports or medical records associated with those diagnoses if you have any.

[FILE UPLOAD BUTTON]

Are you on any medications currently?

_ Yes

_ No

If yes, please list any drugs that you are currently taking.

QUORUM REVIEW
APPROVED
INSTITUTIONAL
REVIEW BOARD

--NEXT PAGE—

Thank you for completing our survey! We really appreciate your support in fighting back against Castleman disease.

The CDCN will review the information you have provided and determine how you can be involved in providing samples for research. We will be in touch once we have gathered enough information to be able to send you a kit in the mail with empty tubes or if we have any questions for you.

QUORUM REVIEW
APPROVED
INSTITUTIONAL
REVIEW BOARD

Patient with related disease (e.g. Lupus, Rheumatoid Arthritis and > 18 years old)

Thank you for filling out this Survey, so that we will be able to include your samples in the BioBank.

If you are presented with a Survey Page that does not contain any questions, please select "Next Page" to continue through the Survey. That particular Survey Page does not contain any questions that pertain to you based on your prior responses.

If you have any questions, please contact Dustin Shilling at 1-215-614-0689 or dustin@castlemannetwork.org

The following form will enable you to participate in the CDCN Biobank (Castlebank). Tissue samples such as blood or excess biopsy samples from patients will enable scientists to better characterize and understand Castleman disease and develop better treatments. Any blood or tissue donation will be used for high-impact research to battle this disease!

Is this your first time filling out this Survey? (If you do not remember if you have participated in the past, please call 1-215-614-0689)

X First Time Participant

_ Repeat Participant

--NEXT PAGE--

Please select the appropriate category that describes the PERSON completing this survey. (This question refers to the person completing the survey, NOT necessarily the patient)

_ Adult CD patient (> 18 years old)

_ Parent of pediatric CD patient

_ Related family member of CD patient (without CD and > 18 and NOT parent of pediatric CD patient)

X Patient with related disease (e.g. Lupus, Rheumatoid Arthritis and > 18 years old)

_ Healthy volunteer (> 18 years old)

--NEXT PAGE--

QUORUM REVIEW
APPROVED
INSTITUTIONAL
REVIEW BOARD

RESEARCH CONSENT FORM – KEY POINTS

For Family Members, Healthy Controls, or Similar Disease Controls

Study Title: The Castleman Disease Collaborative Network Biobank: A Collection of Biospecimens and Clinical Data to Facilitate Research
Study #: 001
Sponsor: Castleman Disease Collaborative Network
Study Investigator: David Fajgenbaum
Castleman Disease Collaborative Network
1001 S. 18th St., Unit A., Philadelphia, PA 19146
Telephone Number: (610) 304-0696

The goal of the "Castleman Disease Collaborative Network Biobank" project is to be a patient-driven movement that empowers people with Castleman Disease to directly transform research and treatment of disease by providing blood, sharing part of their stored tissue, and copies of their medical records for use in a variety of research projects. Because we are enrolling people with Castleman across the country regardless of where they are being treated, this study will allow many more people to contribute to research than has previously been possible. We are also inviting family members of people with Castleman Disease, healthy individuals, as well as individuals who have diseases that share similar characteristics to Castleman Disease to use as comparators to patients with Castleman Disease.

1. What is the purpose of this study?

We want to understand Castleman Disease better so that we can develop more effective therapies. The Castleman Disease Collaborative Network would like to partner directly with people with Castleman Disease, so we can study many more aspects of Castleman Disease than would otherwise be possible.

2. What will I have to do if I agree to participate in this study?

You will answer some questions about your medical history and send a blood and/or saliva sample to us in a pre-stamped package that we will provide.

3. Do I have to participate in this study?

No. Taking part in this study is voluntary. Even if you decide to participate, you can always change your mind and leave the study.

QUORUM REVIEW
APPROVED
INSTITUTIONAL
REVIEW BOARD

4. Will I benefit from participating?

While taking part in this study will not improve your own health, the information we collect will aid in research efforts to provide better treatment and prevention options to people with Castleman in the future. We will provide updates about key research discoveries made possible by this biobank on our website, and directly to you via email if you so choose.

5. What are the risks of taking part in this research?

There may be a risk that your information (which includes your genetic information and medical history information) could be seen by unauthorized individuals. However, we have procedures and security measures in place designed to minimize this risk and protect the confidentiality of your information.

If you decide to give blood for this biobank, you may experience pain, bruising, dizziness, or an infection.

6. Will it cost me anything to participate in this study?

No.

7. Who will use my samples and see my information?

Your samples and health information will be available to researchers at the Castleman Disease Collaborative Network (CDCN), a not-for-profit global initiative dedicated to accelerating research and treatment for Castleman disease. Your samples may also be used by outside researchers, both in the U.S. and internationally. After removing your name and other readily identifiable information, we will share tissues with qualified researchers, who have undergone review and approval by a CDCN Advisory Board. Results from the research studies may be shared with the greater research community via publications, as well as central data banks at the National Institutes of Health.

8. Can I stop taking part in this research study?

Yes, you can withdraw from this research study at any time. CDCN will destroy all your samples in its possession; if samples and information have already been sent to researchers, the CDCN will notify the researcher of the participant’s wishes but the CDCN cannot guarantee these samples will be destroyed. In addition, research data may have already been published before you withdrew and that cannot be retracted. Your information would be removed from future studies.

9. What if I have questions?

QUORUM REVIEW
APPROVED
INSTITUTIONAL
REVIEW BOARD

If you have any questions, please contact the Principal Investigator, Dr. David Fajgenbaum at (215) 614-0936. In case you want to inquire via writing, their address is 1001 S. 18th St, A Philadelphia PA 19146.

FULL RESEARCH CONSENT FORM

For Family Members and Healthy Controls

The Castleman Disease Collaborative Network Biobank: A Collection of Biospecimens and Clinical Data to Facilitate Research

A. Introduction

You are being invited to participate in a research study that will establish a Castleman Disease biobank. A biobank, sometimes called a biorepository, collects, stores, analyzes and distributes samples of human tissues, blood, and related health information to qualified scientists. Creation of a biobank for this disease may help doctors and researchers better understand why Castleman Disease occurs and develop ways to better treat and prevent it. Because Castleman Disease is so rare, it has not been well studied, due in part to a lack of available biospecimens and data for research use.

You are being asked to participate in the study because you are either a family member of someone with Castleman Disease, or are a healthy individual. You are being asked to provide blood or saliva samples. In order to execute meaningful research, studies require a comparator in order to determine what makes people with the disease different. Family members and healthy individuals who serve as these comparators or “controls” provide information that can be used to compare to people with Castleman, to better understand the biology of Castleman Disease.

The biobank is being established by the Castleman Disease Collaborative Network (CDCN), a global initiative dedicated to accelerating research and treatment for Castleman Disease. We work to achieve this by facilitating collaboration among the global research community, mobilizing resources, strategically investing in high-impact research, and supporting people with Castleman Disease and their family members. This biobank is paid for by the CDCN.

This form describes the project in order to help you decide if you want to participate. This form will tell you what you will have to do and the risks and benefits of participating.

If you have any questions about or do not understand something in this form, you should ask the research staff. You should also discuss your participation with anyone you choose in order to better understand this project and your options. Do not sign this form unless the staff has answered your questions and you decide that you want to be part of this project.

When reading this form, please note that the words “you” and “your” refer to the person in the project.

B. What is the purpose of this biobank?

QUORUM REVIEW
APPROVED
INSTITUTIONAL
REVIEW BOARD

The Castleman Disease Collaborative Network is creating this biobank to support research. We want scientists to better understand Castleman Disease and work toward developing more effective therapies. We will be able to study many more aspects of Castleman than has previously been possible.

By participating in this biobank project, you will be asked to provide blood and/or saliva now or in the future as well as medical information to be used for research. We would also like your permission to access and share (after removing your name and other personal identifiers) your medical information along with your specimens.

Some of the research might involve studying DNA contained in your samples. DNA is the material that makes up genes. Genes determine things like hair color and eye color, and may play a role in Castleman Disease. Using DNA from your blood or tissue sample, researchers will study your entire genetic sequence, known as your genome, and store this information. Your genomic data and health information will be studied along with information from other participants in this study, and it will be stored for future studies.

Samples may be studied immediately or may be stored for future use by researchers from universities, the government, health-related companies, and other research institutes. Some researchers will be from the United States and some may be from other countries around the world. A Biobank Advisory Board (BAB) composed of physicians and scientists will review research requests to help ensure that tissues are being utilized in the most impactful manner. The BAB will not to attempt to re-identify you. Future researchers must also sign an agreement indicating they will not attempt to re-identify you. Because technology is changing all the time it is not possible to know all of the techniques that researchers may use now or in the future.

We are asking many people to participate in the biobank, both people with Castleman, their family members, and healthy individuals (as a comparison group). We don’t know how many people will participate.

C. What other options are there?

Your participation in the biobank project is voluntary. Your alternative is not to participate. The decision not to participate or to stop participating at any time will not involve any penalty to you and will not involve any loss of benefits to which you might otherwise be entitled.

D. What is involved in the biobank project?

With your consent, project staff will collect medical information through a secure questionnaire regarding your health, medical, and family history.

The types of samples the Castleman Disease Collaborative Network wants to collect from family members and health controls include:

- blood samples already stored, scheduled to be taken, or that may be taken in the future
- saliva

QUORUM REVIEW
APPROVED
INSTITUTIONAL
REVIEW BOARD

Each type of sample collection is optional. You can decide to participate in each type of sample collection separately, which means you can agree to some, all, or none of the different types of samples. You can indicate your choice at the end of this form.

If you consent to have blood drawn for the biobank, it may be collected at the same time as routine medical visits, if possible. The biobank will send you blood collection kits with tubes and instructions that you can take to your doctor or local laboratory so they can obtain one or more blood samples. The amount of blood collected will be no greater than 50 mL (about 3 tablespoons) over a 2-month period.

If you give your consent to be re-contacted, biobank staff may contact you periodically to update your medical information or ask you to provide new samples for research. You may also choose if you would like the Castleman Disease Collaborative Network to notify you about other research studies in the future.

People who work with the sponsor (CDCN) will store your samples in a secure laboratory. Your samples will be stored as long as the sponsor decides to keep them.

E. How long will I be in this research study?

Your direct involvement will be limited initially to the time it takes to obtain your consent and to collect samples and medical information. Because this project involves a biobank, your samples, data, and health information may be used immediately or may be stored indefinitely for future use. If you agree, we may also re-contact you to get updates about your health and medical care, to ask whether you will give additional samples, or to tell you about other studies.

If you want to stop being in the biobank project, contact the Principal Investigator or project staff using the contact information provided later in this form. If you decide to withdraw from the project early, the Principal Investigator or project staff may ask you some questions about being in the project to gain feedback on ways in which biobank may be improved. The Principal Investigator or the sponsor can remove you from the project at any time, even if you want to stay in the project.

F. What kind of information could be found in this study and will I be able to see it?

This research is being done to add to our knowledge of Castleman Disease, and is not intended to provide information about your individual health. Investigators do not plan on returning the research test results to you, and the results will not be placed in your regular medical record. However, if a research test reveals something that could potentially affect your medical care, they may contact your physician to have the tests confirmed in a clinical lab. Your physician, not the investigator, will then follow-up with you. We will obtain consent from you to provide your physician your research results and ask you for updated physician contact information at that time.

If you choose, the Castleman Disease Collaborative Network can also keep you up to date on the research outcomes of projects using samples or data from this biobank; however not all research projects will use samples from all the donors to the bank, so your own tissue may or may not have been part of a particular research project. You do not need to participate in this biobank to be kept up-to-date

QUORUM REVIEW
APPROVED
INSTITUTIONAL
REVIEW BOARD

on the Castleman Disease Collaborative Network's research efforts; instead, you can sign up online for our email list here at cdcn.org. We regularly update the website.

If you choose, your samples and information may also be used for other central databanks. To allow research collaborations and sharing of data, the National Institutes of Health (NIH) and other research organizations have developed special data (information) repositories that analyze data and collect the results of certain types of genetic studies. These central banks will store your genetic information and provide them to qualified researchers to do more studies. This information will be available for any research question, such as research to understand what causes certain diseases (for example heart disease, cancer, or psychiatric disorders), development of new scientific methods, or the study of where different groups of people may have come from. We are also asking your permission to share your results with these special controlled-access banks. Controlled-access means that only researchers who apply for and get permission to use the information for a specific research project will be able to access the information. Your genomic data and health information will not be labeled with your name or other information that could be used to identify you. Researchers approved to access information in the database will agree not to attempt to identify you. Your information will be sent only with a code number attached. All directly identifiable information, such as your name, will not be shared with data banks or other investigators. Although there may be a small risk of loss of privacy when sharing this information with these banks, we have established procedures to encode your samples and information and protect your data. Although we will do everything we can to protect the privacy of your data, we cannot absolutely guarantee its privacy or predict how genetic information will be used in the future.

Furthermore, we will publish important discoveries found through these studies in the scientific literature so that the entire research community can work together to better understand Castleman Disease. Your individual data will not be published in a way in which you could be readily identified. Abstracts, which are plain language summaries of the published reports, will be available to you and the general public.

G. What are the risks or discomforts of the biobank project?

Your regular medical care will not change if you participate in this biobank project. If you agree to give a blood sample, the medical staff will take your blood by sticking a needle in your arm. The amount of blood that will be collected will not exceed 50 mL (about 3 tablespoons) over a 2-month period. Some problems you might have from this are:

- It may hurt.
- You may get a bruise.
- You may feel dizzy.
- You may get an infection.

There are some risks related to the use of your medical information and specimens. There could be an accidental release of your medical information to people who are not involved in your medical care. An accidental release of your genetic information could be used to identify you and your family members. We have tried to minimize this risk by carefully limiting access to the computers that would house your

QUORUM REVIEW
APPROVED
INSTITUTIONAL
REVIEW BOARD

information and having other strict data security measures in place. There is still a risk someone could get access to the information we have stored about you, including information about a family member (for example, revealing that you or a blood relative carry a genetic disease). Because your DNA information is unique to you, there is a chance that someone could trace it back to you. The risk of this happening is small, but may be greater in the future.

Federal and State laws also provide some protections against discrimination based on genetic information. For example, the Genetic Information Nondiscrimination Act (GINA) makes it illegal for health insurance companies, group health plans, and most employers to discriminate against you based on your genetic information. However, it does not prevent companies that sell life insurance, disability insurance, or long-term care insurance from using genetic information as a reason to deny coverage or set premiums.

H. What are the benefits of the research study?

Research conducted with your information and specimens will not help you directly, but it may help people with Castleman in the future. Research performed with your information and specimens could result in the discovery and development of new drugs, tests or commercial products.

I. Can I stop being in the research study and what are my rights?

You can stop being in the research study at any time. You may choose to leave your samples and/or information in the biobank but request not to be re-contacted. Or, if you want to end all participation, the CDCN will destroy all of your sample identifiers and medical information in its possession; the CDCN will retain your original consent and the documentation of your withdrawal of consent. Any specimens or data that have already been distributed in a de-identified manner cannot be destroyed, and some research data may have already been published. If data has already been distributed to third parties, it cannot be retrieved (as it will have been de-identified). Any specimens or identifiable data you have given can be removed upon request, but any de-identified, already distributed information or specimens cannot be withdrawn.

To withdraw from research, you must notify, in writing, the Principal Investigator (Address: 1001 S. 18th St, A Philadelphia PA 19146). There are three ways you can choose to stop participating in the Biobank:

1. You may choose to leave your data and unused samples in the Biobank while requesting not to be re-contacted in the future.

2. You may request that your personally identifying information be destroyed, but allow some or all of your samples to continue being used anonymously for future research.

3. You can choose to end all participation, in which case all of the unused samples in the Biobank will be destroyed and your medical information will be deleted.

J. Will I be paid to take part in this research study?

QUORUM REVIEW
APPROVED
INSTITUTIONAL
REVIEW BOARD

You will not get payment for being in this biobank project. The sponsor does not plan to give you any money or payment resulting from any discoveries or products made from this research project.

K. What are the costs?

Participating in the biobank project should not cost you anything beyond your routine, scheduled medical costs. If you decide to donate saliva and blood samples, we recommend you get these drawn during your regularly scheduled clinical appointments. You and your insurance company will already be paying for the costs of the blood draws as a part of your standard medical care. That way there's no additional cost. If you don't have regularly scheduled follow-up but still want to participate, please visit an outpatient clinic to get your blood drawn. You will need to pay for the cost of the blood draw but the CDCN can reimburse you if you fill out a W2 form and provide a copy of the original receipt. Neither you nor your insurance company will be asked to pay for research tests.

L. What happens if I am injured or sick because I took part in this research study?

If injury (physical, psychological, social, financial, or otherwise) occurs as a result of participation in this project, there are no plans for providing compensation to you or your family.

Be aware that your health care payer/insurer might not cover the costs of study-related injuries.

You do not give up any of your legal rights by signing this form.

M. What about confidentiality?

Your individual data and health information (without your name, address, or other details that might identify you) will be put in a controlled-access database. This means that only researchers who apply for and get permission to use the information for a specific research project will be able to access the information. Researchers approved to access information in the database will agree not to attempt to identify you. Access to information with your name, such as the consent form, will be restricted to healthcare staff, the Principal Investigator, designated CDCN staff, and approved CDCN contractors. Documents will be kept in a locked cabinet and information in computers will be protected by passwords. In order to protect your private information, only a unique code number, not your name or medical record number, will appear on your samples or on information sent to researchers outside the CDCN. Some people who work for the biobank will be able to find your name from the code so they can possibly have your sample(s) destroyed if you change your mind later. Except when required by law, your personally identifying information will not be disclosed outside of the Castleman Disease Collaborative Network.

N. Whom do I contact if I have questions about the research study?

If you have any questions about the study, please contact the research doctor or study staff listed below by email davidfajgenbaum@gmail.com or calling 215-614-0936:

QUORUM REVIEW
APPROVED
INSTITUTIONAL
REVIEW BOARD

- David Fajgenbaum, MD (Principal Investigator)

If you prefer to do so in writing, please send written correspondence to the address 1001 S. 18th St, A Philadelphia PA 19146.

You can learn more about the Castleman Disease Collaborative Network by visiting our website at www.cdcn.org.

Quorum Review Institutional Review Board (IRB) has reviewed this project. An IRB is a group of people who review research studies to protect the rights and welfare of research participants. If you have questions about your rights as a research participant, if you are not able to resolve your concerns with the Principal Investigator, if you have a complaint, or if you have general questions about what it means to be in a research project, you can call Quorum Review or visit the Quorum Review website at www.quorumreview.com.

Quorum Review is located in Seattle, Washington.

Office hours are 8:00 AM to 5:00 PM Pacific Time, Monday through Friday.

Ask to speak with a Research Participant Liaison at 888-776-9115 (toll free).

O. Conflict of Interest

Dr. David Fajgenbaum, the person responsible for the conduct of this research study also serves as the Executive Director of the CDCN. You should be aware that you are not under any obligation to participate in this research study.

P. Authorization to use your health information for research purposes

Because information about you and your health is personal and private, it generally cannot be used in this research study without your written authorization. This section is intended to inform you about how your health information will be used or disclosed in the study. Your information will only be used as described here and as required or allowed by law.

If you do not want to allow the uses described here, you cannot be in this biobank. You can still be in the biobank even if you do not give permission for the use and sharing of your information for the optional parts of the biobank.

1. What personal information about me will be used or shared with others during this research?

- Your tissue samples relevant to this research study and related records
- New health information created from study-related tests and/or questionnaires

2. Why will protected information about me be used or shared with others?

QUORUM REVIEW
APPROVED
INSTITUTIONAL
REVIEW BOARD

The main reasons include the following:

- To conduct and oversee the research described earlier in this form;
- To ensure the research meets legal, institutional, and accreditation requirements;
- To conduct public health activities (including reporting of adverse events or situations where you or others may be at risk of harm)

3. Who will use or share protected health information about me?

With your consent, biobank staff will obtain information about your health from you, and they may share your consent information in connection with the research study.

4. With whom outside of the Castleman Disease Collaborative Network may my personal health information be shared?

While all reasonable efforts will be made to protect the confidentiality of your protected health information, it may also be shared with the following entities:

- Federal and state agencies (for example, the Department of Health and Human Services, the Food and Drug Administration, the National Institutes of Health, and/or the Office for Human Research Protections), or other domestic or foreign government bodies if required by law and/or necessary for oversight purposes. A qualified representative of the FDA, the National Cancer Institute, and Quorum Review may review your medical records.
- Outside individuals or entities that have a need to access this information to perform functions relating to the conduct of this research such as data storage companies.

5. For how long will protected health information about me be used or shared with others?

There is no scheduled date at which your protected health information that is being used or shared for this research will be destroyed, because research is an ongoing process.

6. Statement of privacy rights:

- You have the right to withdraw your permission for the doctors and researchers to use or share your protected health information. We will not be able to withdraw all the information that already has been used or shared with others to carry out related activities such as oversight, or that is needed to ensure quality of the study. To withdraw your permission, you must do so in writing by contacting the researcher listed above in the section: “Whom do I contact if I have questions about the research study?” If you do so, you will no longer be a participant in this study. You can withdraw your permission for the optional parts of the biobank and still be in the biobank.

QUORUM REVIEW
APPROVED
INSTITUTIONAL
REVIEW BOARD

• After your information is shared with the Network, federal law will no longer require it be protected. The Network may also share your information with other people who the federal law does not apply too. The Network will nonetheless only use or disclose your information as described in this form and will have its own security measures in place to ensure your information and privacy remains protected.

Q. Consent

I have read this form, and I have been able to ask questions about this study. I understand that my participation is voluntary, and if I decide not to participate it will have no impact on my medical care. If decide to participate now, I can decide to stop being in the study at any time. I voluntarily agree to take part in this research biobank. By signing this form, I do not give up any of my legal rights.

I Consent

_ Yes I Consent

REQUIRED ACTIVITIES:

I agree to the following activities as part of this biobank. (You must agree to all of the following to participate in the biobank. If you do not agree to all of these, you should not join the biobank.)

I am willing to provide any future specimens to the biobank. (Even if you check this now, you can change your mind in the future.)

_ Yes I Agree

You can use the results of the research on my samples and my medical information for future research studies, including studies that have not yet been designed, studies for diseases other than Castleman disease, and/or studies that may be for commercial purposes.

_ Yes I Agree

You can share the results of the research on my samples and my medical information with central data banks (e.g., the NIH) and with other qualified researchers. It will be shared in a manner that does not include my name, social security number, or any other information that could be used to readily identify me, to be used by other qualified researchers to perform future research studies, including studies that have not yet been designed, studies for diseases other than Castleman disease, and studies that may be for commercial purposes.

_ Yes I Agree

QUORUM REVIEW
APPROVED
INSTITUTIONAL
REVIEW BOARD

OPTIONAL ACTIVITIES:

Please check below if you are willing to allow any of these optional activities as part of this research biobank. You can still be in the study even if you do not agree to any of these activities:

I am willing to have blood drawn for the biobank. You can perform (or collaborate with others to perform) research on the blood sample that I will send you and store the sample until this research study is complete.

_ Yes I Agree

I am willing to have my saliva collected for the biobank. You can perform (or collaborate with others to perform) research on the saliva sample that I will send you and store the sample until this research study is complete.

_ Yes I Agree

I am willing to be re-contacted by study staff later, to update my medical information or provide new samples for research. Even if I agree now by checking this box, I can decide not to provide this information or additional samples when I am re-contacted.

_ Yes I Agree

I am willing to be re-contacted to obtain my consent in the event that return of my personal research results becomes possible.

_ Yes I Agree

You can request my stored tissue samples (blood and/or tissue (lymph nodes, bone marrow)) from my physicians and the hospitals and other places where I received my care, perform (or collaborate with others to perform) research on the samples, and store the samples until this research study is complete.

_ Yes I Agree

You should print a copy of this form for your records.

Signature of Participant

QUORUM REVIEW
APPROVED
INSTITUTIONAL
REVIEW BOARD

First

Last

Date of Participant Signature

MM/DD/YYYY

--NEXT PAGE—

If you selected “Parent of pediatric CD patient,” at the beginning of the survey please respond on behalf of the PATIENT

If you selected “Adult CD patient”/ “Related family member of CD patient”/ “Patient with related disease”/ “Healthy volunteer,” at the beginning of this survey, please enter YOUR information.

Please provide your current legal first name.

Please provide your current legal middle name. Please type in 0 if none.

Please provide your current legal last name.

Has your name changed since birth?

_Yes

_No

What is your date of birth?

MM/DD/YYYY

QUORUM REVIEW
APPROVED
INSTITUTIONAL
REVIEW BOARD

What was your physical sex at birth?

_Male

_Female

What city/municipality were you born in?

What country were you born in?

Please provide your current full mailing address below.

Street Address

Address Line 2

City

State / Province / Region

Postal / Zip Code

Country

_ Which of the following selections describes your ancestry? Please select all that apply.

_ American Indian or Alaska Native

_ Asian

_ Black or African American

_ Hispanic or Latino

_ Native Hawaiian or Other Pacific Islander

_ Caucasian, Non-Finnish Ancestry

_ Caucasian, Finnish Ancestry

QUORUM REVIEW
APPROVED
INSTITUTIONAL
REVIEW BOARD

_ I prefer not to say

_ Other (please specify)

Please enter your primary phone number where we may contact you if needed.

Please enter your email address.

--NEXT PAGE—

Would you consider yourself generally healthy?

_ Yes

_ No

Do you feel healthy today?

_ Yes

_ No

Please list any medical conditions or past diagnoses that we should be aware of or type in NA if this does not apply to you.

Please supply any reports or medical records associated with those diagnoses if you have any.

[FILE UPLOAD BUTTON]

Are you on any medications currently?

_ Yes

_ No

If yes, please list any drugs that you are currently taking.

QUORUM REVIEW
APPROVED
INSTITUTIONAL
REVIEW BOARD

--NEXT PAGE—

Thank you for completing our survey! We really appreciate your support in fighting back against Castleman disease.

The CDCN will review the information you have provided and determine how you can be involved in providing samples for research. We will be in touch once we have gathered enough information to be able to send you a kit in the mail with empty tubes or if we have any questions for you.

QUORUM REVIEW
APPROVED
INSTITUTIONAL
REVIEW BOARD

CDCN Biobank (Castleba ×

Secure | https://cdcn.wufoo.com/forms/cdcn-biobank-castlebank-consent-survey-1/

Apps | International News | Israel News | Social | Media | Reference | EMT | MD | DO | Jefferson | Home | Penn | Other bookmarks

CDCN Biobank (Castlebank) Consent Survey 1

Thank you for filling out this Survey, so that we will be able to include your samples in the BioBank.

If you are presented with a Survey Page that does not contain any questions, please select **"Next Page"** to continue through the Survey. That particular Survey Page does not contain any questions that pertain to you based on your prior responses.

If you have any questions, please call or email **1-215-614-0689 or biobank@castlemannetwork.org**

The following form will enable you to participate in the CDCN Biobank (Castlebank). Tissue samples such as blood or excess biopsy samples from patients will enable scientists to better characterize and understand Castleman disease and develop better treatments. Any blood or tissue donation will be used for high-impact research to battle this disease!

If you have any questions, please call or email**1-215-614-0689 or biobank@castlemannetwork.org**

Is this your first time filling out this Survey? *

- ○ First Time Participant
- ◉ Repeat Participant

If you do not remember if you have participated in the past, please call 1-215-614-0689

Repeat Participant Disclaimer

You have indicated that you are a **Repeat Participant**. If you have **NOT** competed this survey before, please select ***First Time Participant*** so that you will be presented with the appropriate Consent information.

If you do not remember if you have participated in the past, please call **1-215-614-0689**

Next Page

1 / 6

ENG 07:59 6/26/2017

QUORUM REVIEW
APPROVED
INSTITUTIONAL
REVIEW BOARD

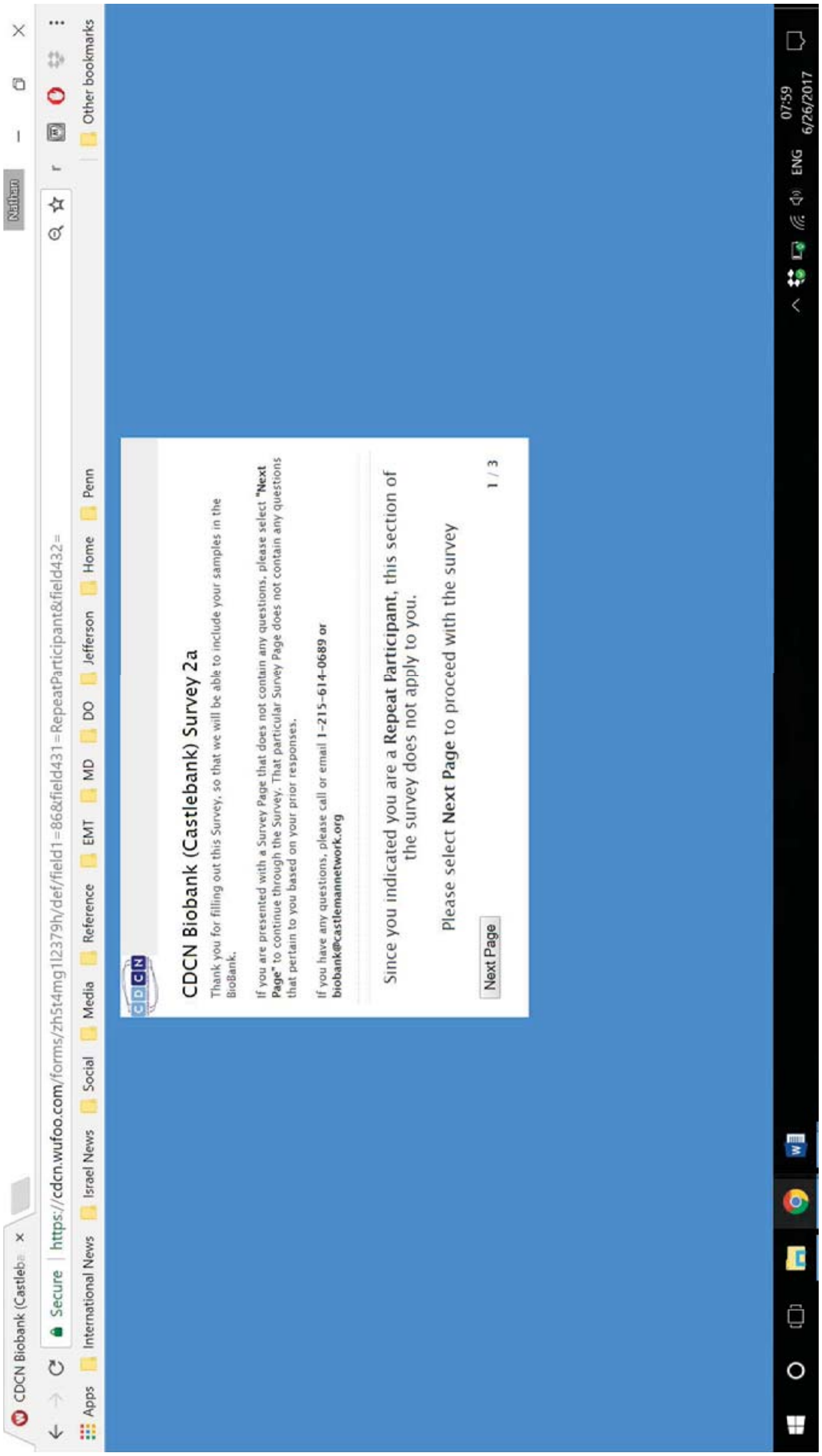

QUORUM REVIEW
APPROVED
INSTITUTIONAL
REVIEW BOARD

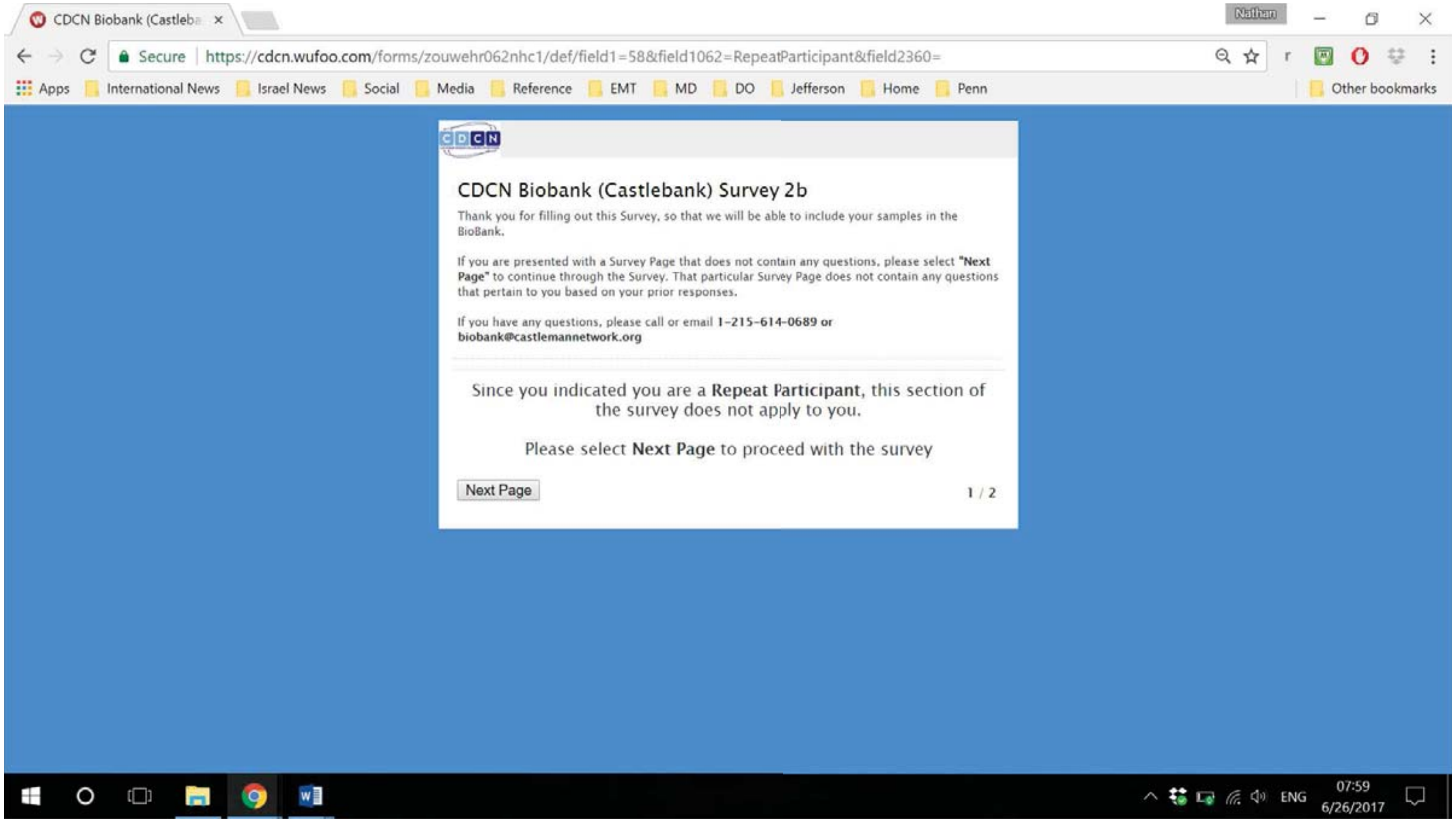

QUORUM REVIEW
APPROVED
INSTITUTIONAL
REVIEW BOARD

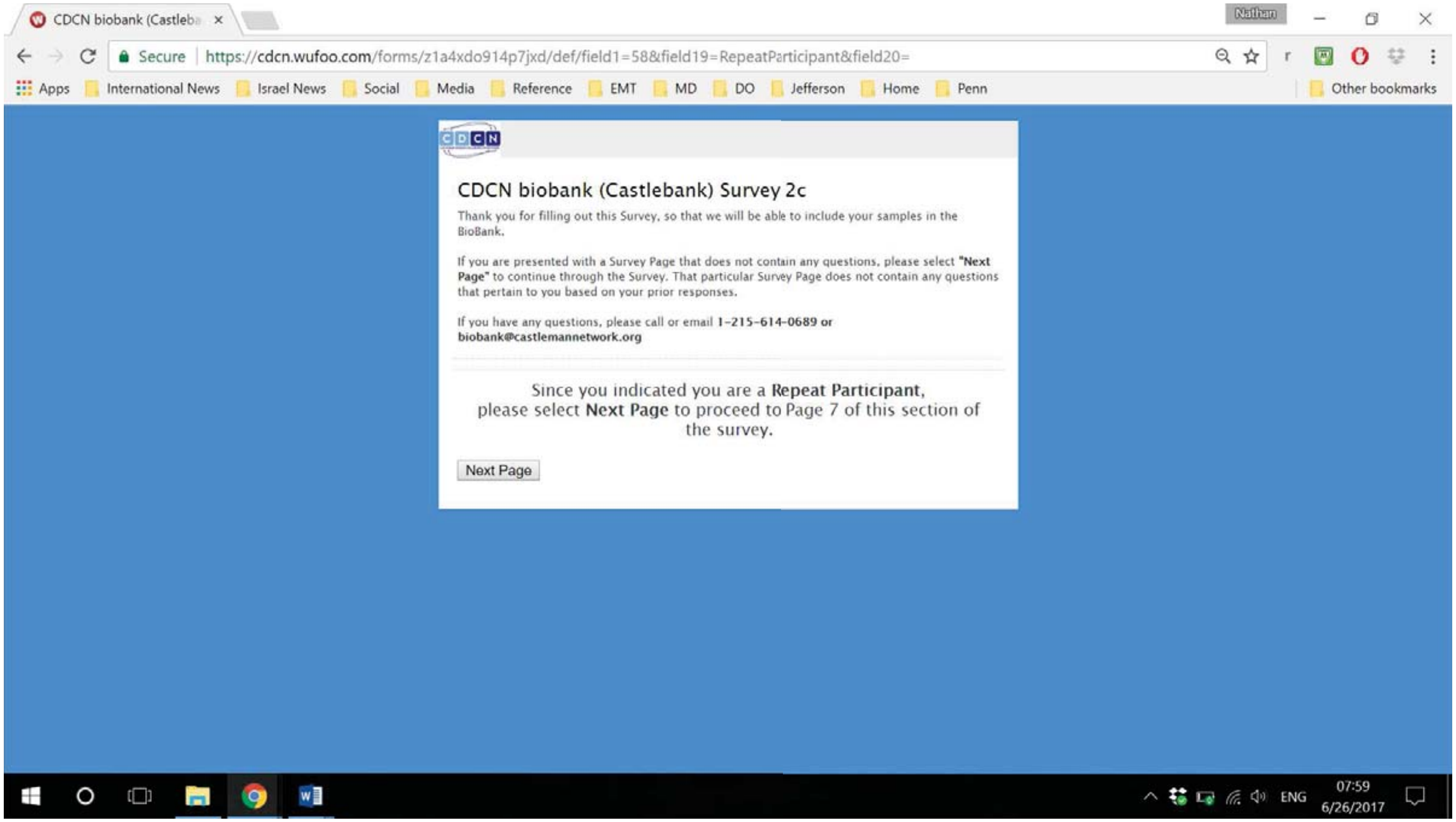

QUORUM REVIEW
APPROVED
INSTITUTIONAL
REVIEW BOARD

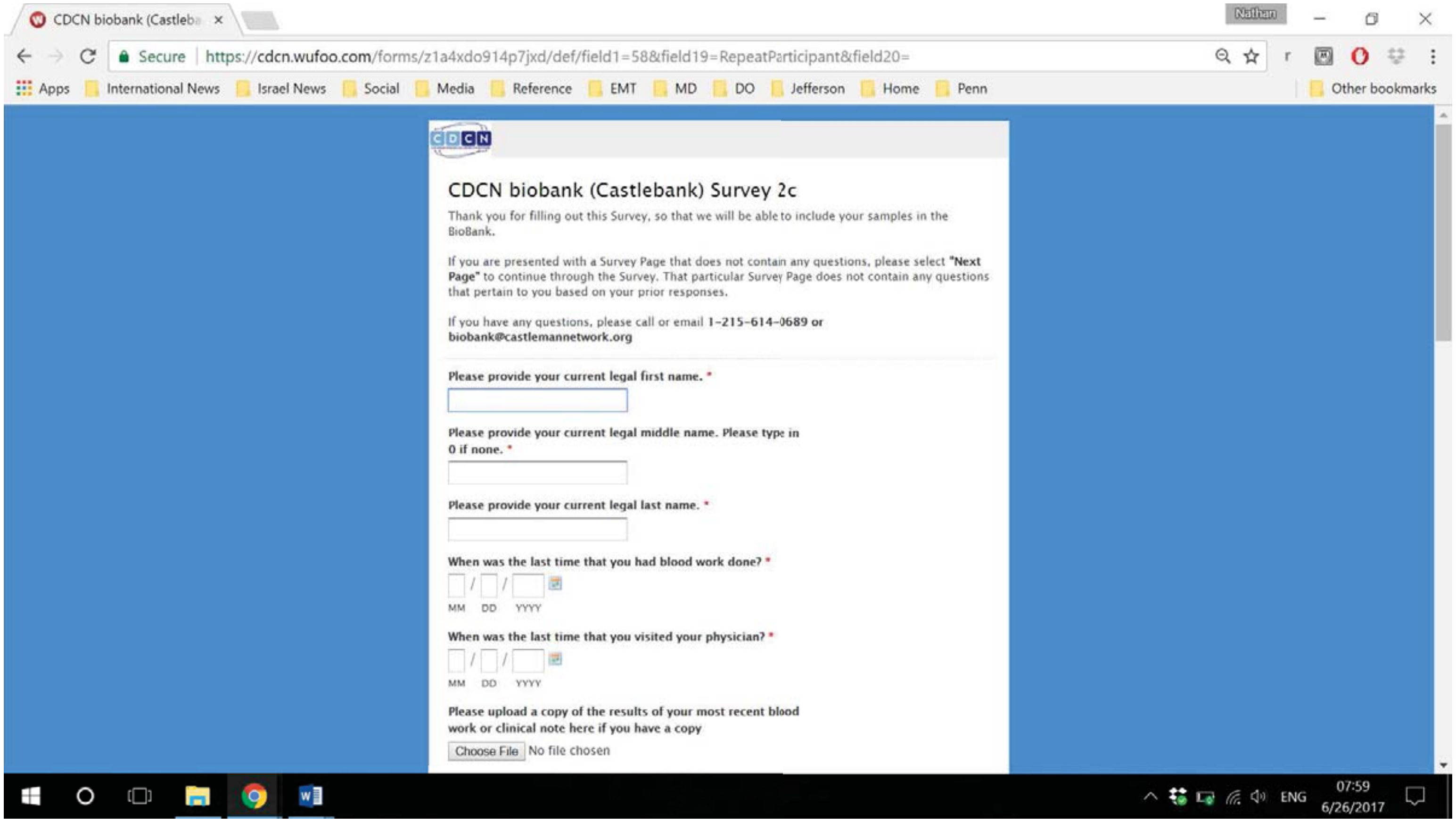

CDCN biobank (Castleb...

Secure | https://cdcn.wufoo.com/forms/z1a4xdo914p7jxd/def/field1=58&field19=RepeatParticipant&field20=

Apps | International News | Israel News | Social | Media | Reference | EMT | MD | DO | Jefferson | Home | Penn | Other bookmarks

CDCN

CDCN biobank (Castlebank) Survey 2c

Thank you for filling out this Survey, so that we will be able to include your samples in the BioBank.

If you are presented with a Survey Page that does not contain any questions, please select **"Next Page"** to continue through the Survey. That particular Survey Page does not contain any questions that pertain to you based on your prior responses.

If you have any questions, please call or email **1-215-614-0689 or biobank@castlemannetwork.org**

Please provide your current legal first name. *

Please provide your current legal middle name. Please type in 0 if none. *

Please provide your current legal last name. *

When was the last time that you had blood work done? *

MM / DD / YYYY

When was the last time that you visited your physician? *

MM / DD / YYYY

Please upload a copy of the results of your most recent blood work or clinical note here if you have a copy

Choose File No file chosen

07:59 6/26/2017

QUORUM REVIEW
APPROVED
INSTITUTIONAL
REVIEW BOARD

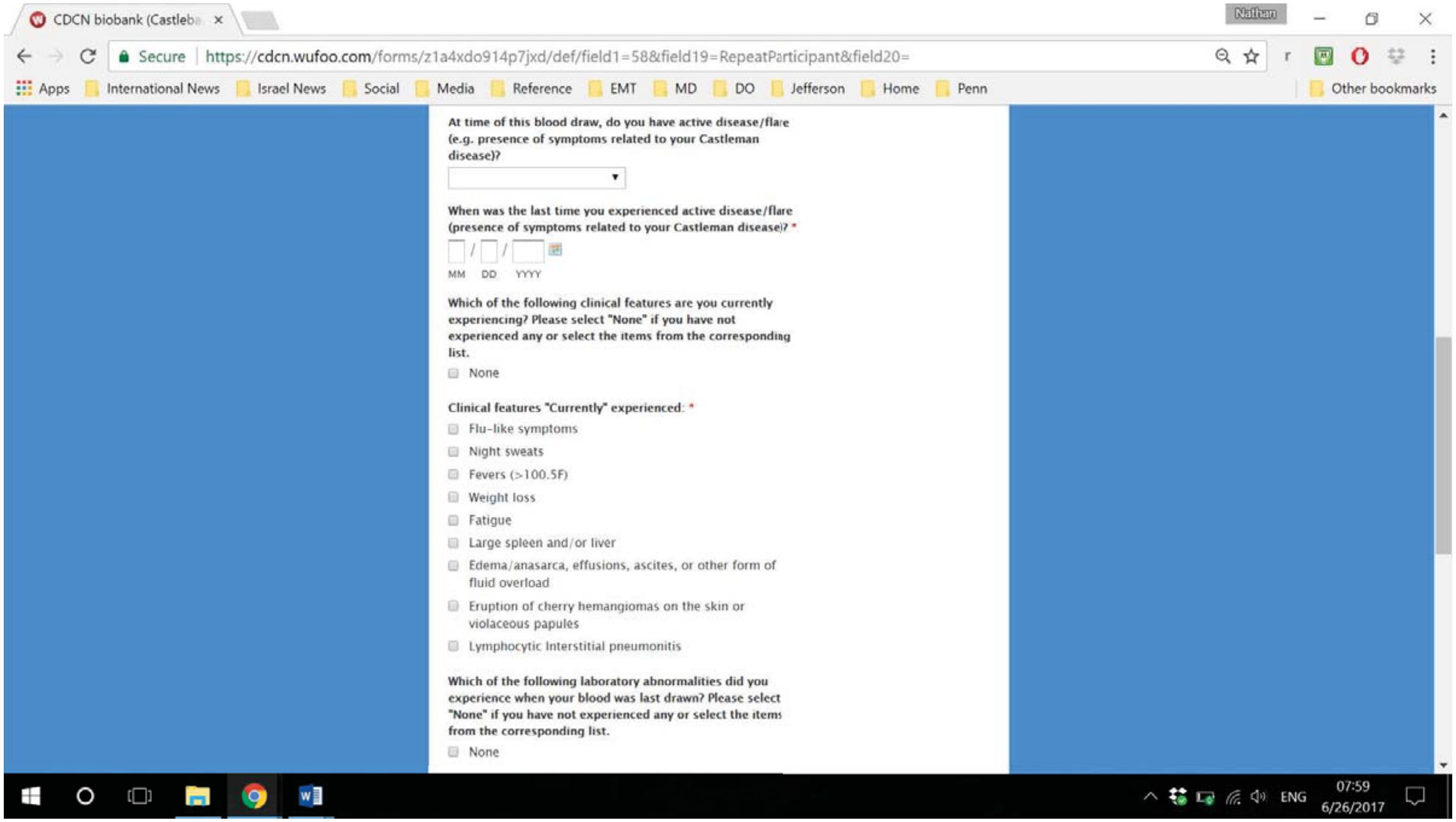

CDCN biobank (Castleba

Secure | https://cdcn.wufoo.com/forms/z1a4xdo914p7jxd/def/field1=58&field19=RepeatParticipant&field20=

Apps | International News | Israel News | Social | Media | Reference | EMT | MD | DO | Jefferson | Home | Penn | Other bookmarks

At time of this blood draw, do you have active disease/flare (e.g. presence of symptoms related to your Castleman disease)?

When was the last time you experienced active disease/flare (presence of symptoms related to your Castleman disease)? *

MM / DD / YYYY

Which of the following clinical features are you currently experiencing? Please select "None" if you have not experienced any or select the items from the corresponding list.

- None

Clinical features "Currently" experienced: *

- Flu-like symptoms
- Night sweats
- Fevers (>100.5F)
- Weight loss
- Fatigue
- Large spleen and/or liver
- Edema/anasarca, effusions, ascites, or other form of fluid overload
- Eruption of cherry hemangiomas on the skin or violaceous papules
- Lymphocytic Interstitial pneumonitis

Which of the following laboratory abnormalities did you experience when your blood was last drawn? Please select "None" if you have not experienced any or select the items from the corresponding list.

- None

ENG 07:59 6/26/2017

QUORUM REVIEW
APPROVED
INSTITUTIONAL
REVIEW BOARD

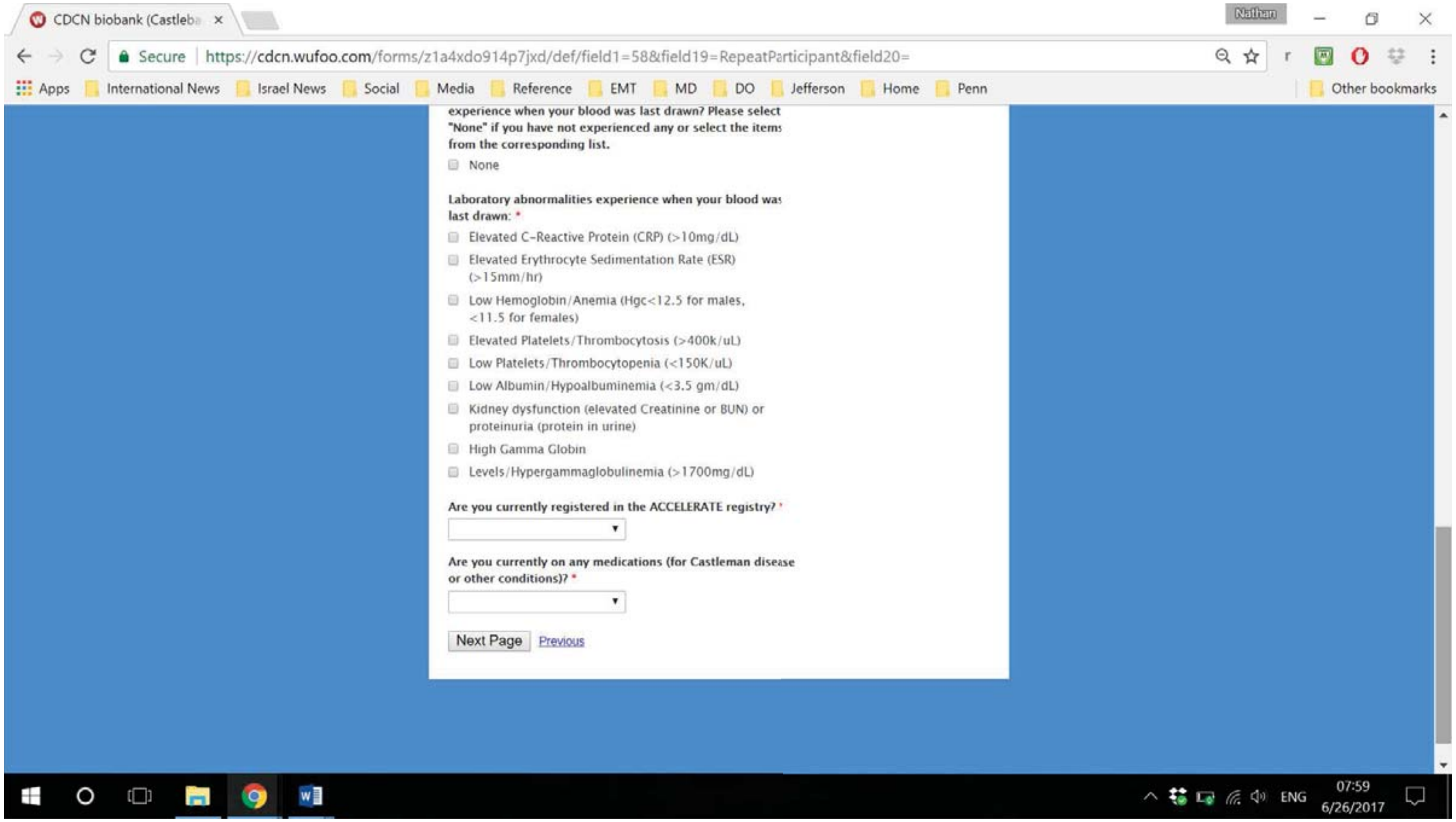

QUORUM REVIEW
APPROVED
INSTITUTIONAL
REVIEW BOARD

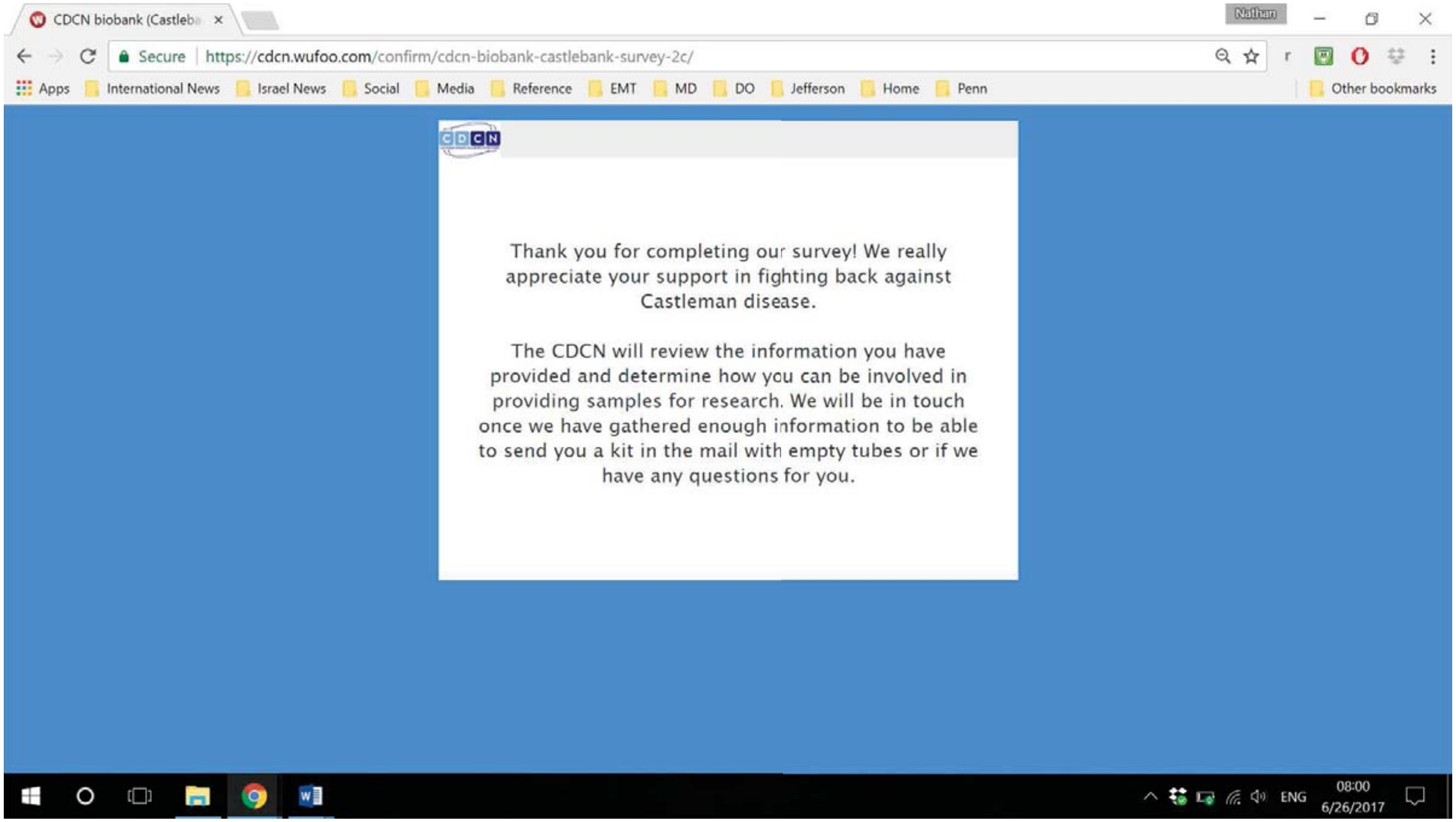

QUORUM REVIEW
APPROVED
INSTITUTIONAL
REVIEW BOARD

E

Email #1: PRELIMINARY INFORMATION (TO BE EMAILED TO "Confirmed" IN OUR DATABASE)

Title: We need your help. The answers to the unknowns of Castleman disease are within each of us.

Dear FIRST NAME,
As you know, Castleman disease is a very difficult diagnosis for patients and their loved ones. The lack of knowledge about the disease is a barrier to finding better treatment options and saving lives. We don't know what causes it, what immune cells are involved, or the most effective treatments. However, the Castleman Disease Collaborative Network (CDCN) has been working to change that by conducting research and we need your help! The CDCN is funding several projects, including the Castleman Genome Project, **but we need patient samples for these studies**. Participation as a patient in these studies would only involve mailing a blood or spit sample, asking your hospital to share excess samples from a previous biopsy, or enabling researchers to review your medical record data to better understand the disease.

Would you complete the following brief survey so that we can see if you or your loved one may be a good candidate for one of these studies? www.goo.gl/4aiyMz

If you are not a patient, would you please forward this to your loved one with Castleman disease?
Thank you so much!

Our top priority study is to perform genomic sequencing of patients and relatives to search for genetic variants that may be causing Castleman disease. We will be in touch if you or your loved one is a good fit, but please feel free to call us with questions: 610-304-0696.

Also, in a couple of months, we will establish a CDCN biobank called CastleBank, which will collect Castleman disease samples and disperse them to top researchers. We will be back in touch then about your sending in samples for that.

This summer, we will start a natural history registry called ACCELERATE, which will enable you to share your medical record data with researchers to gain insights into this disease. More info: http://www.cdcn.org/accelerate

This is a tough battle, but the answers are within each of us and together we can find better treatments for this deadly disease!

All our best,

[CDCN Member's Name] and David

[CDCN Member's Name]'s email signature

David Fajgenbaum, MD, MBA, MSc
Assistant Professor of Medicine, Translational Medicine & Human Genetics, University of

Pennsylvania
Co-Founder & Executive Director, Castleman Disease Collaborative Network

Email #2: DETAILED INFORMATION ABOUT DONATING (ONLY IF SOMEONE EMAILS TO ASK MORE ABOUT THE CASTLEMAN GENOME PROJECT—WE WANT PEOPLE WITH ALL SUBTYPES TO COMPLETE THE TISSUE SAMPLE INTEREST FORM AND NOT FEEL TURNED AWAY BECAUSE OF OUR CRITERIA)

Title: Detailed information about the Castleman Genome Project

Dear FIRST NAME,

Thank you for your interest in the Castleman Genome Project. The purpose of this study is to understand why the immune system becomes activated in Castleman disease. One hypothesis is that a genetic defect inhibits the ability to turn off the immune system response. To explore it, this study will examine the DNA of 10-15 CD patients, and their parents.

For this specific study, we are looking for participants that meet the following criteria:

- HHV-8-negative, multicentric subtype of Castleman disease
- Both parents of the patient are also willing and able to donate blood and saliva samples
- 35 years of age or younger
- Highly symptomatic at some point in the course of their disease that required hospitalization to support the patient
- Though not a requirement, we want to study Castleman disease patients that have another Castleman disease patient in their immediate or extended family

If you and your loved ones would like to participate in this study, please **complete the following brief survey so that we can see if you may be a good candidate? www.goo.gl/4aiyMz**

Even if you aren’t a good fit for this study, please complete the survey so that we can be in touch in the future about other studies.Please feel free to email or call us with questions: 610-304-0696.

This is a tough battle, but the answers are within each of us and together we can find better treatments for this deadly disease!

All my best,
[CDCN Member’s Name]

[CDCN Member’s Name]’s email signature

Email #3: NOT PARTICIPATING IN STUDY (PATIENT REPLIED TO SAY THAT HE/SHE IS NOT INTERESTED IN CONTRIBUTING SAMPLES FOR RESEARCH OR COMPLETING THE TISSUE INTEREST FORM)

Title: Other ways to help fighting Castleman disease

Dear FIRST NAME,

Thank you for your interest in joining the fight to take down Castleman Disease. We understand that you're not interested in providing samples for research, and there are other ways that you can contribute to help us to improve understanding and treatment of Castleman disease.

We are conducting research studies around the world and need funding to make these happen. Please visit http://www.cdcn.org/about-us/current-priorities for a listing of our research priorities focused on identifying what causes Castleman disease, which type of immune cells are involved, and what treatments are effective for patients. If you want to help to make these studies happen, please donate at http://www.cdcn.org/donate.

You can also become a Castleman Warrior to help raise funds for life-saving research. Read a recent story about one of our inspirational patients, who is also a Castleman Warrior: http://www.cdcn.org/PatientPowered.

Please visit http://www.cdcn.org/get-involved or email info@castlemannetwork.org for ways you and your loved ones can get involved.

All my best,
[CDCN Member's Name]

[CDCN Member's Name]'s email signature

Email #4: ACCEPTED INTO STUDY

Title: Castleman Genome Project – You have been selected to participate

Dear FIRST NAME,

Thank you for completing the tissue sample donation survey. We are pleased to inform you that, based on your survey information, you may be a good candidate for the Castleman Genome Project, which may lead to the identification of the genetic drivers of Castleman disease! There are just a few simple steps for you to participate:

1) We need to connect you with the principal investigator (Dr. Minji Byun) of the study so that she can assess your eligibility criteria and consent you. Will you reply to this email stating that we can share your name and contact information with Dr. Byun?

2) Dr. Minji Byun will contact you to discuss your eligibility, consent you, and send you a sample collection kit.

3) You and your family members will follow the instructions in the sample collection kit to collect your saliva and blood samples and then ship the samples to Dr. Byun in St. Louis (at no cost).

We truly appreciate your participation in this study and thank you in advance for your cooperation. If you have any questions, please feel free to contact us at 610-304-0696.

All my best,
[CDCN Member's Name]

[CDCN Member's Name]'s email signature

Email #1: PRELIMINARY INFORMATION (TO BE EMAILED TO the "not sure's" IN OUR DATABASE)

Title: We need your help. The answers to the unknowns of Castleman disease are within each of us.

Dear FIRST NAME,
Thank you for completing the registration process on www.CDCN.org for you or a loved one with Castleman disease. You listed "not sure" next to your diagnosis and we wanted to clarify with you about your current diagnosis. Would you please reply to this email to let us know what most accurately describes you or your loved one (if a loved one, please email us his or her name):

a. I (or my loved one) have been diagnosed by a doctor with unicentric Castleman disease
b. I (or my loved one) have been diagnosed by a doctor with HHV-8-positive multicentric Castleman disease
c. I (or my loved one) have been diagnosed by a doctor with HHV-8-negative or "idiopathic" multicentric Castleman disease
d. I (or my loved one) have been diagnosed by a doctor with Castleman disease but I'm not sure if it is unicentric, HHV-8-positive multicentric, or HHV-8-negative multicentric
e. I (or my loved one) have not been diagnosed by a doctor with Castleman disease but my doctors are considering that I may have Castleman disease.

If you answered A, B, C, or D, you know that Castleman disease is a very difficult diagnosis for patients and their loved ones. The lack of knowledge about the disease is a barrier to finding better treatment options and saving lives. However, the Castleman Disease Collaborative Network (CDCN) has been working to change that by conducting research and **we need patient samples for these studies**!

Participation as a patient in these studies would only involve mailing a blood or spit sample, asking your hospital to share excess samples from a previous biopsy, or enabling researchers to review your medical record data to better understand the disease.

Would you complete the following brief survey so that we can see if you or your loved one may be a good candidate for one of these studies? www.goo.gl/4aiyMz

If you are not the patient, would you please forward this to your loved one with Castleman disease?
Thank you so much!

Please feel free to call us with questions: 610-304-0696.

This is a tough battle, but the answers are within each of us and together we can find better treatments for this deadly disease!

All our best,

[CDCN Member's Name] and David

[CDCN Member's Name]'s email signature

David Fajgenbaum, MD, MBA, MSc
Assistant Professor of Medicine, Translational Medicine & Human Genetics, University of Pennsylvania
Co-Founder & Executive Director, Castleman Disease Collaborative Network
